# Quality improvement and practice-based research in sleep medicine using structured clinical documentation in the electronic medical record

**DOI:** 10.1186/s41606-019-0038-2

**Published:** 2020-01-02

**Authors:** Demetrius M. Maraganore, Thomas Freedom, Kelly Claire Simon, Lori E. Lovitz, Camelia Musleh, Richard Munson, Nabeela Nasir, Smita Patel, Joya Paul, Mari Viola-Saltzman, Steven Meyers, Richard Chesis, Laura Hillman, Samuel Tideman, Anna Pham, Rosa Maria Vazquez, Roberta Frigerio

**Affiliations:** 1 Department of Neurology, NorthShore University HealthSystem, 2650 Ridge Ave, Evanston, IL 60201, USA.; 2 Department of Neurology, University of Florida, 1149 Newell Drive, Gainesville, FL 32611, USA.; 3 Health Information Technology, NorthShore University HealthSystem, 4901 Searle Parkway, Skokie, IL 60077, USA.

**Keywords:** Electronic medical record, Sleep disorders, Structured clinical documentation support, Clinical decision support, Best practices

## Abstract

**Background:**

We developed and implemented a structured clinical documentation support (SCDS) toolkit within the electronic medical record, to optimize patient care, facilitate documentation, and capture data at office visits in a sleep medicine/neurology clinic for patient care and research collaboration internally and with other centers.

**Methods:**

To build our SCDS toolkit, physicians met frequently to develop content, define the cohort, select outcome measures, and delineate factors known to modify disease progression. We assigned tasks to the care team and mapped data elements to the progress note. Programmer analysts built and tested the SCDS toolkit, which included several score tests. Auto scored and interpreted tests included the Generalized Anxiety Disorder 7-item, Center for Epidemiological Studies Depression Scale, Epworth Sleepiness Scale, Pittsburgh Sleep Quality Index, Insomnia Severity Index, and the International Restless Legs Syndrome Study Group Rating Scale. The SCDS toolkits also provided clinical decision support (untreated anxiety or depression) and prompted enrollment of patients in a DNA biobank.

**Results:**

The structured clinical documentation toolkit captures hundreds of fields of discrete data at each office visit. This data can be displayed in tables or graphical form. Best practice advisories within the toolkit alert physicians when a quality improvement opportunity exists. As of May 1, 2019, we have used the toolkit to evaluate 18,105 sleep patients at initial visit. We are also collecting longitudinal data on patients who return for annual visits using the standardized toolkits. We provide a description of our development process and screenshots of our toolkits.

**Conclusions:**

The electronic medical record can be structured to standardize Sleep Medicine office visits, capture data, and support multicenter quality improvement and practice-based research initiatives for sleep patients at the point of care.

## Background

Many sleep disorders have complex symptomatology and variable evolution. Carefully documenting the severity of disease, the frequency of symptoms, impact on health and quality of life, and response to treatment is essential to guiding the efficacy and safety of disease management. With the advent of the electronic medical record (EMR), it became possible to process and store data for easy evaluation and comparison. The EMR also allows immediate access to longitudinal health information, improving the quality, safety, and efficiency of patient care (Esper et al. 2010). The EMR can provide information on therapy outcomes (Ballard et al. 2015), track quality of care (Fitzsimons et al. 2013), screen for neurological disorders (Wray et al. 2014), and improve communication between specialists and primary care physicians (Zuchowski et al. 2015).

The American Academy of Neurology (AAN) has proposed quality measures for the care of adults with neurological disease (Cheng 2010; Fountain et al. 2011; Miller et al. 2013; Odenheimer et al. 2013; England et al. 2014), and guidelines for restless legs syndrome/Willis-Ekbom disease (RLS/WED) are under development (Guidelines Projects in Process, 2019). The American Academy of Sleep Medicine recently published quality measures (Trotti et al. 2015). Other guidelines for RLS/WED have been published (Allen et al. 2014; Picchietti et al. 2015; Garcia-Borreguero et al. 2016; Winkelman et al. 2016). Few studies in sleep and neurology are incorporated into EMRs (Alexander et al. 2016; Stubberud et al. 2019; Mulvey et al. 2018). A challenge to EMR documentation is that clinical data are not captured discretely, making it difficult to report performance and assess quality improvement opportunities. Structured clinical documentation support offers a solution to this problem, allowing data to be captured discretely, and making it easier to report performance.

Sleep medicine is also challenged by a lack of comparative effectiveness research. There are several approved treatments for common sleep disorders, such as RLS/WED, but it is unknown which ones are superior in efficacy and tolerability and for which patient subgroups. Traditional clinical trials enroll selected patients, use surrogate measures, follow patients for short periods, and generalize poorly to clinical practice (Tunis et al. 2003; Vickers and Scardino 2009; Patsopoulos 2011; Chalkidou et al. 2012). Data captured in the EMR could be used to identify eligible patients, assign treatments, and measure outcomes at the point of care (Staa et al. 2012). To address these unmet needs, we participated in a department-wide project addressing quality improvement and practice-based research in neurology by building a structured clinical documentation support (SCDS) and clinical decision support (CDS) toolkit within our EMR. We present a description of our process for developing a sleep toolkit to support Best Practices and provide some basic descriptive data demonstrating our discrete data collection. As an example, we present RLS/WED as one of the many sleep disorders for which our toolkit collects data. RLS/WED serves as a useful example because it is well defined, has a genetic basis, and poorly understood etiology requiring further study.

## Methods

The NorthShore University HealthSystem (NorthShore) Sleep program includes fourteen physicians (six pulmonologists and eight neurologists). The toolkit is used by sleep specialists from the Department of Neurology practicing at four hospitals, and at six outpatient sites in the northern suburbs of Chicago, IL.

To build our SCDS toolkit, we had to develop the content of the note, optimize the EMR (Epic Systems Corporation) to reflect the content, and implement the toolkit in a test environment. We previously illustrated our seven-stage, stepwise progression of quality improvement and practice-based research using the EMR. Briefly, the department’s stepwise progression consists of seven steps: 1. Development and implementation of structured clinical documentation support (SCDS) EMR tools; 2. Enrollment reports of subjects encountered; 3. Data quality reports to identify missing data; 4. Descriptive reports of group characteristics such as patient reported and physcian assessment measures; 5. Quality improvement projects (baseline data); 6. Quality improvement projects (using clinical decision support tools built into the EMR to hardwire patient safety and improved outcomes); 7. Dissemination of tools and sharing of data via the NPBRN (Maraganore et al. 2016).

The sleep neurologists met every 2 weeks to standardize the progress note content to support Best Practices in treating patients with sleep disorders. We reviewed the pertinent medical literature, AAN guidelines (Guidelines, 2019), National Institute of Neurological Diseases and Stroke Common Data Elements (NINDS/NIH, 2015), the American Academy of Sleep Medicine (AASM) (Aurora et al. 2012), and the International Restless Legs Syndrome Study Group (IRLSSG) guidelines (Garcia-Borreguero et al. 2013). We included the Center for Epidemiologic Studies Depression (CES-D) scale (Radloff 1977), General Anxiety Disorder 7 item (GAD-7) scale (Spitzer et al. 2006), Pittsburgh Sleep Quality Index (PSQI) (Buysse et al. 1989), Epworth Sleepiness Scale (ESS) (Johns 1991), Insomnia Severity Index (ISI) (Morin 1993; Bastien et al. 2001), and International Restless Legs Syndrome Study Group Rating Scale (IRLSRS) (Walters et al. 2003). We designed workflows (the order and assignment of tasks to a care team (medical assistant, nurse, sleep neurologist)) according to the level of training and progress notes (the order and layout in which the content would write). Patients may also fill in their questionnaires online ahead of the office visit through our online patient portal. This process does not lengthen the office visit and ensures collection of all the necessary data. We limited the medical assistant and/or nurse assessments to 15 min each and the neurologist assessment to 60 min.

After deciding on the content, we conducted meetings with programmers from NorthShore’s EMR Optimization team every 2 weeks. They built an SCDS and CDS toolkit that included navigators (a sidebar index of processes to choose from), electronic forms (which had the ability to auto-score and auto-interpret), and summary flow sheets. We included free text fields to allow for additional information. All patients with sleep disorders were evaluated using the SCDS. The SCDS and CDS toolkit were also designed to promote patient safety (by alerting physicians of untreated anxiety or depression and directing them to a mental health orders set).

Finally, we tested the SCDS and CDS toolkit in the EMR’s development environment, made revisions, and moved the toolkit to the EMR’s production environment. The project team continued to meet every 2 weeks to make revisions based on patient encounters. Our toolkits focused on outpatient visits using the EMR’s ambulatory environment. (Screenshots of the SCDS and CDS tools appear in Additional file 1).

After toolkit implementation, we met every 2 weeks with programmers specialized in extracting, transforming, and loading data from the EMR’s data repository to sleep-specific data marts in NorthShore’s Enterprise Data Warehouse (EDW). The EDW programmers created enrollment reports for tracking patients and produced data quality reports indicating which required data was missing from office visits. These data quality reports were distributed to the care team monthly. Data not cleaned within 3 months were archived as permanently missing, and those data were no longer listed in subsequent reports. The care team learned where they were error prone from the data quality reports, and they remediated their use of the toolkits. When systematic errors occurred for many providers, the teams had the opportunity to improve their use of the toolkits or to request optimizations or a change in data requirements. The monthly reports produced only a few or no data checks per provider once the project was established.

## Results

### Baseline descriptives

To demonstrate what our toolkit effectively captures, as of May 1, 2019, we have evaluated 18,105 patients with our Sleep toolkit, including up to 836 fields of data per office visit (example screenshots in Additional file 1). Our toolkit generates monthly reports, such as Table 1, which shows the sleep diagnoses of patients seen at initial visit. For each of these patients, we collected detailed information related to their demographics. We also collected detailed information related to their specific symptoms, sleep medication use, and family history of sleep related disorders. An example of a detailed, descriptive report related to these patients is shown in Additional file 2. Lastly, we collected several score test measures that can be evaluated longitudinally as we follow these patients (Additional file 3).

## Discussion

We employed an SCDS and CDS toolkit built within our EMR for quality improvement and outcomes research. We have demonstrated the feasibility of incorporating these toolkits into clinical care and capturing high-level patient data. Additionally, we capture longitudinal data on patients by continued use of the toolkits at annual visits. Understanding how measures of patient’s wellbeing change over time is informative for planning appropriate care across disciplines.

As a clinical next step, we will use the data collection to aid in decisions at the point of care. Specifically, we are creating electronic pop-up boxes (best practice advisories [BPAs]) that fire at the point of care whenever a quality improvement opportunity is identified, based on data captured by the EMR. We have already implemented these to detect and manage depression or anxiety in the sleep visit. We implemented a BPA based on a patient’s CES-D and GAD-7 score. If the patient screens positive, an alert ‘pops’ up requiring the physician to place an order (medication or referral) or defer (and a comment as to the reason is required). As a quality measure, we are assessing the frequency of antidepressant or anxiolytic medication order and/or psychiatry referral order for those that screen positive. We are planning additional BPAs, specific to sleep patients. One application could be if a patient has RLS/WED and the ferritin level has not been checked in the past year, a BPA will fire and present a mouse-click option to place an order or defer (which will cascade and prompt selection of a reason for deferral). After implementing the BPA, we will track improvements in documentation of ferritin levels versus benchmark data. In another application, we could screen for impulse control disorder and augmentation. If a patient screens positive, we could alter management of dopamine agonists.

Several research applications could be undertaken using this routinely collected clinical data. We are currently conducting a DNA biobanking study, in which enrollment is prompted by a BPA that scans the EMR for eligibility criteria. Eligible patents are identified automatically at the point of care. When appropriate, they can then be consented into the study. DNA information in addition to clinical data will allow novel studies of biomarkers and clinical features as it relates to disease progression, treatment response and outcomes. Another future opportunity is to conduct pragmatic trials using the EMR. For example, we could compare a dopamine agonist versus an alpha-2 delta ligand to evaluate outcomes such as RLS/WED severity, augmentation rates, and effects on scales such as the CES-D and ESS. BPAs would trigger enrollment of eligible subjects and prompt random assignment of equivocal medications, at the point-of-care, using the data captured by our SCDS and CDS toolkit. We have already implemented this design successfully for other projects in the Department of Neurology.

The Department of Neurology at NorthShore University HealthSystem (NorthShore) includes 40 neurologists practicing at 4 hospitals and 8 outpatient sites in the north suburbs of Chicago, IL. The neurologists include generalists and subspecialists in epilepsy, neurodegenerative disorders, multiple sclerosis, neuromuscular disorders, neuro-oncology, sleep disorders, and stroke. Our toolkit creates an efficient division of labor among the medical staff that renders the process of obtaining all necessary data more clear and efficient.

Our toolkit also facilitates collaboration within our eleven neurology subspecialties that all have access to the data. The data collected can also be shared with other specialties and centers if they are willing to create a standardized toolkit. While our toolkit is currently limited to neurology subspecialities that use the same EMR, potential future applications include expanding to other subspecialties and EMR platforms.

To improve quality and conduct novel practice-based research, we are also sharing our sleep toolkit and collaborating with other departments of neurology that use the same EMR (Epic). With funding support from the Agency for Healthcare Research and Quality, the NorthShore Neurological Institute created a Neurology Practice-Based Research Network (NPBRN) using the EMR. All 15 participating sites have agreed to implement SCDS and CDS toolkits for up to 11 neurological indications (including sleep disorders), and to share de-identified data collaboratively. The NorthShore site will manage the data and provide monthly participation and data quality reports as well as quarterly descriptive cohort and quality improvement reports, which will allow us to analyze the data for demographic and clinical characteristics in diverse patient populations and to conduct multi-center research studies.

While there are many benefits to incorporating SCDS and CDS into clinical practice, there are also some limitations. The structured questions may not be asked in the same manner by different examiners. The patients’ answers may be subjective or recorded in a biased manner by the examiner. Additionally, patients’ interpretation of self-entered questionnaires may vary. There is a learning curve to using the toolkits and they require ‘standardized’ answers. However, there is always the opportunity to enter free text. In our experience, once fully implemented, the toolkits do not require additional face-to-face time and include features that can increase the efficiency of the office visit, such as the previously mentioned clearly assigned tasks to the medical staff.

Over time, we have rendered the toolkit more efficient by eliminating some of our initial questionnaires that we found redundant and unnecessary, shortening the data collection process. To address the challenge of data collection prior to the physician visit, we streamlined the process, assigning the medical staff to specific data collection tasks in addition to having the patient fill out some questionnaires online prior to coming in the office. These changes have improved our workflow, while ensuring that our data collection process is complete and efficient.

## Conclusions

In conclusion, SCDS toolkits, as well as CDS features, may be used to standardize a sleep medicine office visit. Workflows may be optimized to deliver patient care, and progress notes transformed from unstructured text to structured documents with discrete data points, easily captured in the EMR. The toolkits can be built to enable physicians to approach common diseases according to AAN quality guidelines and support quality initiative projects. The toolkits can also aid in the enrollment of research by identifying eligible patients at the point of care. Furthermore, the data can be queried for research questions such as whether the IRLS score predicts whether a patient will be treated or whether the ferritin level predicts benefit from treatment. Overall, we anticipate that these SCDS and CDS toolkits will improve the quality of sleep medicine care and facilitate practice-based research.

## Supplementary Material

Additional file 1 **Additional file 1**. Screenshots of SDCS toolkit within the EMR,© 2018 EPIC Systems Corporation, used with permission

Additional file 2 **Additional file 2**. Complete descriptive report of patients at initial visit.

Additional file 3 **Additional file 3**. Score tests distributions, correlations, and principal component analysis of patients at initial visit.

## Figures and Tables

**Table 1:** Diagnosis of Sleep Clinic Patients at initial visit

Sleep Related Breathing Disorders	11,988
Insomnia	2760
Sleep Related Movement Disorders	1708
Hypersomnias of Central Origin	646
Parasomnias	415
Circadian Rhythm Sleep Disorders	372

Note: Patient may have been diagnosed with more than one sleep disorder

## Abbreviations

AAN: American Academy of Neurology
AASM: American Academy of Sleep Medicine
BPA: Best practice advisory
CDS: Clinical decision support
CES-D: Center for Epidemiologic Studies Depression scale
DNA: Deoxyribonucleic acid
EDW: Enterprise Data Warehouse
EMR: Electronic Medical Record
ESS: Epworth Sleepiness Scale
FDA: U.S. Food and Drug Administration
GAD-7: General Anxiety Disorder 7 item scale
IRLSRS: International Restless Legs Syndrome Study Group Rating Scale
IRLSSG: International Restless Legs Syndrome Study Group
ISI: Insomnia Severity Index
NINDS: National Institute of Neurological Diseases and Stroke Common Data Elements
NPBRN: Neurology Practice-Based Research Network
PSQI: Pittsburgh Sleep Quality Index
RLS/WED: Restless Legs Syndrome/Willis Ekbom Disease
SCDS: Structured clinical documentation support
SNP: Single nucleotide polymorphisms
