# Supplementary material for: Quality improvement and practice-based research in sleep medicine using structured clinical documentation in the electronic medical record
Source: Sleep Sci Pract. Author manuscript; Available in PMC 2020 May 11. (PMC7213673; doi:10.1186/s41606-019-0038-2)
Supplement: Additional file 1 — Screenshots of SDCS toolkit within the EMR,© 2018 EPIC Systems Corporation, used with permission [file NIHMS1574724-supplement-Additional_file_1.pdf]

DodoNA Sleep – Medical Assistant Section

Biometrics

Biometrics - Biometrics

Show: All Choices

Values By

Biometrics

Neck circumference (inches)	15.5	Waist circumference (inches)	34
Hip circumference (inches)	38	Waist hip ratio	0.89

Restore | Close F9 | Cancel | Previous F7 | Next F8

©2019 Epic Systems Corporation. Confidential.

ESS

EPWORTH - Epworth Sleepiness Scale (ESS)

Show: All Choices

Values By

Testing Status

Was test performed? **Yes** | Unable to perform | Patient refused

EPWORTH SLEEPINESS SCALE

Item	Selected (0 / 1 / 2 / 3)
Sitting and reading	1
Watching TV	1
Sitting inactive in a public place	0
Being a passenger in a motor vehicle for an hour or more	0
Lying down in the afternoon	0
Sitting and talking to someone	0
Sitting quietly after lunch (no alcohol)	2
Stopped for a few minutes in traffic while driving	0

Total score: 4 | Epworth: Questions answered (out of 8): 8

Epworth score indicates that patient is considered to be: Not Sleepy

Comments:

©2019 Epic Systems Corporation. Confidential.

DodoNA Sleep – Medical Assistant Section

PSQI

Pittsburgh Sleep Quality Index

PITTSBURGH SLEEP QUALITY INDEX (PSQI)

Testing Status

Was test performed?
Yes | Unable to perform | Patient refused

Instructions: The following questions relate to your usual sleep habits during the past month ONLY. Your answers should indicate the most accurate reply for the MAJORITY of days and nights in the past month. Please answer all questions.

Sleep Habits

1. During the past month, what time have you usually gone to bed at night?

Hour: 01 02 03 04 05 06 07 08 09 10 11 12
Minute: 00 15 30 45
Period: AM / PM

2. During the past month, how long (in minutes) has it usually taken you to fall asleep each night?

35 NUMBER OF MINUTES

3. During the past month, what time have you usually gotten up in the morning?

Hour: 01 02 03 04 05 06 07 08 09 10 11 12
Minute: 00 15 30 45
Period: AM / PM
Same Day

4. During the past month, how many hours of ACTUAL SLEEP did you get at night? (This may be different than the number of hours you spent in bed.)

7 HOURS OF SLEEP PER NIGHT

For each of the remaining questions, check the one best response. Please answer ALL questions.

5. During the past month, how often have you had trouble sleeping because you . . .

5a) Cannot get to sleep within 30 minutes:
Not during the past month | Less than once a week | Once or twice a week | Three or more times a week

5b) Wake up in the middle of the night or early morning:

©2019 Epic Systems Corporation. Confidential.

ISI

Insomnia Severity Index - Insomnia Severity Index

Show: All Choices

Values By

Testing Status

Was test performed? Yes | Unable to perform | Patient refused

Please rate the CURRENT (i.e. LAST 2 WEEKS) SEVERITY of your insomnia problem(s).

1. Difficulty falling asleep: 0=None | 1=Mild | 2=Moderate | 3=Severe | 4=Very severe
2. Difficulty staying asleep: 0=None | 1=Mild | 2=Moderate | 3=Severe | 4=Very severe
3. Problem waking up too early: 0=None | 1=Mild | 2=Moderate | 3=Severe | 4=Very severe
4. How SATISFIED/DISSATISFIED are you with your CURRENT sleep pattern?: 0=Very satisfied | 1=Satisfied | 2=Moderately satisfied | 3=Dissatisfied | 4=Very dissatisfied
5. How NOTICEABLE to others do you think your sleep problem is in terms of impairing the quality of your life?: 0=Not at all noticeable | 1=A little | 2=Somewhat | 3=Much | 4=Very much noticeable
6. How WORRIED/DISTRESSED are you about your: 0=Not at all worried | 1=A little | 2=Somewhat | 3=Much | 4=Very much worried

©2019 Epic Systems Corporation. Confidential.

DodoNA Sleep – Registered Nurse Section

Sleep Study

Sleep Study - Sleep Study

Time taken: 1225 | 9/29/2016

Show: Last Filed | All Choices

Values By

Sleep Study

Sleep study?	Yes / No	Yes taken 1 month ago
Type of sleep study	Actigraphy / Home Sleep Test / Multiple Sleep Latency Test / Nocturnal Polysomnography / Oral Titration Study / PAP Titration Study / Split Night Study / Suggested Immobilization Test	Nocturnal Polysomnography taken 1 month ago

Nocturnal Polysomnography (PSG)

PSG (year performed)	Don... / 2025 / 2024 / 2023 / 2022 / 2021 / 2020 / 2019 / 2018 / 2017 / 2016 / 2015 / 2014 / 2013 — 2011 taken 1 month ago		
PSG (total sleep time in minutes)	408 minutes taken 1 month ago	PSG (sleep efficiency)	86 % taken 1 month ago
PSG (arousal Index)	16 events/hour taken 1 month ago	PSG (apnea hypopnea index)	3 events/hour taken 1 month ago
PSG (lowest SaO2)	90 % taken 1 month ago	PSG (periodic limb movement index)	22 events/hour taken 1 month ago
PSG (periodic limb movement arousal index)			

©2019 Epic Systems Corporation. Confidential.

CPAP DL Results

CPAP DL Results - Sleep CPAP DL results

Time taken: 1059 | 2/22/2017

Values By

› Sleep Study

› Other Info

PAP COMPLIANCE

DME	
METHOD	CPAP / AutoPAP / BiPAP / Auto Bi... / ASV / VPAP / Other
Type of Interface	Pillows / Nasal / Full face
Date Range	
Percentage Days with Use	
Maximum Use	
Average Use (days used) Hrs Mins	
Percent Days over 4 Hrs %	
Average Pressure (90%tile): Cm H2O (whole number or fraction)	
Peak Pressure	
Average Time in Large Leak per Day: Hrs Mins	
Average AHI:	
Triggered Breaths (ASV only)	
1 Month download	Yes / No
3 Month Download	Yes / No
6 Month Download	Yes / No
12+ Month Download	Yes / No
Satisfaction with DME	1 / 2 / 3 / 4 / 5

©2019 Epic Systems Corporation. Confidential.

Current Symptoms

Current Symptoms - Current Sleep Disruptors

Show: Last Filed | All Choices

Values By

Do any of the following occur during sleep or affect your sleep

Current sleep disruptors	Anxiety · Bed partner (snoring, movements) · Irritability · Noise · Pain · Pets · Racing thoughts · Room temperature · Sadness/depression · Uncomfortable bed · Other · None<br>**Other** taken 1 month ago
Current symptoms pertaining to breathing	Dry mouth;Snoring;<br>Awakening to urinate · Chest pain · Choking · Dry mouth · Gasping · Heart racing · Heart slowing<br>**Dry mouth; Snoring** taken 1 month ago
Current symptoms pertaining to limb discomfort or abnormal movements	Leg cramps · Leg discomfort · Leg movements · Muscular tension · Teeth grinding · Other unusual movements or discomfort · None<br>**Leg discomfort** taken 1 month ago
RLS leg symptoms	Urge to move legs accompanied by unpleasant sensation;Urge to move legs worsen during rest;Urge to move legs relieved by mov<br>Urge to move legs accompanied by unpleasant sensation<br>**Urge to move legs accompanied by unpleasant sensation; Urge to move legs worsen during rest; Urge to move legs relieved by movement; Urge to move legs worse during evenings** taken 1 month ago
Current symptoms pertaining to insomnia	Afraid of not being able to sleep · Difficulty falling asleep · Difficulty staying asleep · Early awakening · Unrefreshing sleep · Other · None<br>**Difficulty falling asleep; Difficulty staying asleep** taken 1 month ago
Current symptoms pertaining to parasomnia / REM behaviors	None;<br>Bedwetting · Eating in the night · Injury to self or others during sleep · Nightmares · Night terrors<br>**None** taken 1 month ago

©2019 Epic Systems Corporation. Confidential.

Sleep Habits

Sleep Habits - Sleep Habits

Show: Last Filed | All Choices

Values By

Sleep Habits

Weekends (bedtime)	1100<br>**1100** taken 1 month ago	Weekends (waketime)	0800<br>**0800** taken 1 month ago
Weekends (hours of sleep)	8<br>**8** taken 1 month ago		
Favorite sleep position	On my side · On my back · On my abdomen<br>**On my side; On my back** taken 1 month ago		
On average how long does it take to fall asleep? (minutes)	35<br>**35** taken 1 month ago		
Number of awakenings per night	0 · 1 · 2 · 3 · 4 · 5 · 6 · 7 · 8 · 9 · 10<br>**3** taken 1 month ago		
Is length of awakening known	Yes · No<br>**Yes** taken 1 month ago		
Length of each awakening (Minimum: minutes)	5<br>**5 minutes** taken 1 month ago	Length of each awakening (Maximum: minutes)	15<br>**15 minutes** taken 1 month ago
Causes for awakenings	bed partner · light · pain/discomfort · pet · nocturia · temperature · other<br>**pain/discomfort** taken 1 month ago		

Naps

Daytime nap	Yes · No

©2019 Epic Systems Corporation. Confidential.

Sleep Meds History

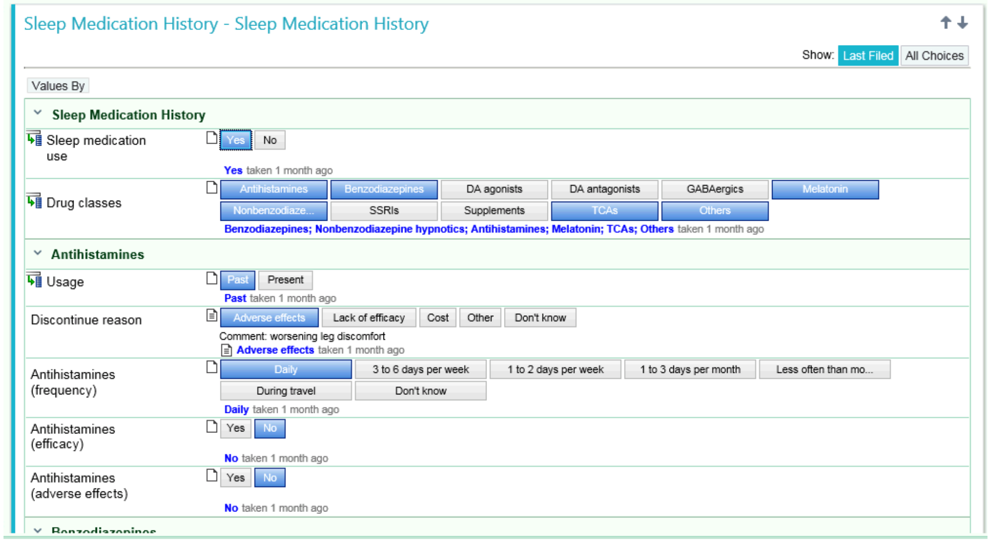

Sleep Medication History - Sleep Medication History

Show: Last Filed | All Choices

Values By

Sleep Medication History

Sleep medication use: Yes | No
Yes taken 1 month ago

Drug classes: Antihistamines | Benzodiazepines | DA agonists | DA antagonists | GABAergics | Melatonin | Nonbenzodiaze... | SSRIs | Supplements | TCAs | Others
Benzodiazepines; Nonbenzodiazepine hypnotics; Antihistamines; Melatonin; TCAs; Others taken 1 month ago

Antihistamines

Usage: Past | Present
Past taken 1 month ago

Discontinue reason: Adverse effects | Lack of efficacy | Cost | Other | Don't know
Comment: worsening leg discomfort
Adverse effects taken 1 month ago

Antihistamines (frequency): Daily | 3 to 6 days per week | 1 to 2 days per week | 1 to 3 days per month | Less often than mo... | During travel | Don't know
Daily taken 1 month ago

Antihistamines (efficacy): Yes | No
No taken 1 month ago

Antihistamines (adverse effects): Yes | No
No taken 1 month ago

Benzodiazepines

©2019 Epic Systems Corporation. Confidential.

Impressions

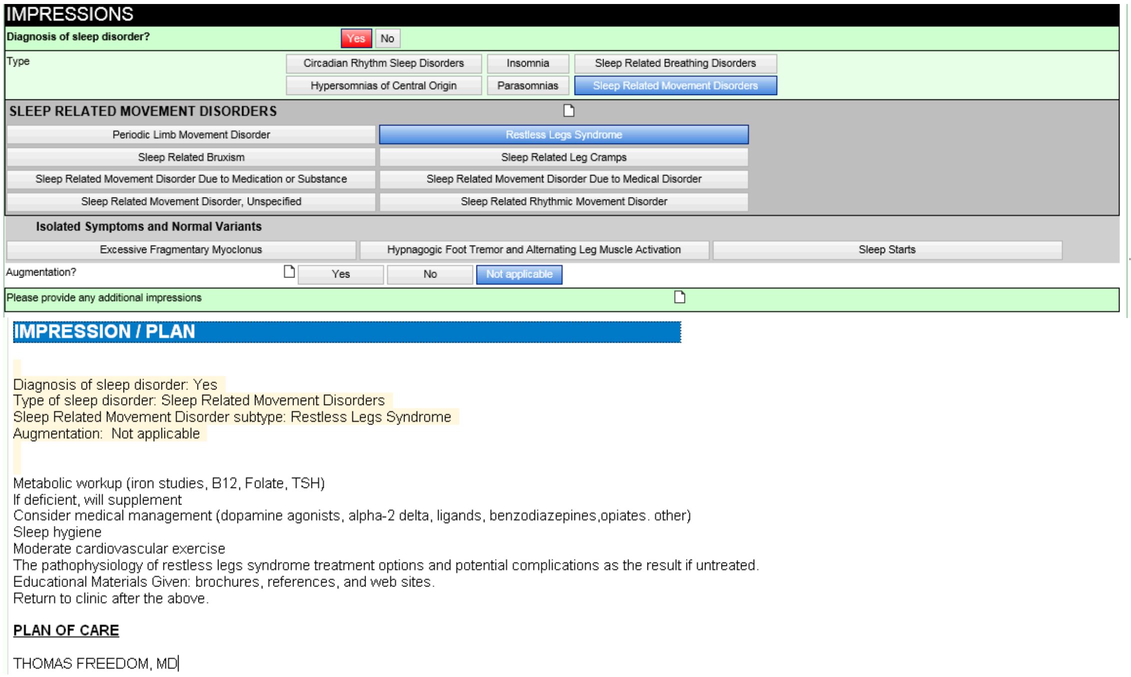

IMPRESSIONS

Diagnosis of sleep disorder? Yes | No

Type: Circadian Rhythm Sleep Disorders | Insomnia | Sleep Related Breathing Disorders | Hypersomnias of Central Origin | Parasomnias | Sleep Related Movement Disorders

SLEEP RELATED MOVEMENT DISORDERS

Periodic Limb Movement Disorder | Restless Legs Syndrome | Sleep Related Bruxism | Sleep Related Leg Cramps | Sleep Related Movement Disorder Due to Medication or Substance | Sleep Related Movement Disorder Due to Medical Disorder | Sleep Related Movement Disorder, Unspecified | Sleep Related Rhythmic Movement Disorder

Isolated Symptoms and Normal Variants

Excessive Fragmentary Myoclonus | Hypnagogic Foot Tremor and Alternating Leg Muscle Activation | Sleep Starts

Augmentation? Yes | No | Not applicable

Please provide any additional impressions

IMPRESSION / PLAN

Diagnosis of sleep disorder: Yes
Type of sleep disorder: Sleep Related Movement Disorders
Sleep Related Movement Disorder subtype: Restless Legs Syndrome
Augmentation: Not applicable

Metabolic workup (iron studies, B12, Folate, TSH)
If deficient, will supplement
Consider medical management (dopamine agonists, alpha-2 delta, ligands, benzodiazepines,opiates. other)
Sleep hygiene
Moderate cardiovascular exercise
The pathophysiology of restless legs syndrome treatment options and potential complications as the result if untreated.
Educational Materials Given: brochures, references, and web sites.
Return to clinic after the above.

PLAN OF CARE

THOMAS FREEDOM, MD

©2019 Epic Systems Corporation. Confidential.
