# Supplementary material for: Quality improvement and practice-based research in sleep medicine using structured clinical documentation in the electronic medical record
Source: Sleep Sci Pract. Author manuscript; Available in PMC 2020 May 11. (PMC7213673; doi:10.1186/s41606-019-0038-2)
Supplement: Additional file 2 — Complete descriptive report of patients at initial visit. [file NIHMS1574724-supplement-Additional_file_2.pdf]

Sleep Disorders

Descriptive Report

2019-02-01

- Gender
- Race
- BMI
- Years of Education
- Tobacco User
- Alcohol User
- Age at encounter
- Age Sleep Problem Began
- Sleep Problem Duration
- Sleep Study Type
- Sleep Disorder Diagnosis
- CESD Interpretation
- GAD7 Interpretation
- ESS Interpretation
- ISI Interpretation
- PSQI Interpretation
- RLS Interpretation
- Current Symptoms Sleep Disruptors
- Current Symptoms Breathing
- Current Symptoms Limb
- Current Symptoms RLS
- Current Symptoms Insomnia
- Current Symptoms REM
- Current Symptoms Awake
- Current Symptoms Narcolepsy
- Sleep Medication Use
- Family History of Circardian Rhythm Disorder
- Family Members with History of Circardian Rhythm Disorder
- Family History of Hypersomnia
- Family Members with History of Hypersomnia
- Family History of Insomnia
- Family Members with History of Insomnia
- Family History of Narcolepsy
- Family Members with History of Narcolepsy
- Family History of Parasomnia
- Family Members with History of Parasomnia
- Family History of Parkinsons
- Family Members with History of Parkinsons
- Family History of REM Sleep Disorder
- Family Members with History of REM Sleep Disorder
- Family History of RLS
- Family Members with History of RLS
- Family History of Sleep Apnea

- Family Members with History of Sleep Apnea
- Family History of Sleep Movement Disorder
- Family Members with History of Sleep Movement Disorder
- Family History of Snoring
- Family Members with History of Snoring

Total Patients = 1157

All DodoNA eligible patients regardless of enrollment

Encounters restricted to the following visit types: Initial Visit

---

Gender

Gender	Count	Percent
Female	729	63
Male	428	37

Race

Value	Percent	Count	Male	Female
Caucasian	59.6	689	253	436
NA	29	336	129	207
African American	4.9	57	14	43
Hispanic/Latino	3.5	40	15	25
Asian	2.9	33	15	18
American Indian or Alaska Native	0.1	1	1	0
Other	0.1	1	1	0

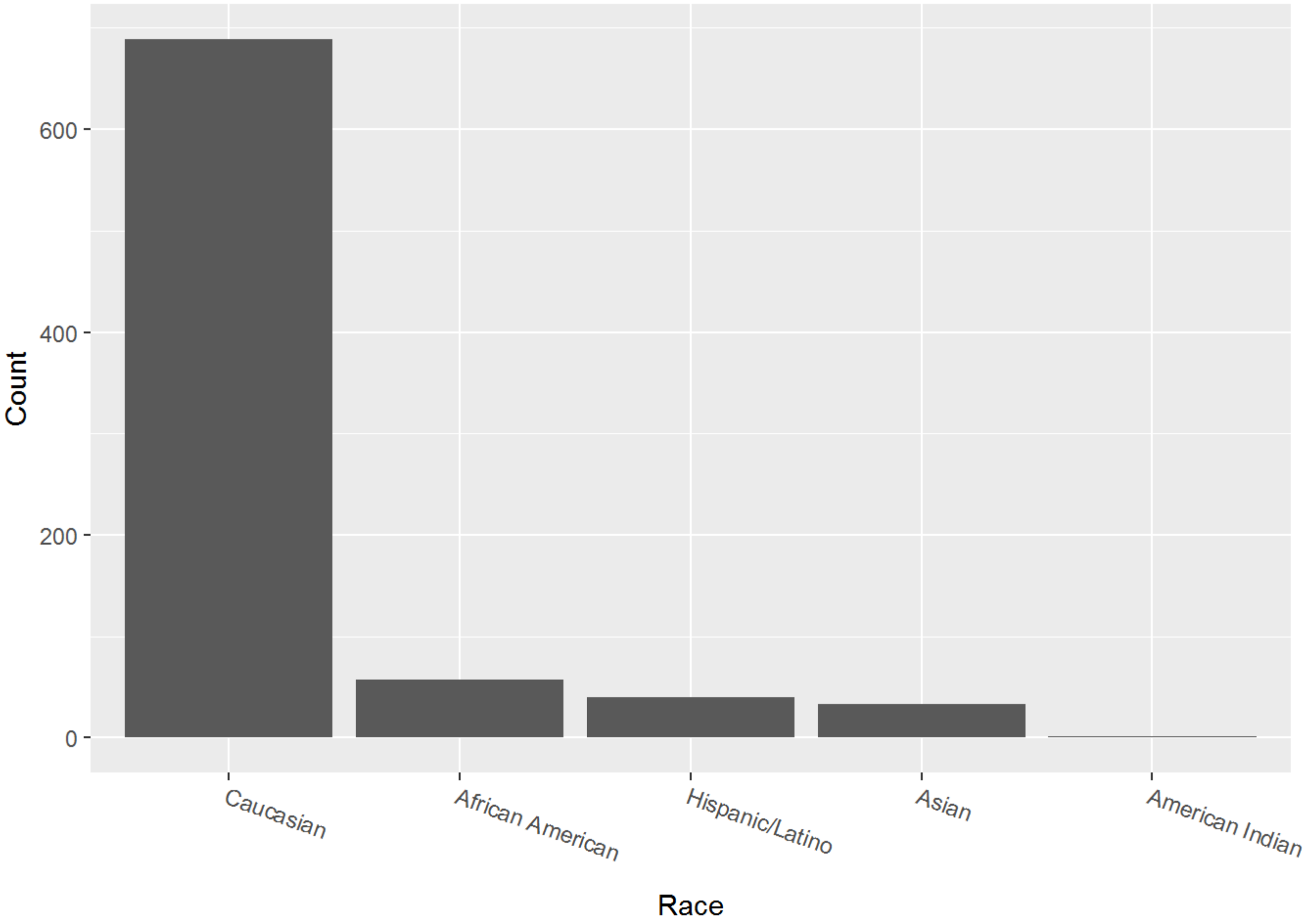

BMI

Min	Median	Mean	Max	St.Dev	No.Data
16	30	31.27	79	8.02	30

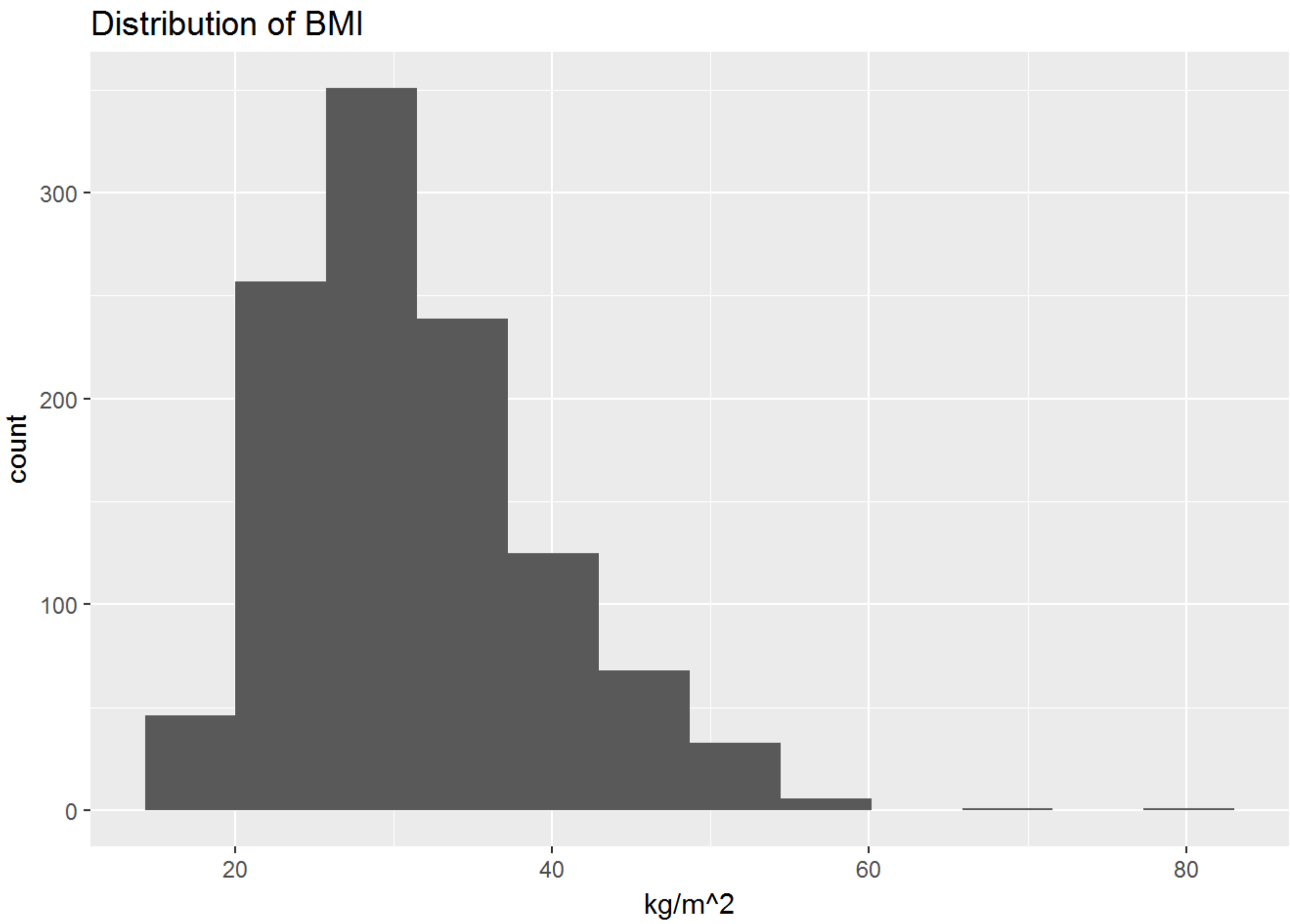
Distribution of BMI
count
300
200
100
0
20
40
60
80
kg/m^2

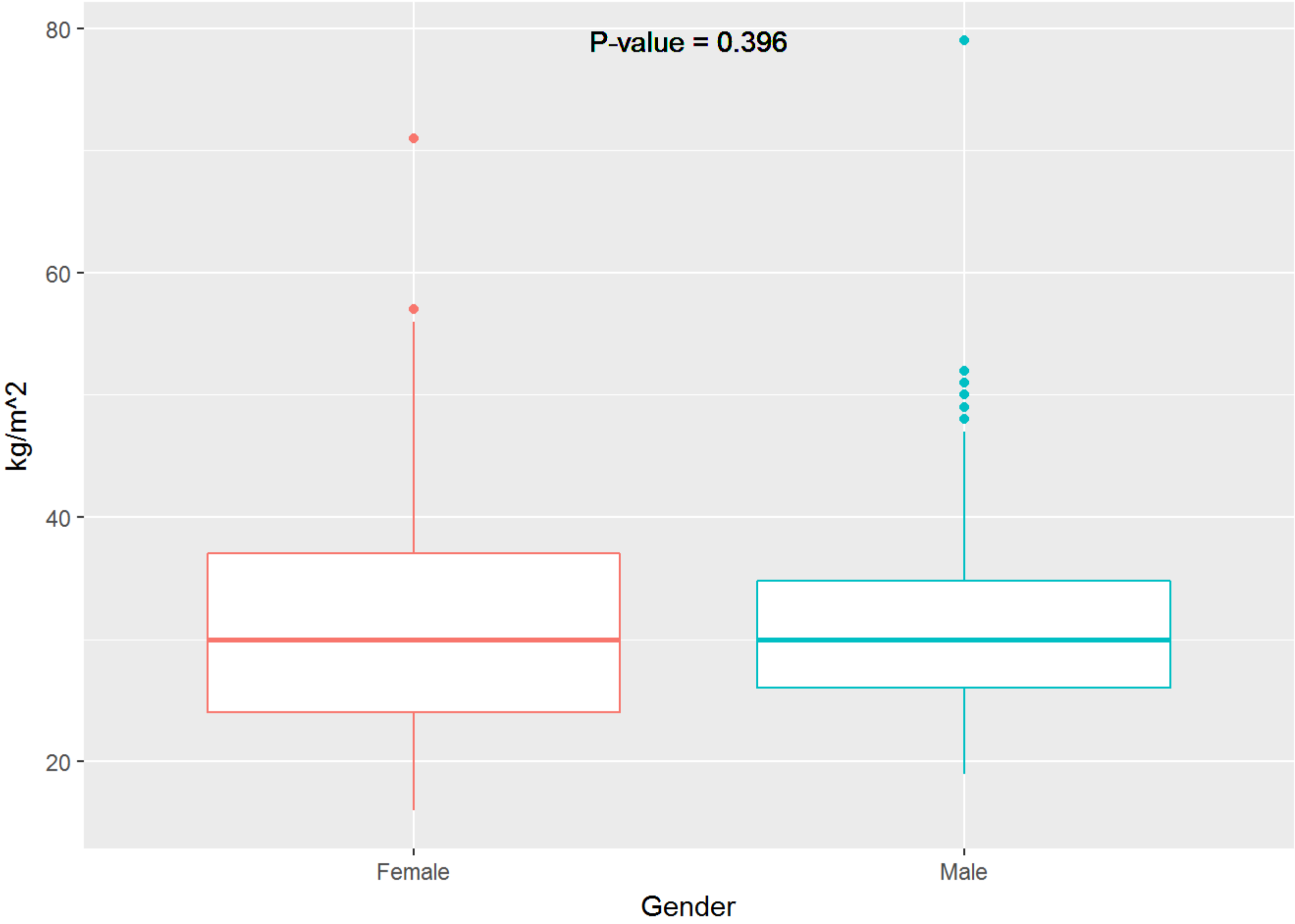
P-value = 0.396
80
60
40
20
kg/m^2
Female
Male
Gender

Years of Education

Min	Median	Mean	Max	St.Dev	No.Data
3	16	15.98	30	3.083	80

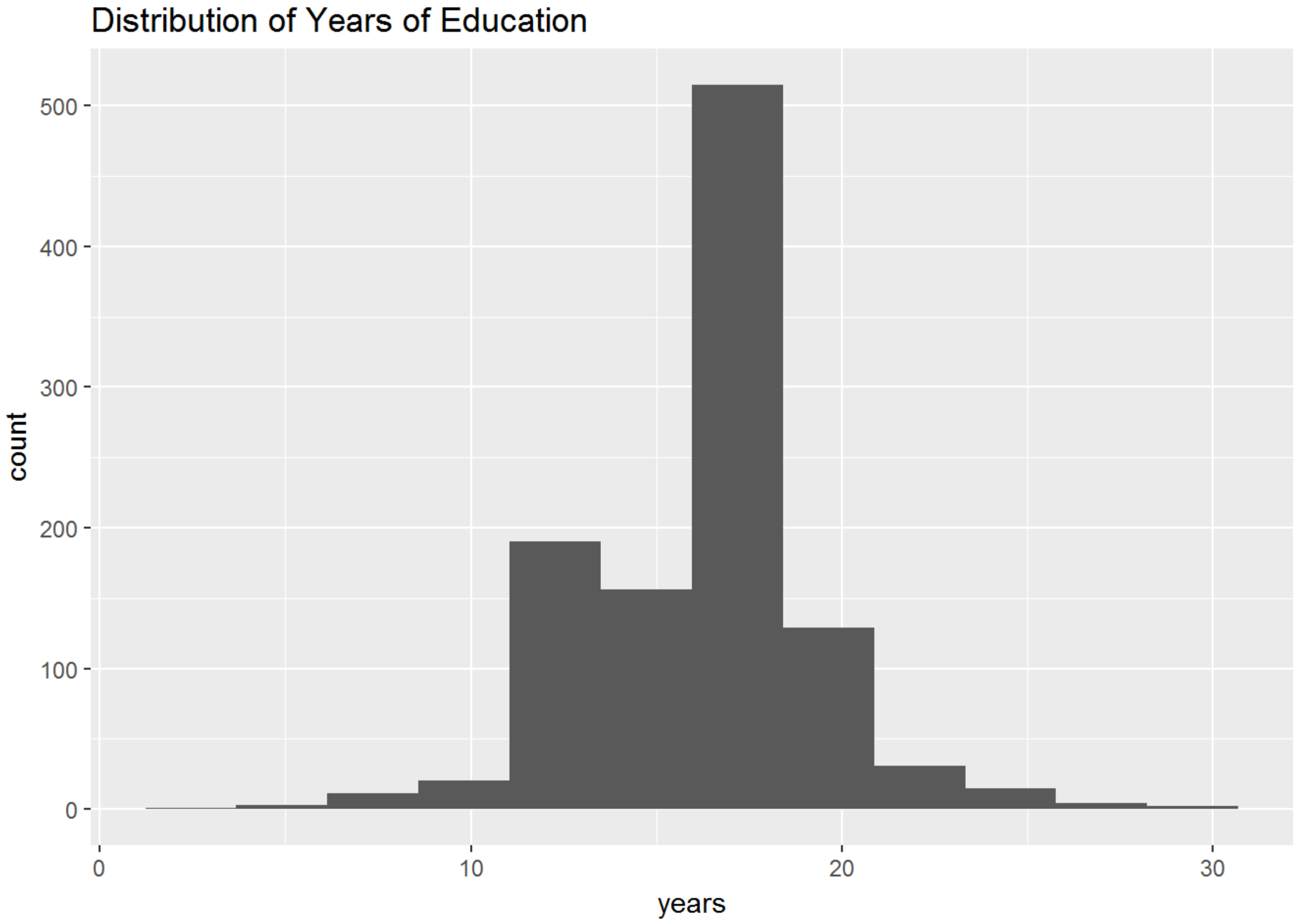

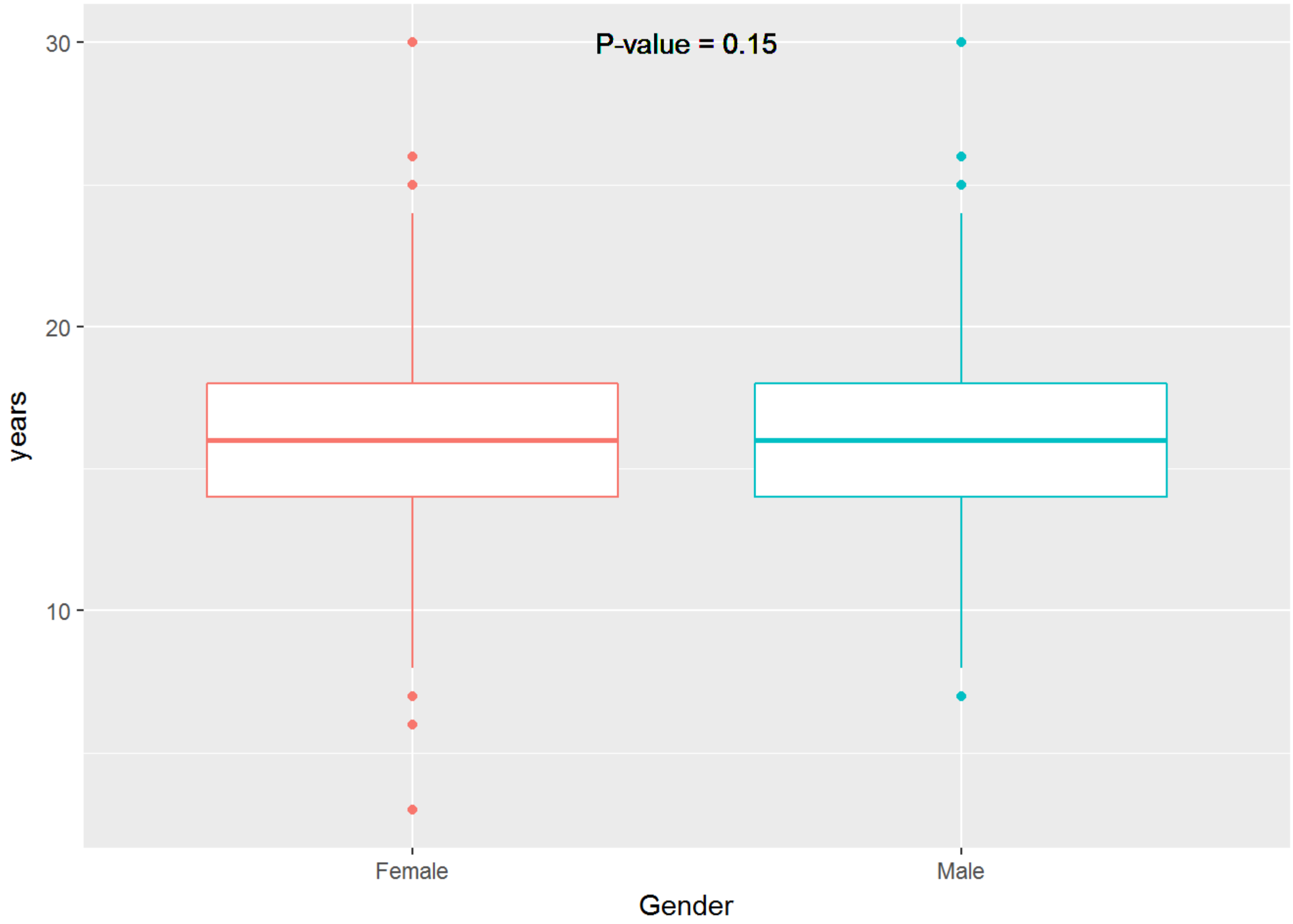

Tobacco User

Value	Percent	Count	Male	Female
Never	57.2	662	227	435
Quit	33.8	391	154	237
Yes	6.3	73	34	39
NA	2.1	24	10	14
Passive	0.4	5	2	3
Not Asked	0.2	2	1	1

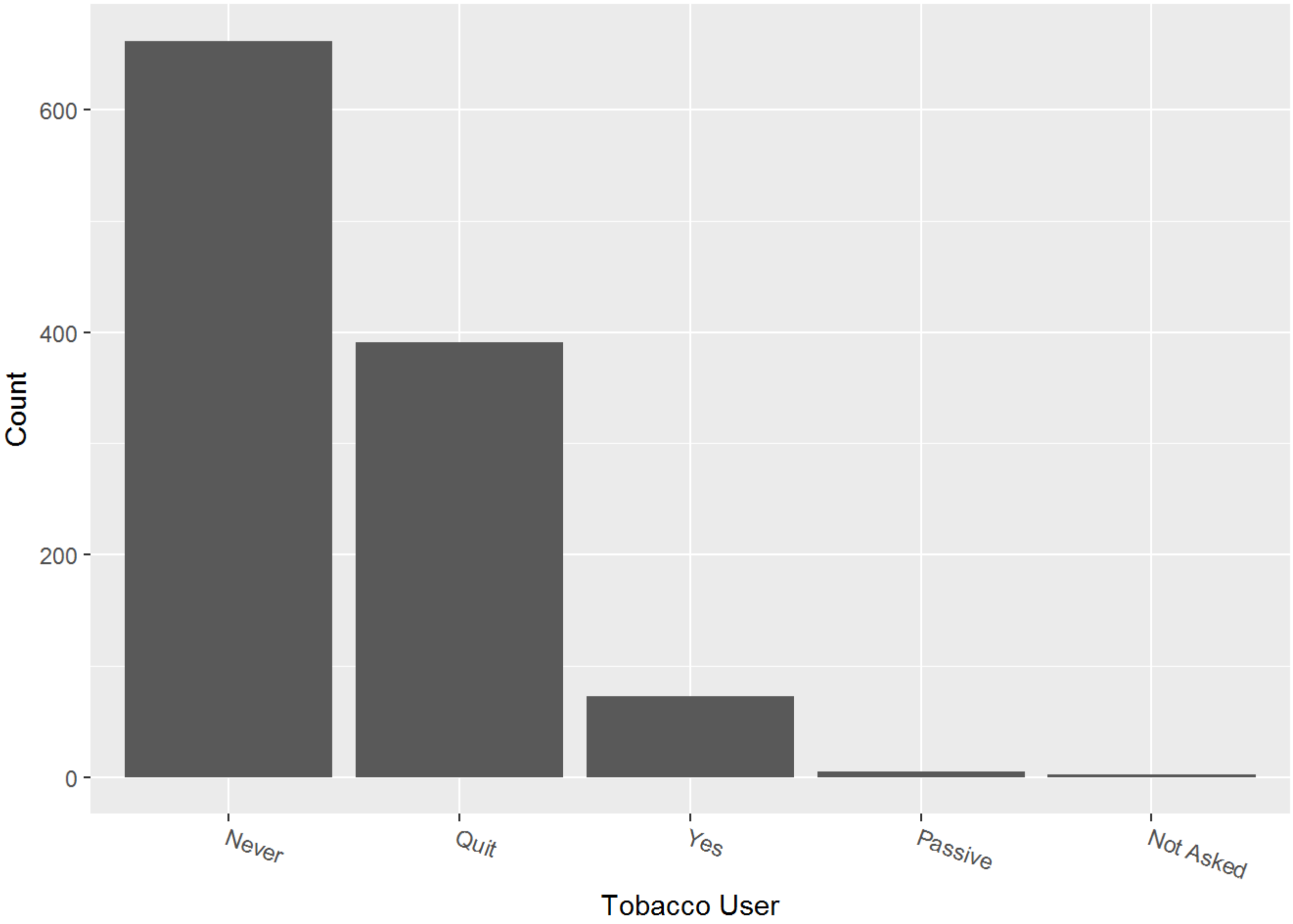

Alcohol User

Value	Percent	Count	Male	Female
Yes	54.1	626	236	390
No	43	498	177	321
NA	2.4	28	11	17
Not Asked	0.4	5	4	1

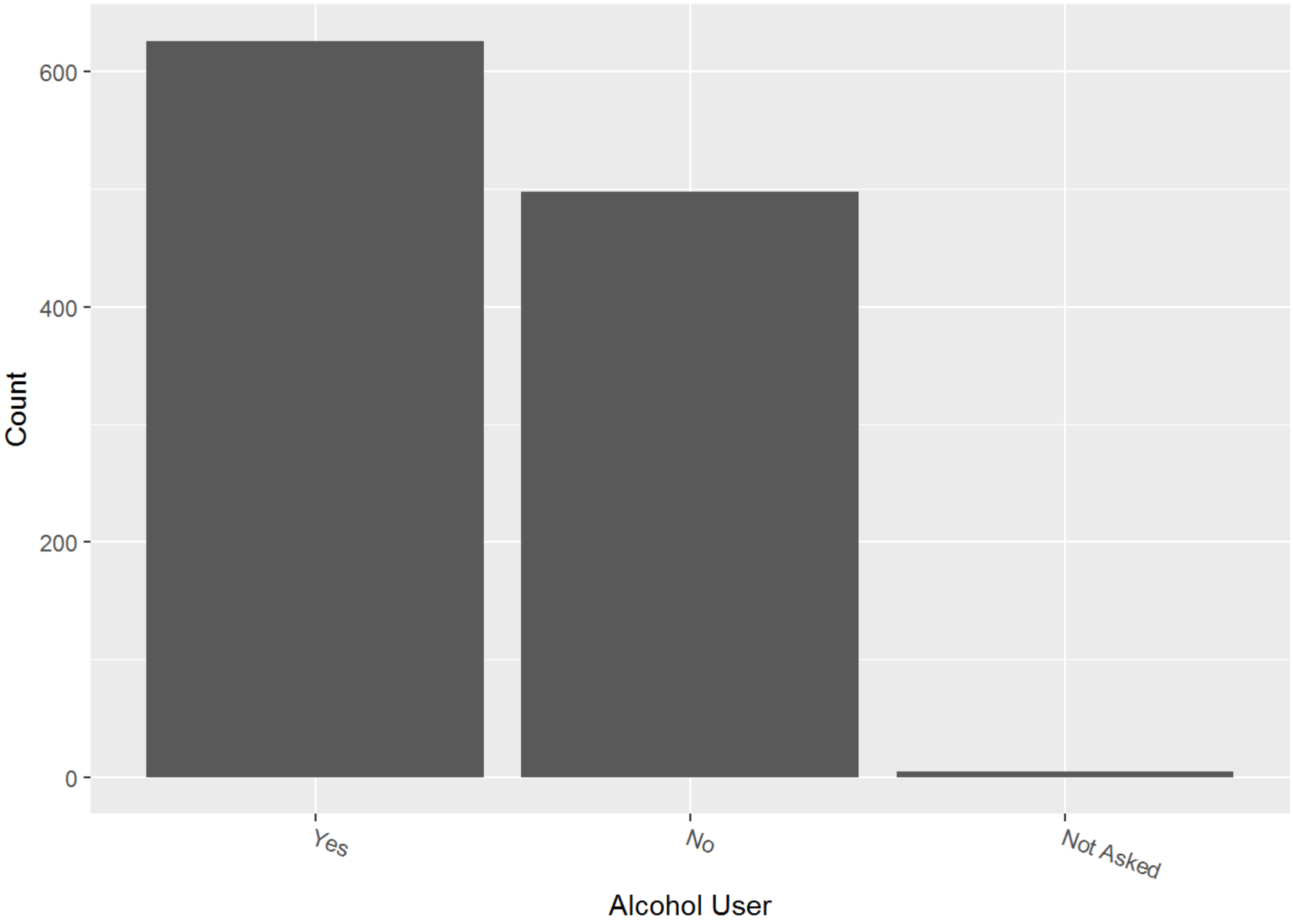

Age at encounter

Min	Median	Mean	Max	St.Dev	No.Data
18.03	56.87	55.07	93.12	15.9	0

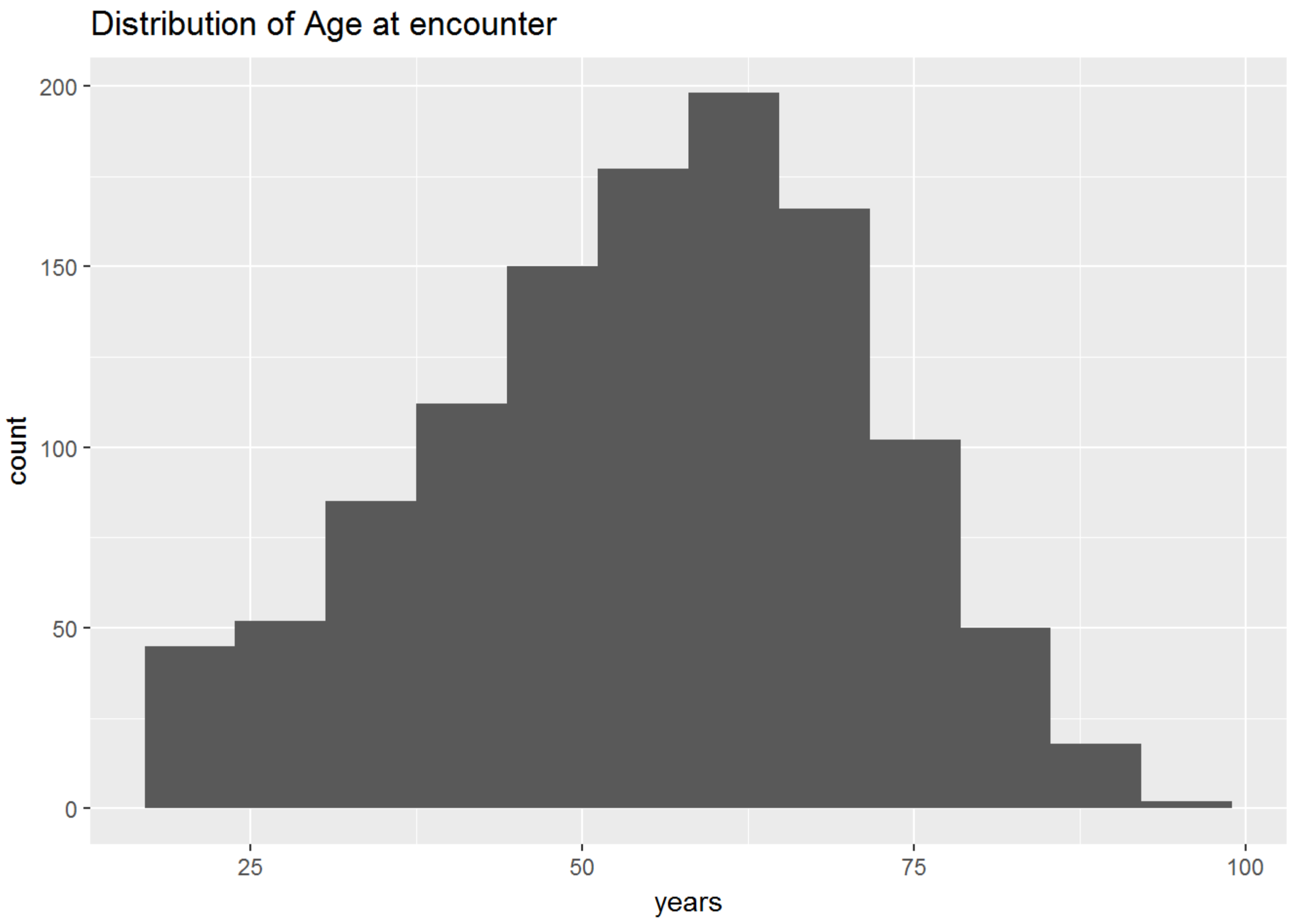
Distribution of Age at encounter
count
200
150
100
50
0
25
50
75
100
years

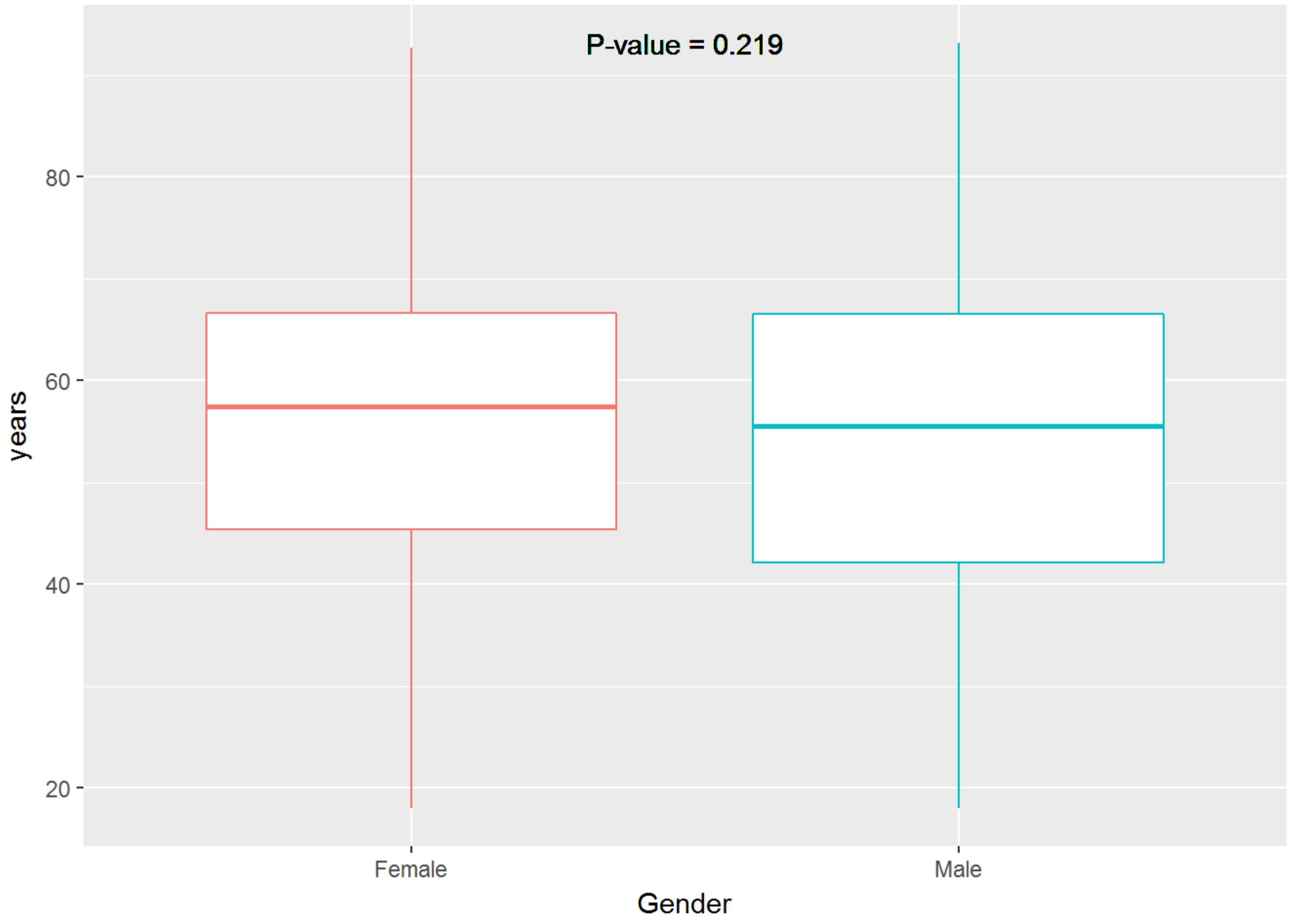
P-value = 0.219
years
80
60
40
20
Female
Male
Gender

Age Sleep Problem Began

Min	Median	Mean	Max	St.Dev	No.Data
0	43.35	42.19	87.16	17.27	620

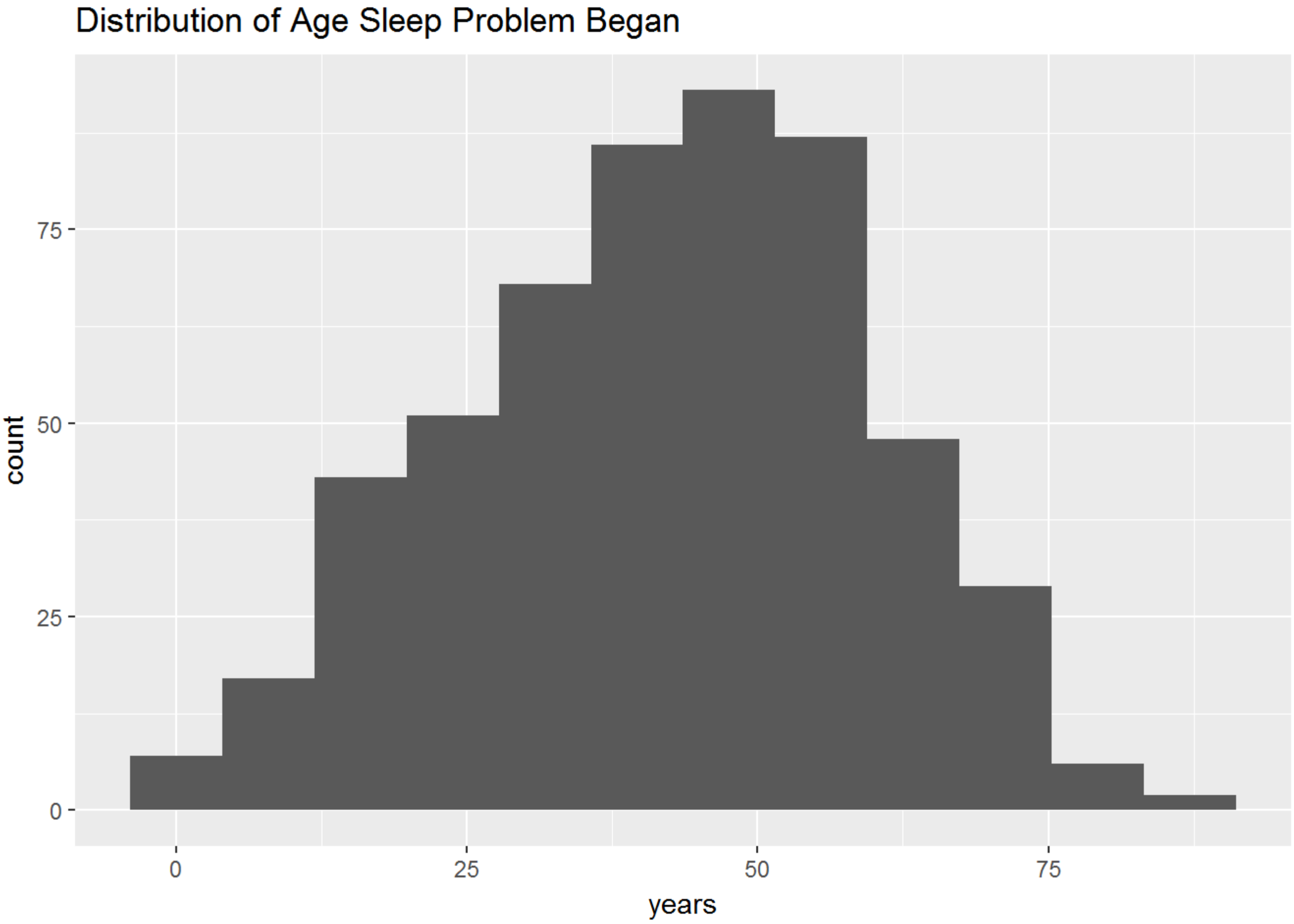

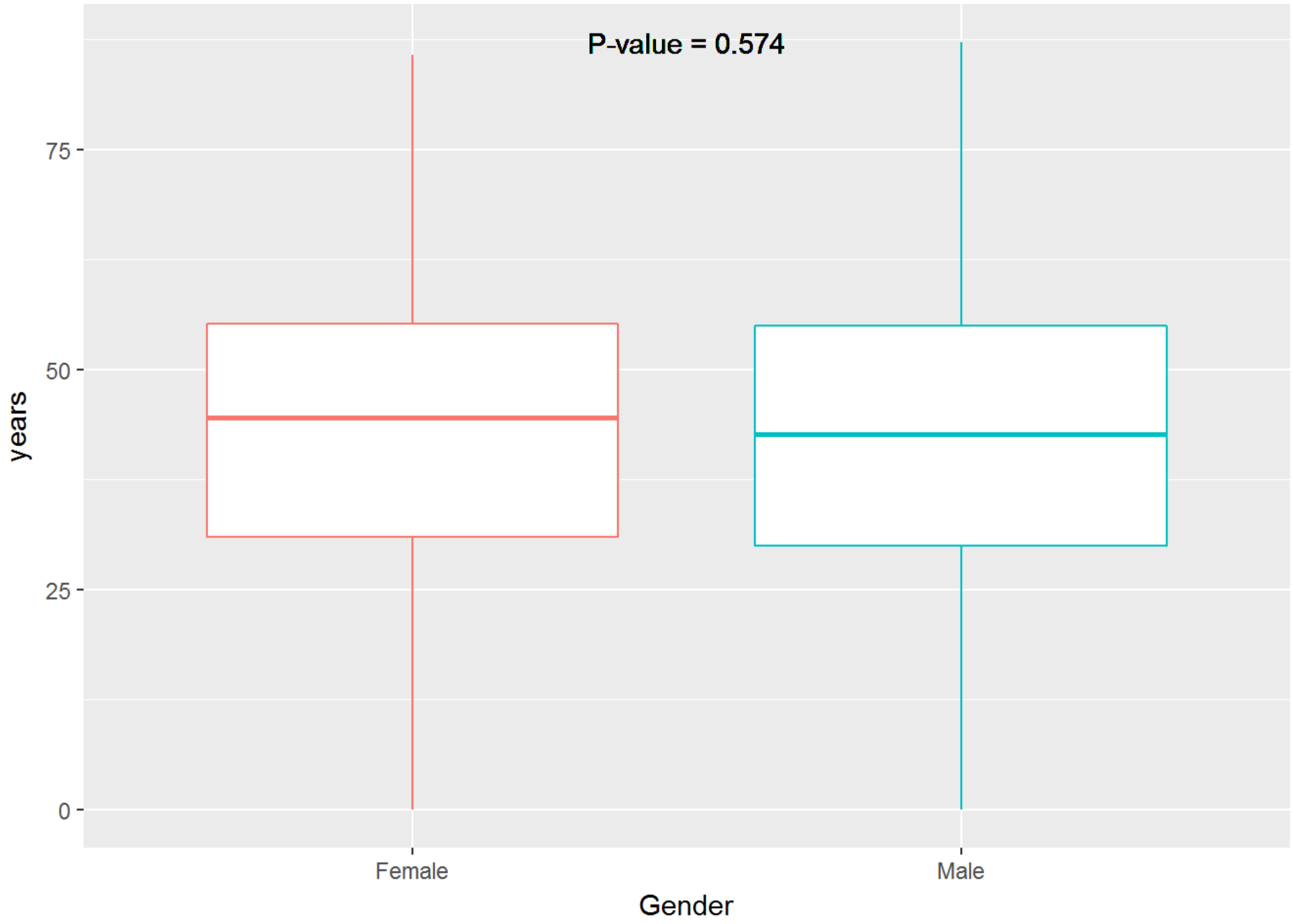

Sleep Problem Duration

Min	Median	Mean	Max	St.Dev	No.Data
0	8.572	12.1	82.74	12.19	620

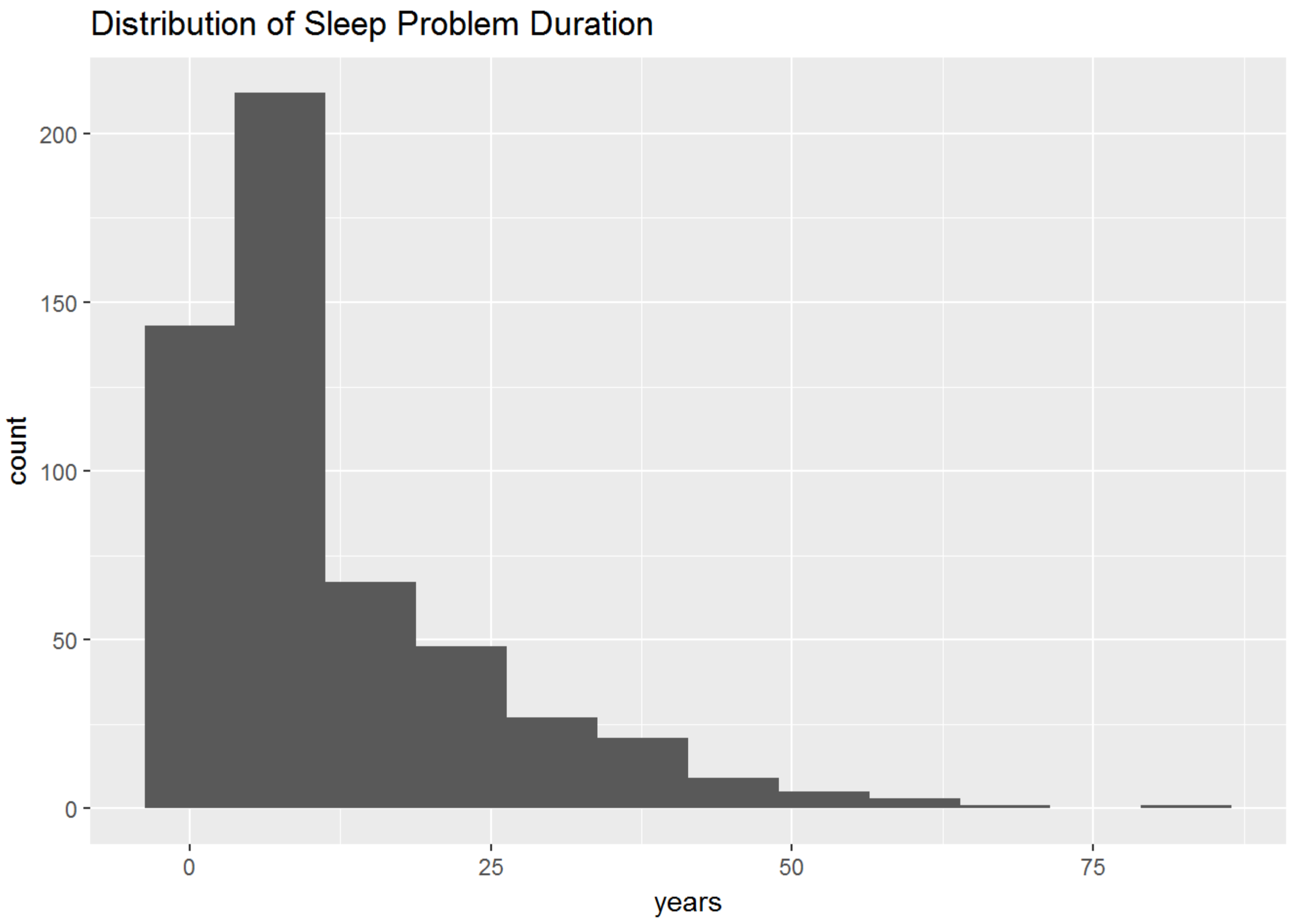
Distribution of Sleep Problem Duration
count
200
150
100
50
0
0
25
50
75
years

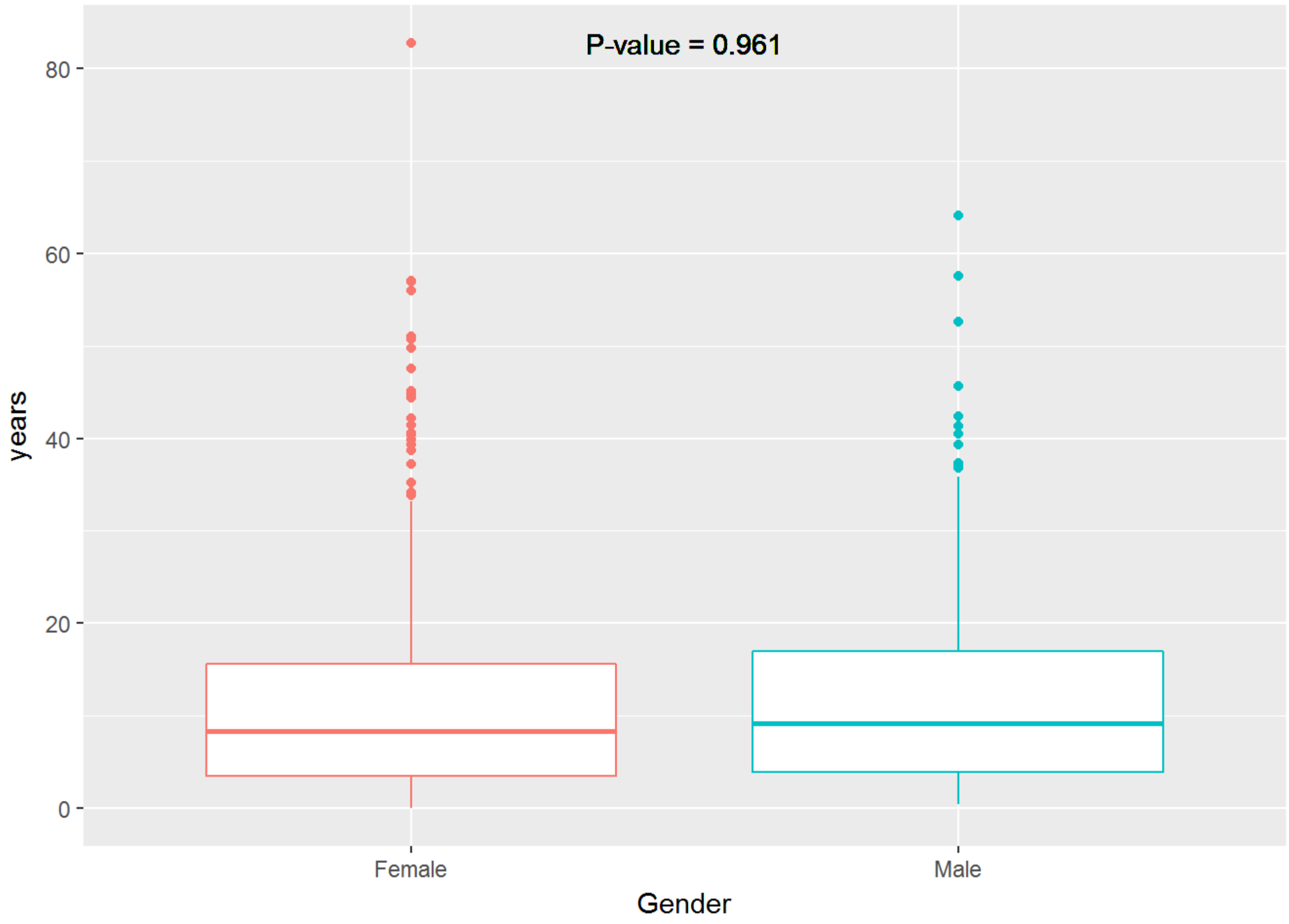
P-value = 0.961
years
80
60
40
20
0
Female
Male
Gender

Sleep Study Type

Value	Percent	Count	Male	Female
Nocturnal Polysomnography	11.2	130	42	88
PAP Titration Study	5.6	65	33	32
Split Night Study	4.5	52	21	31
Home Sleep Test	1.2	14	8	6
Don’t know	1	12	7	5
Multiple Sleep Latency Test	0.2	2	2	0
Oral Titration Study	0.1	1	1	0

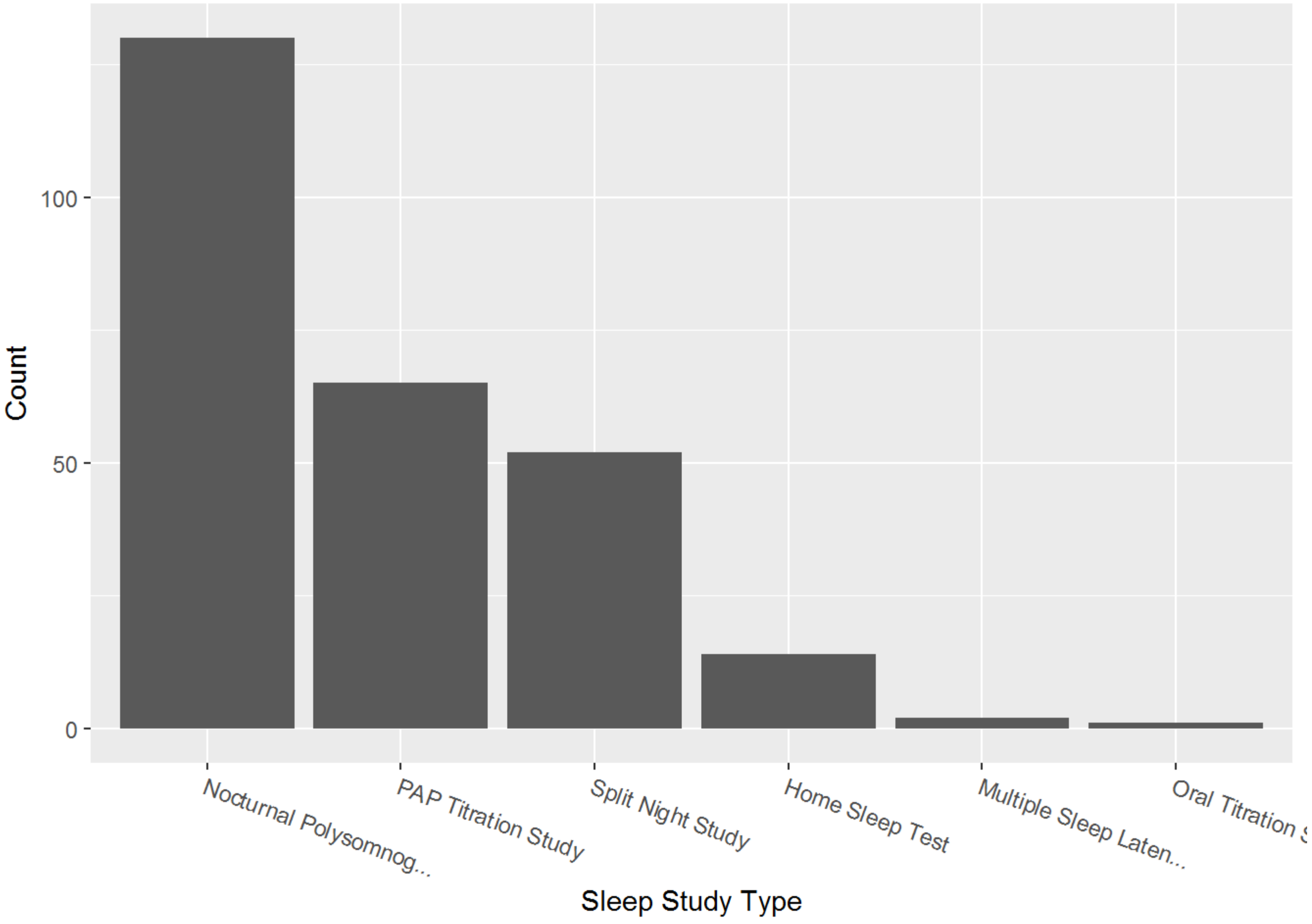

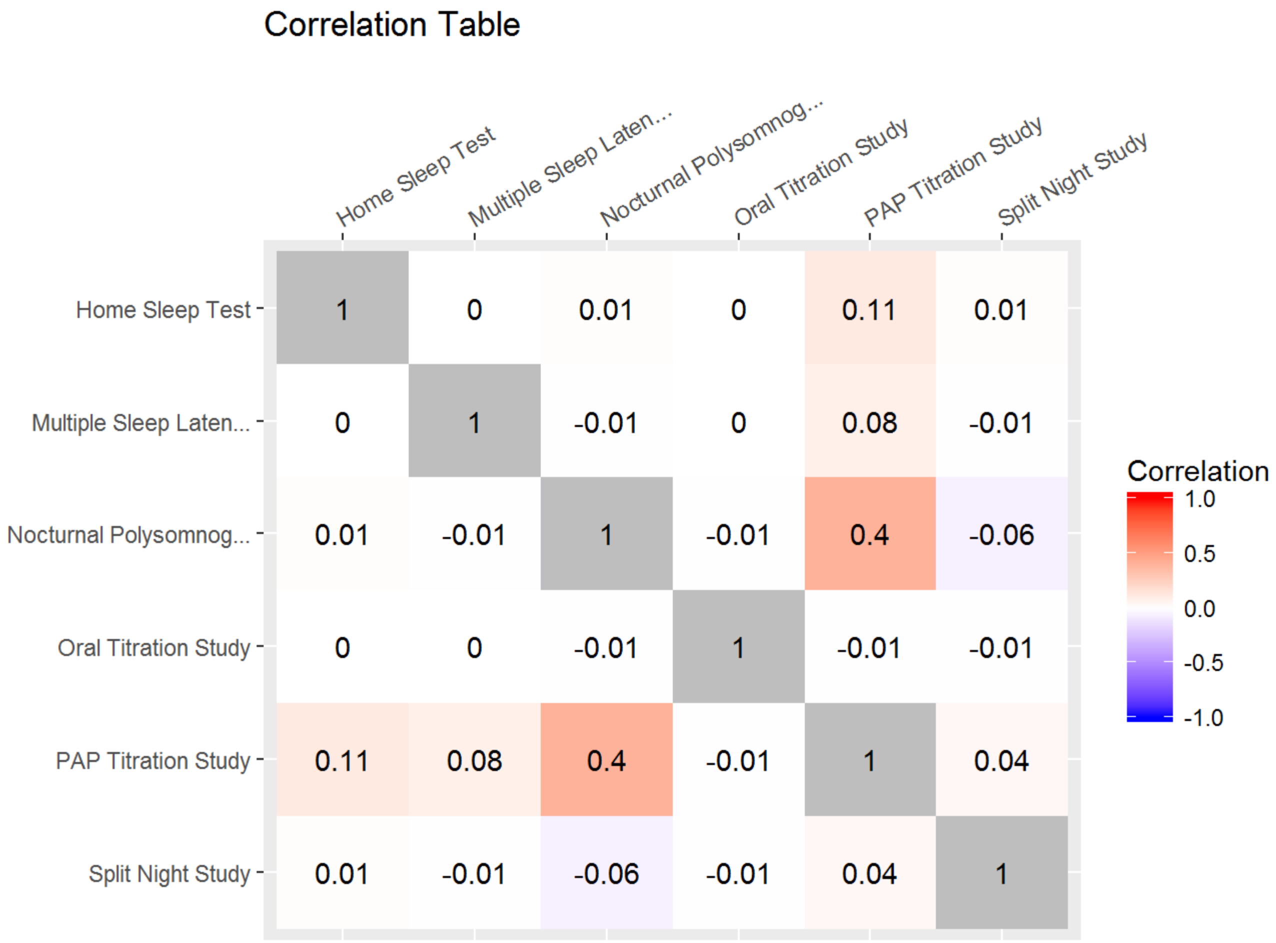

Sleep Disorder Diagnosis

Value	Percent	Count	Male	Female
1	99.2	1148	423	725
NA	0.8	9	5	4

CESD Interpretation

Value	Count	Male	Female
No Indication of Depression	680	260	420
Mild to Moderate Depression	172	63	109
Possibility of Major Depression	211	74	137
NA	94	31	63

Min	Median	Mean	Max	St.Dev	No.Data
0	12	14.24	55	10.54	94

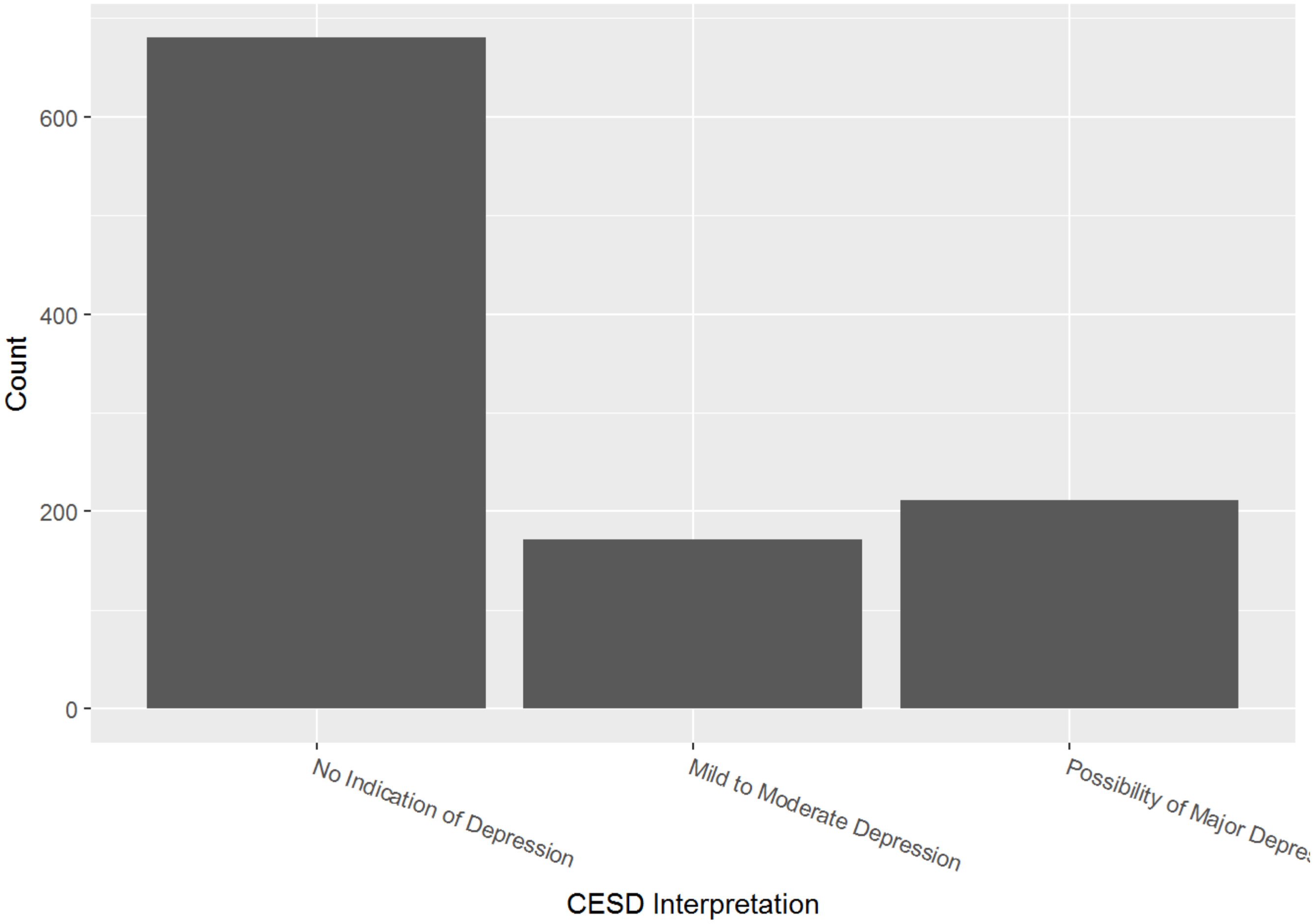
Count
600
400
200
0
No Indication of Depression
Mild to Moderate Depression
CESD Interpretation

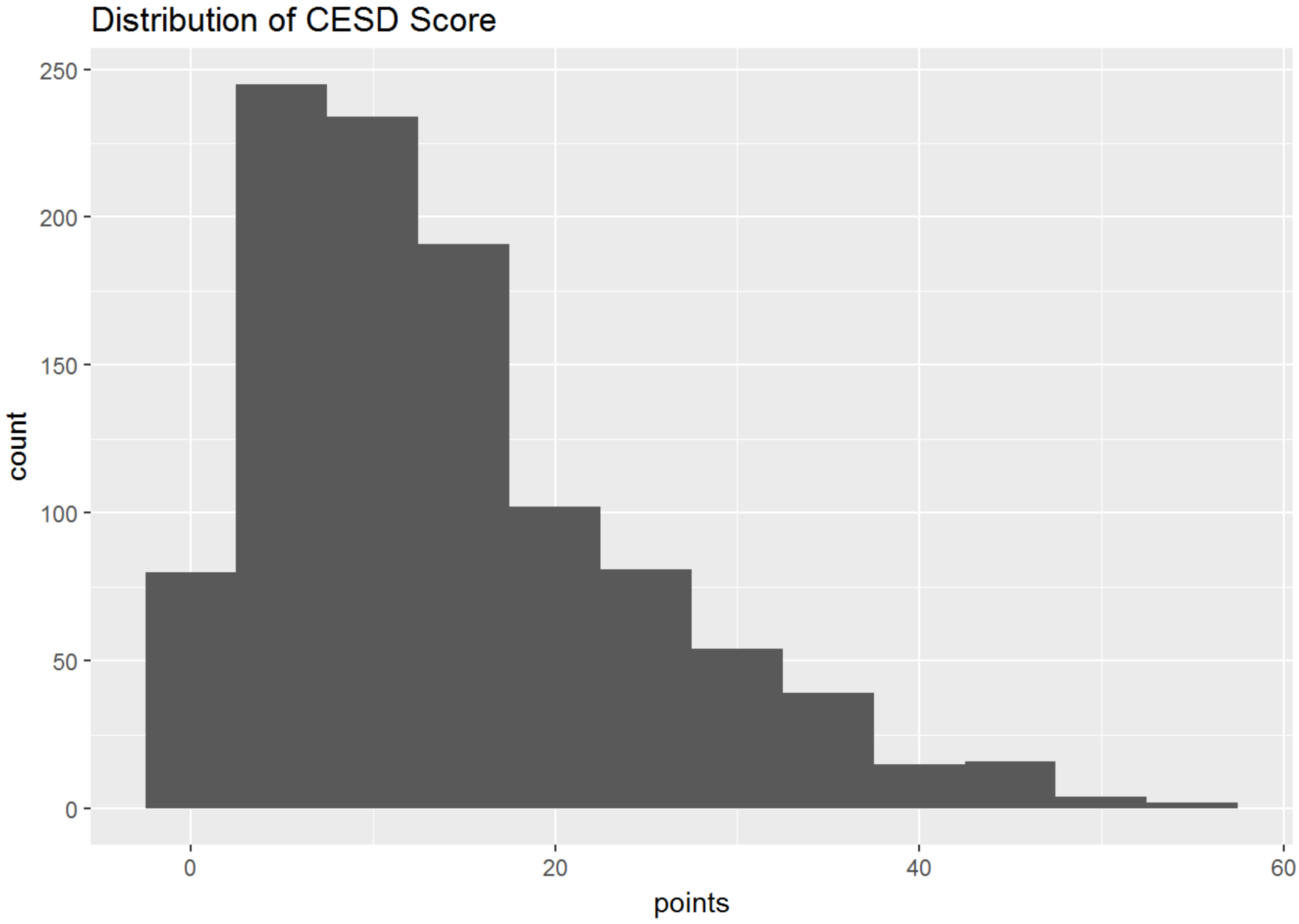
Distribution of CESD Score
count
250
200
150
100
50
0
0
20
40
60
points

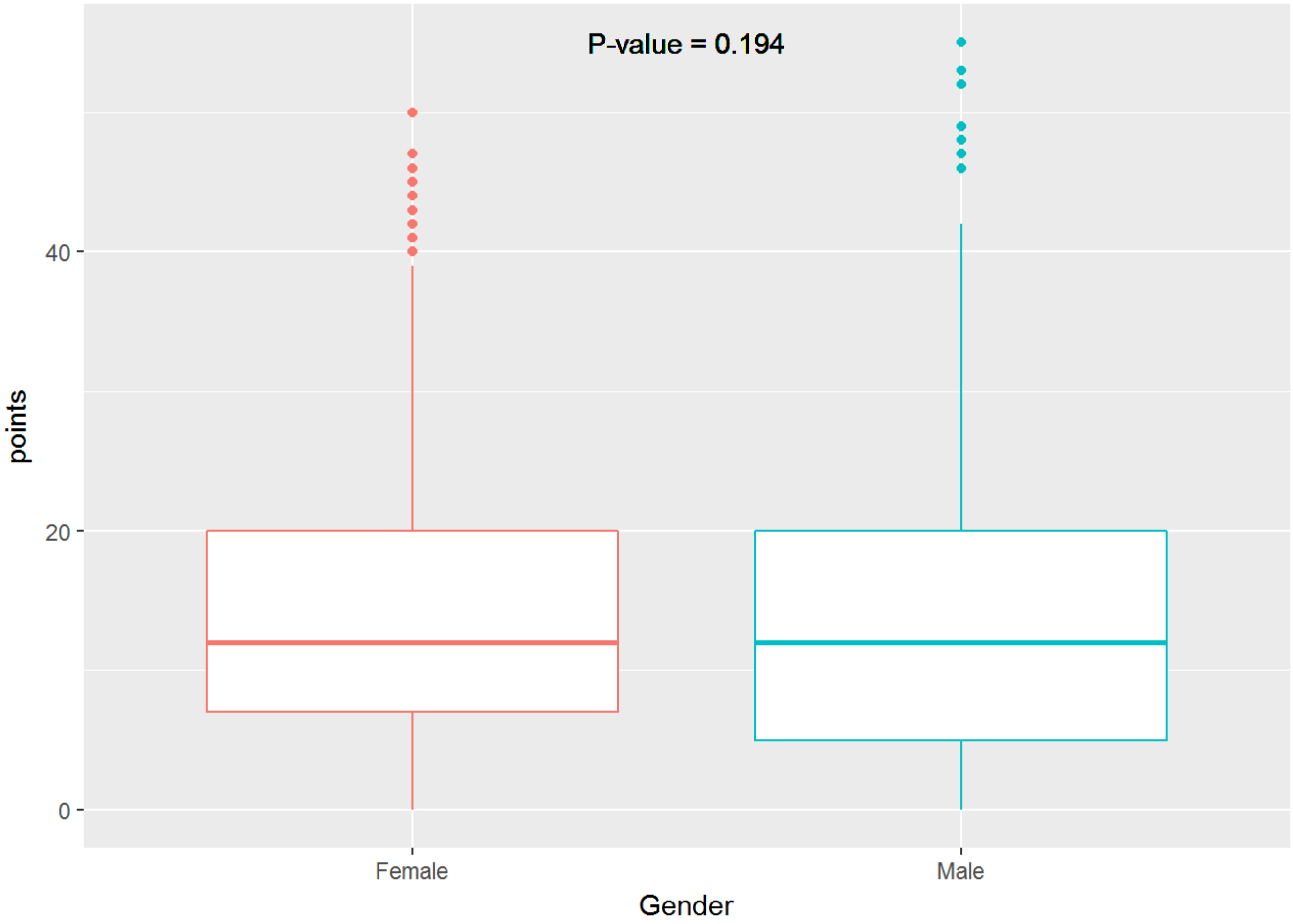

GAD7 Interpretation

Value	Count	Male	Female
Normal	531	218	313
Mild Anxiety	291	92	199
Moderate Anxiety	143	52	91
Severe Anxiety	112	37	75
NA	80	29	51

Min	Median	Mean	Max	St.Dev	No.Data
0	5	6.028	21	5.505	80

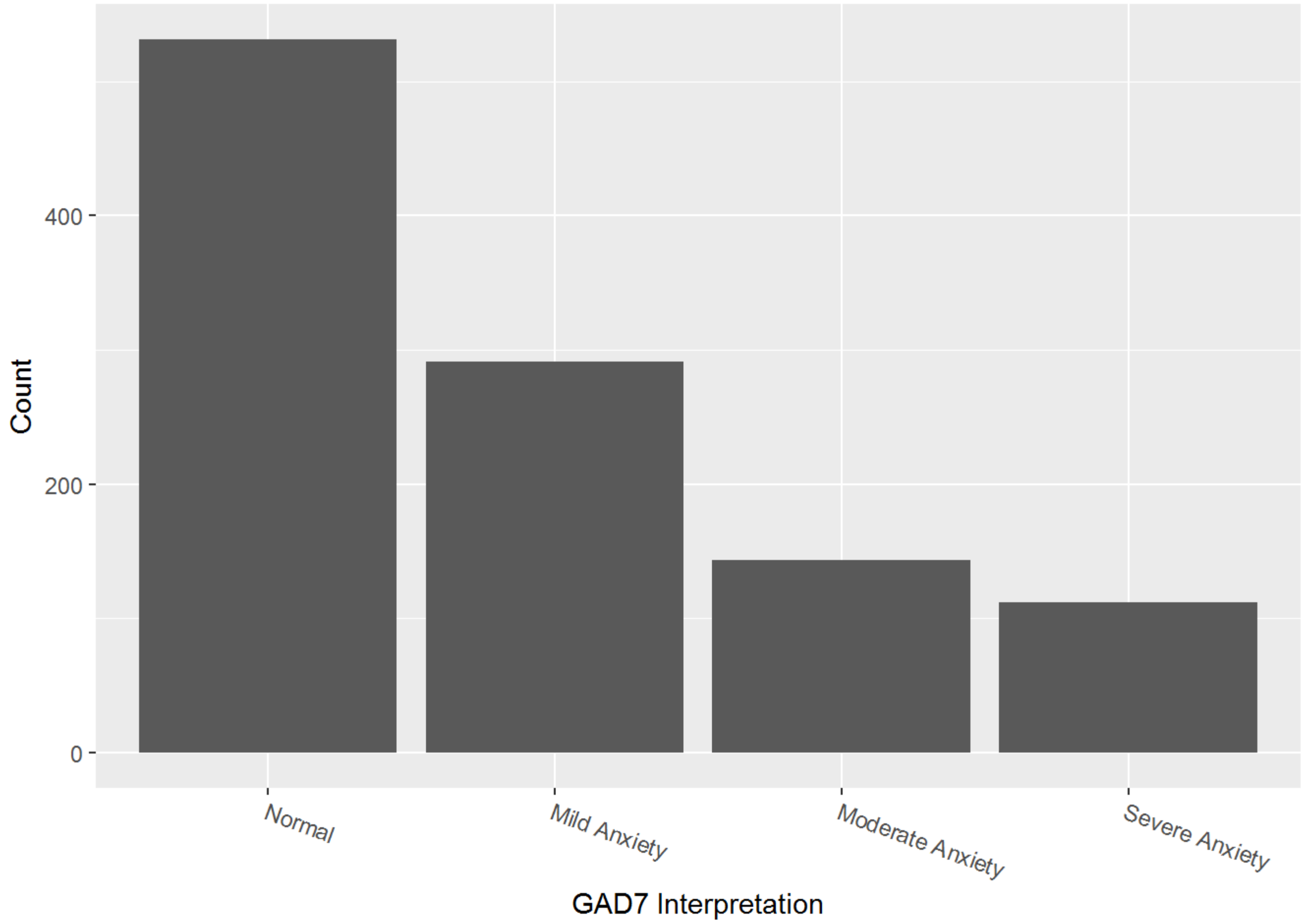
Count
400
200
0
Normal
Mild Anxiety
Moderate Anxiety
Severe Anxiety
GAD7 Interpretation

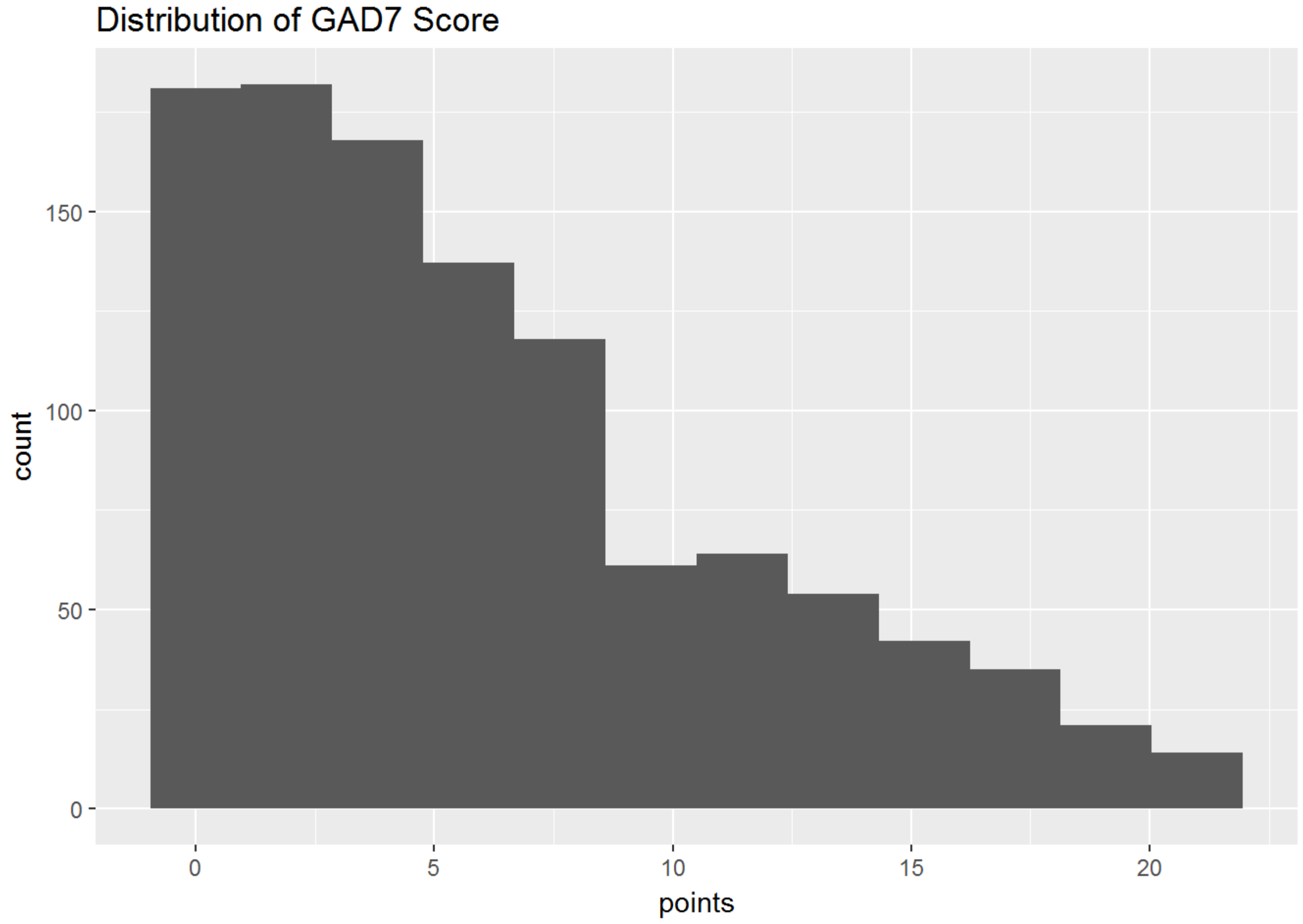
Distribution of GAD7 Score
count
150
100
50
0
0
5
10
15
20
points

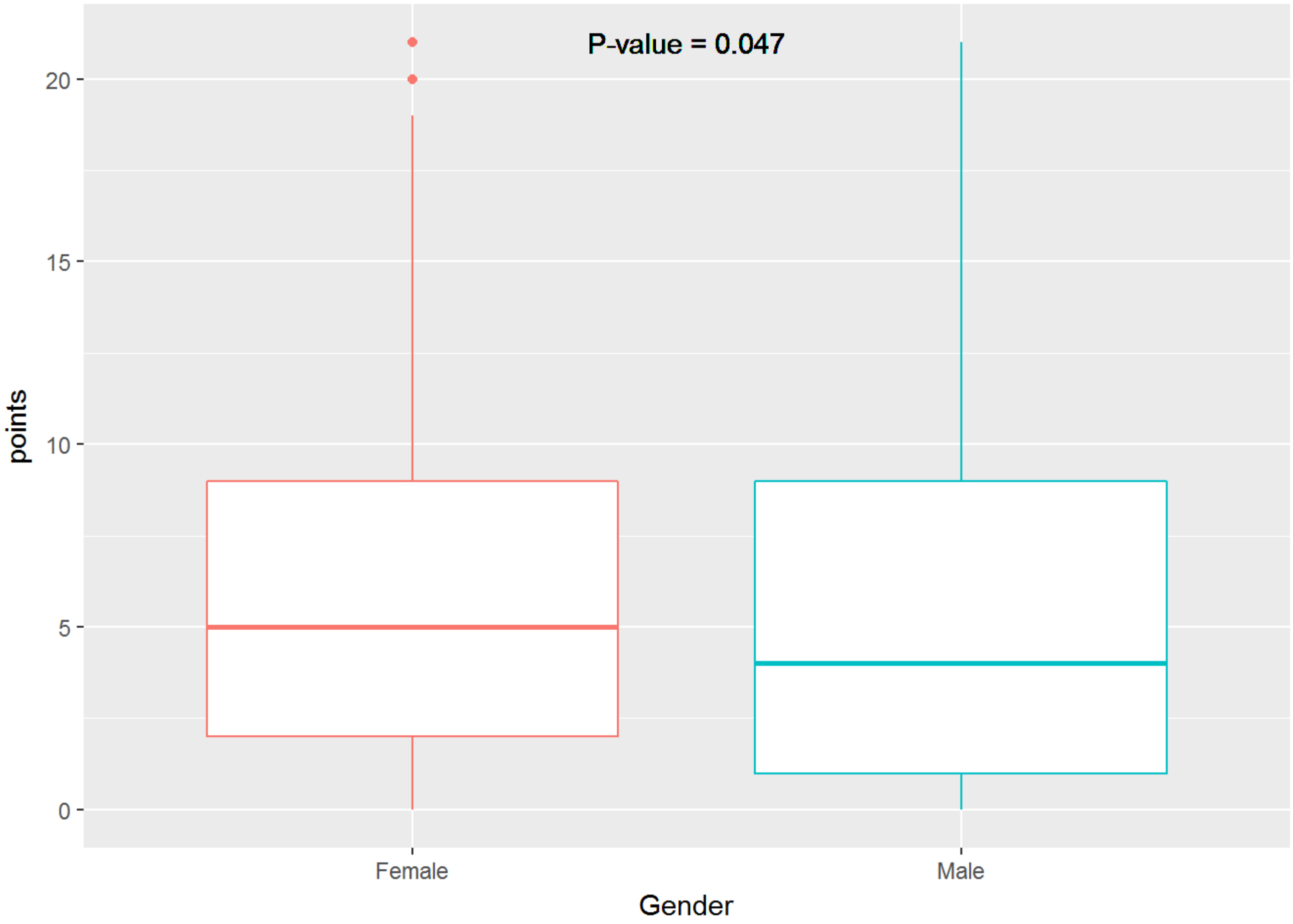

ESS Interpretation

Value	Count	Male	Female
Not Sleepy	693	258	435
Sleepy	335	120	215
Very Sleepy	69	28	41
NA	60	22	38

Min	Median	Mean	Max	St.Dev	No.Data
0	7	8.245	24	5.399	60

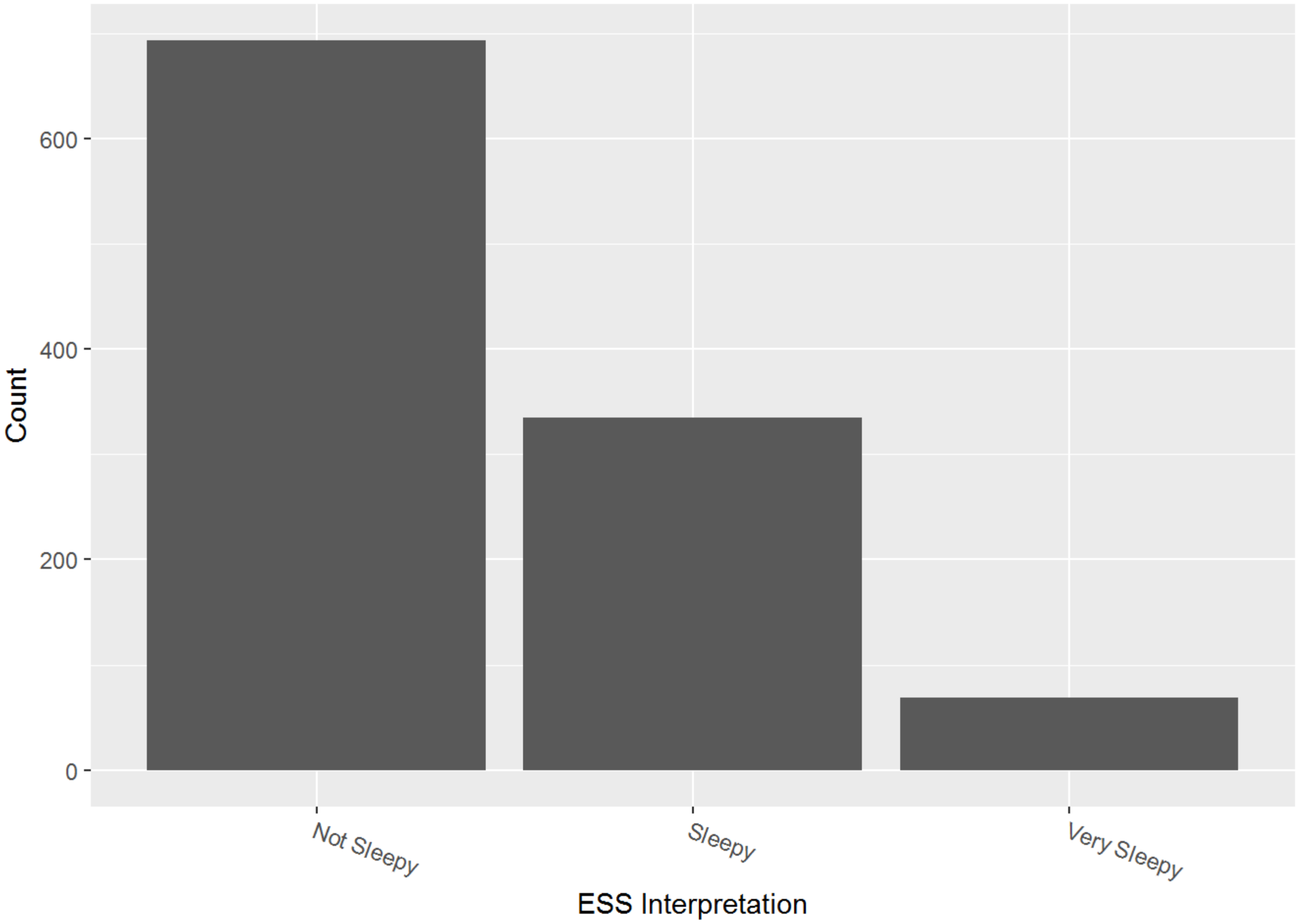
Count
600
400
200
0
Not Sleepy
Sleepy
Very Sleepy
ESS Interpretation

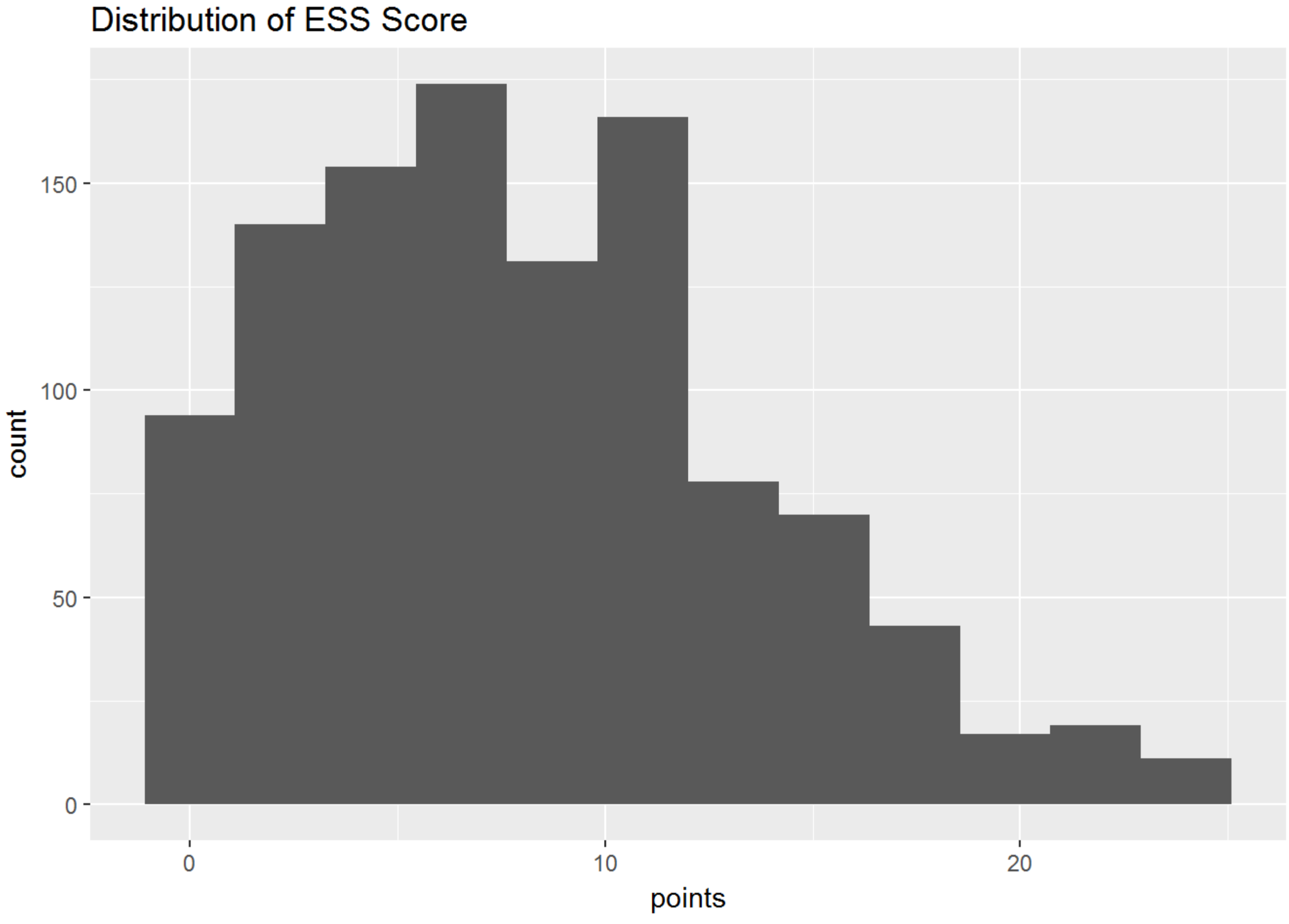
Distribution of ESS Score
count
150
100
50
0
0
10
20
points

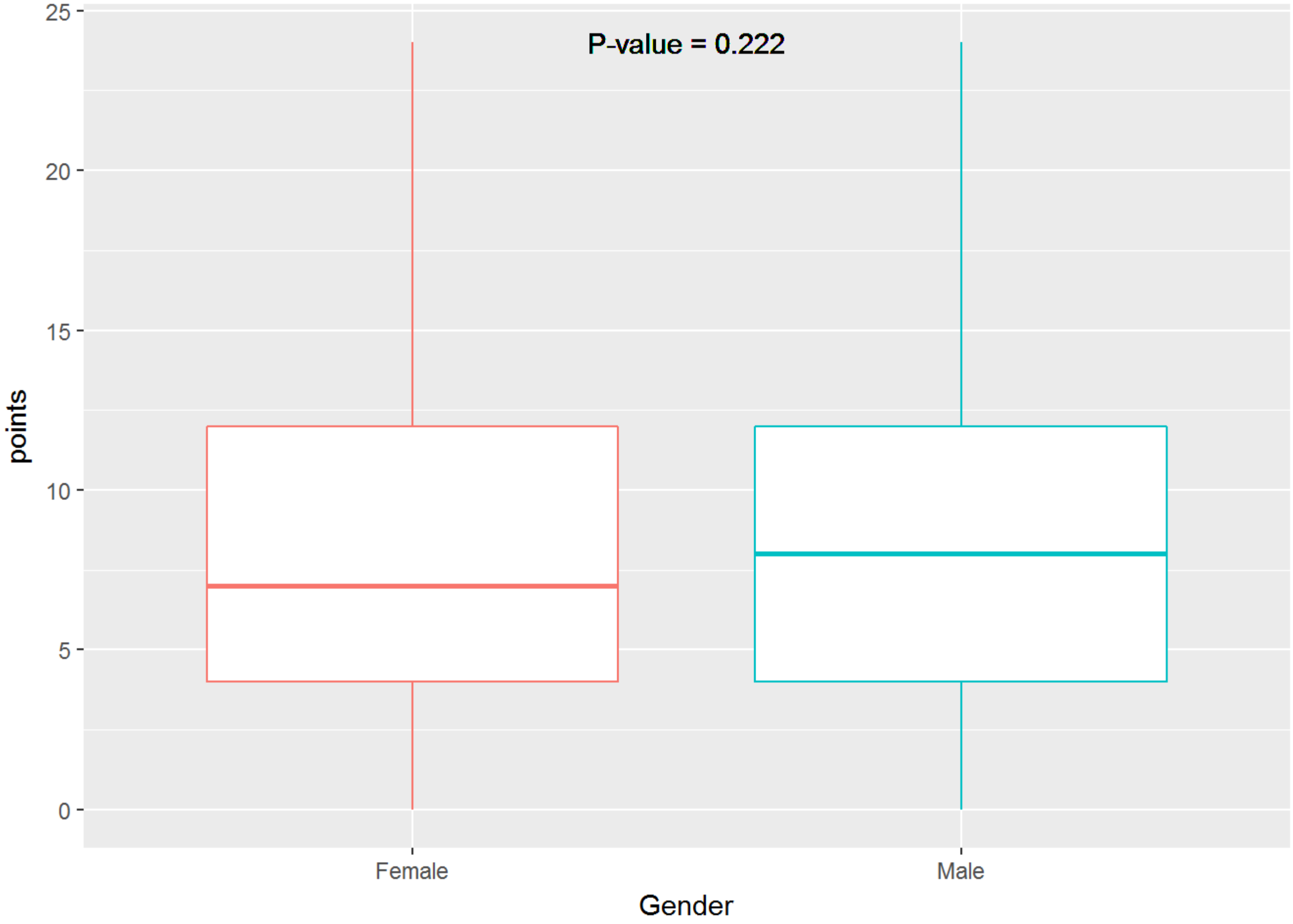

ISI Interpretation

Value	Count	Male	Female
No Clinically Significant Insomnia	153	71	82
Subthreshold Insomnia	342	130	212
Moderately Severe Cinical Insomnia	417	142	275
Severe Clinical Insomnia	176	60	116
NA	69	25	44

Min	Median	Mean	Max	St.Dev	No.Data
0	15	14.95	28	6.425	69

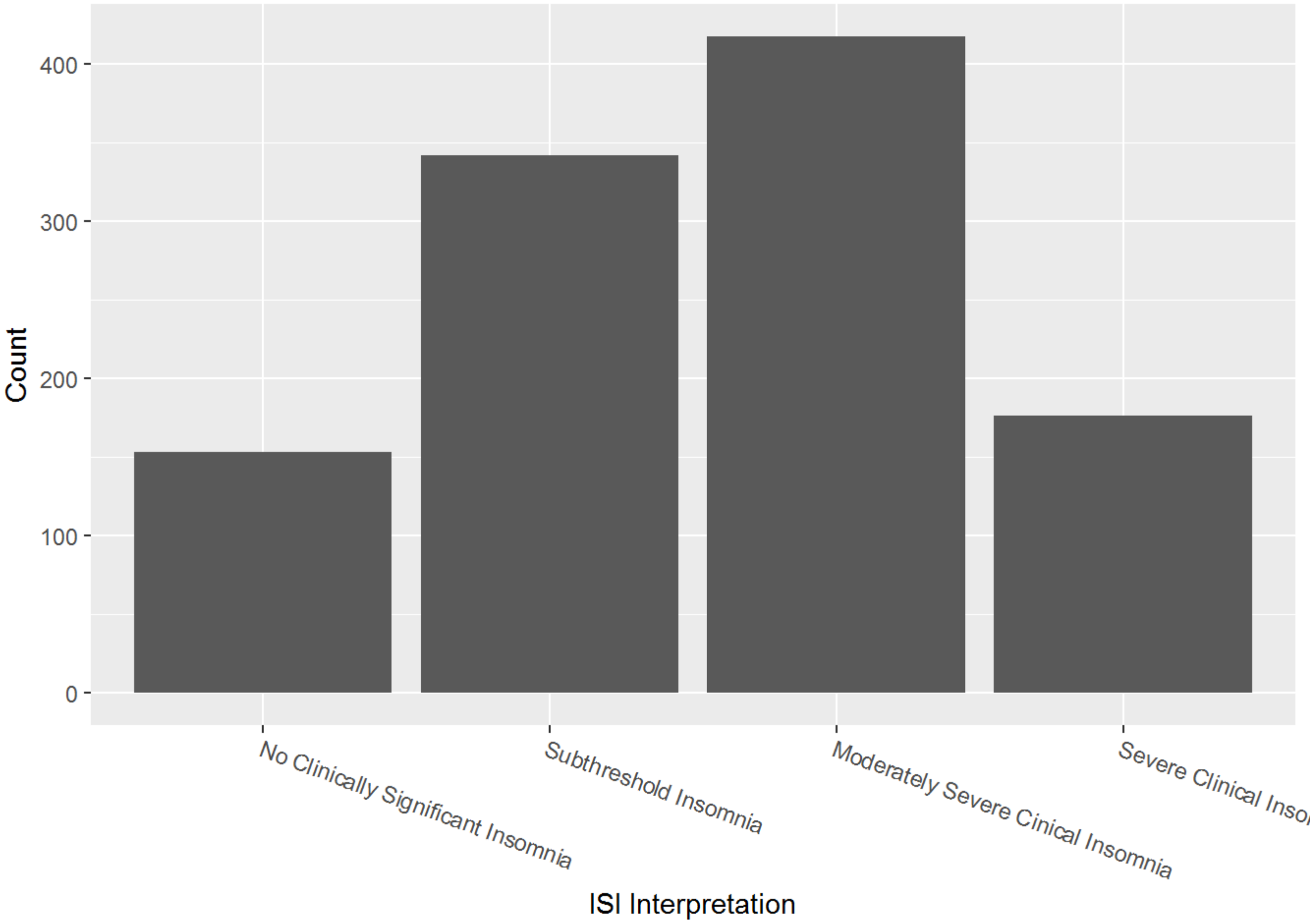
Count
400
300
200
100
0
No Clinically Significant Insomnia
Subthreshold Insomnia
Moderately Severe Cinical Insomnia
Severe Clinical Inso
ISI Interpretation

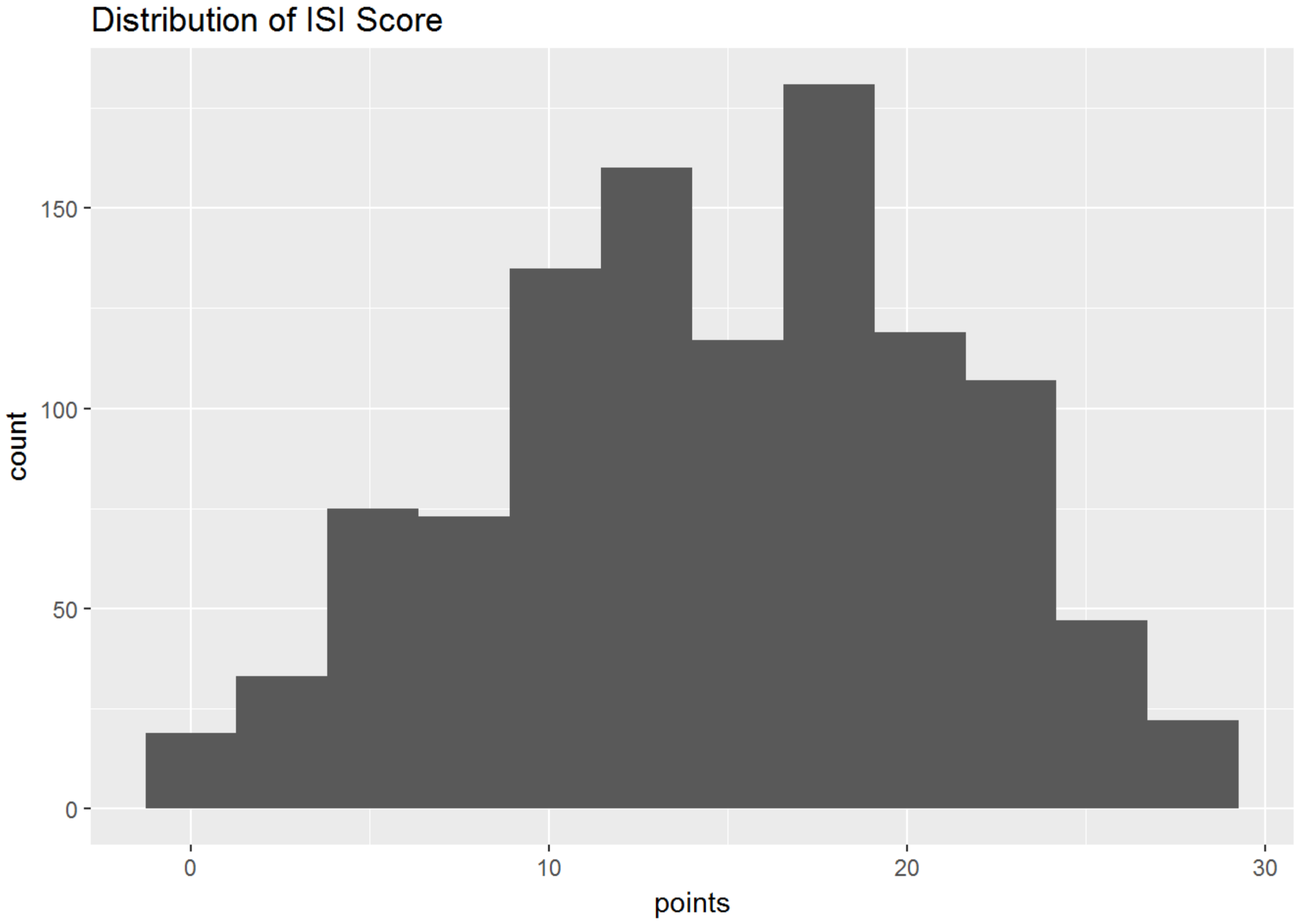
Distribution of ISI Score
count
150
100
50
0
0
10
20
30
points

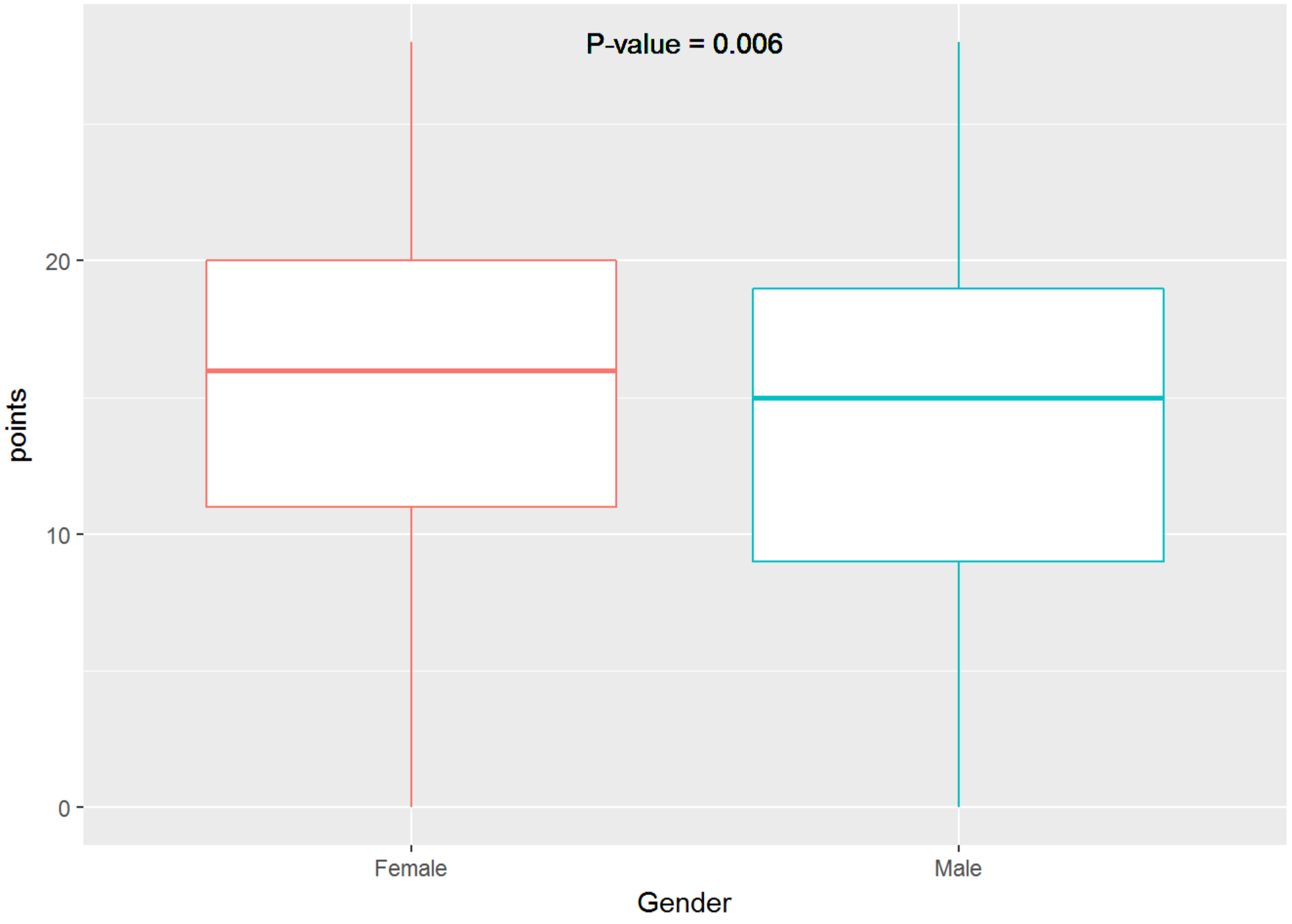

PSQI Interpretation

Value	Count	Male	Female
Good sleep quality	158	76	82
Poor sleep quality	894	319	575
NA	105	33	72

Min	Median	Mean	Max	St.Dev	No.Data
1	10	9.967	21	4.109	105

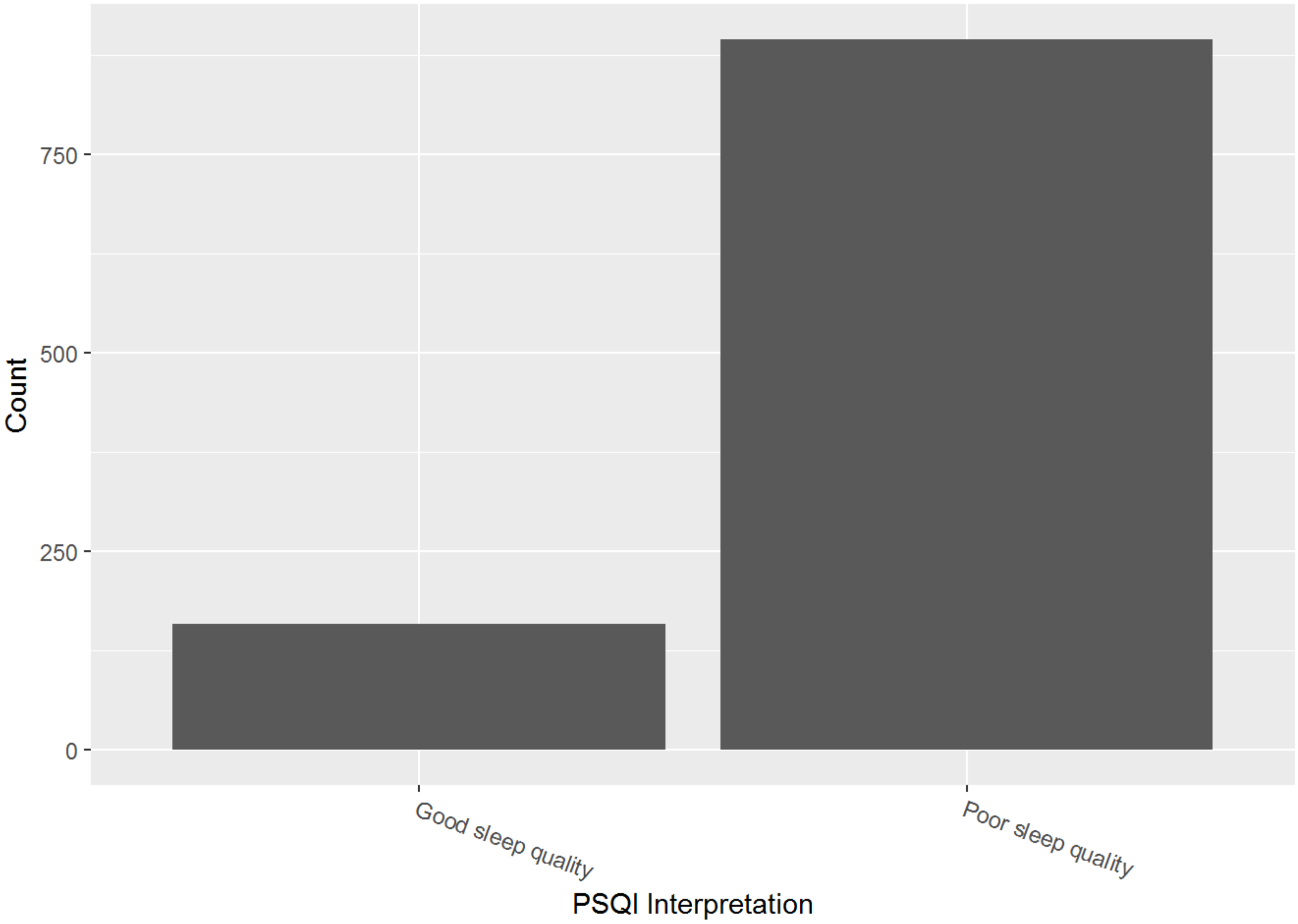
Count
750
500
250
0
Good sleep quality
Poor sleep quality
PSQI Interpretation

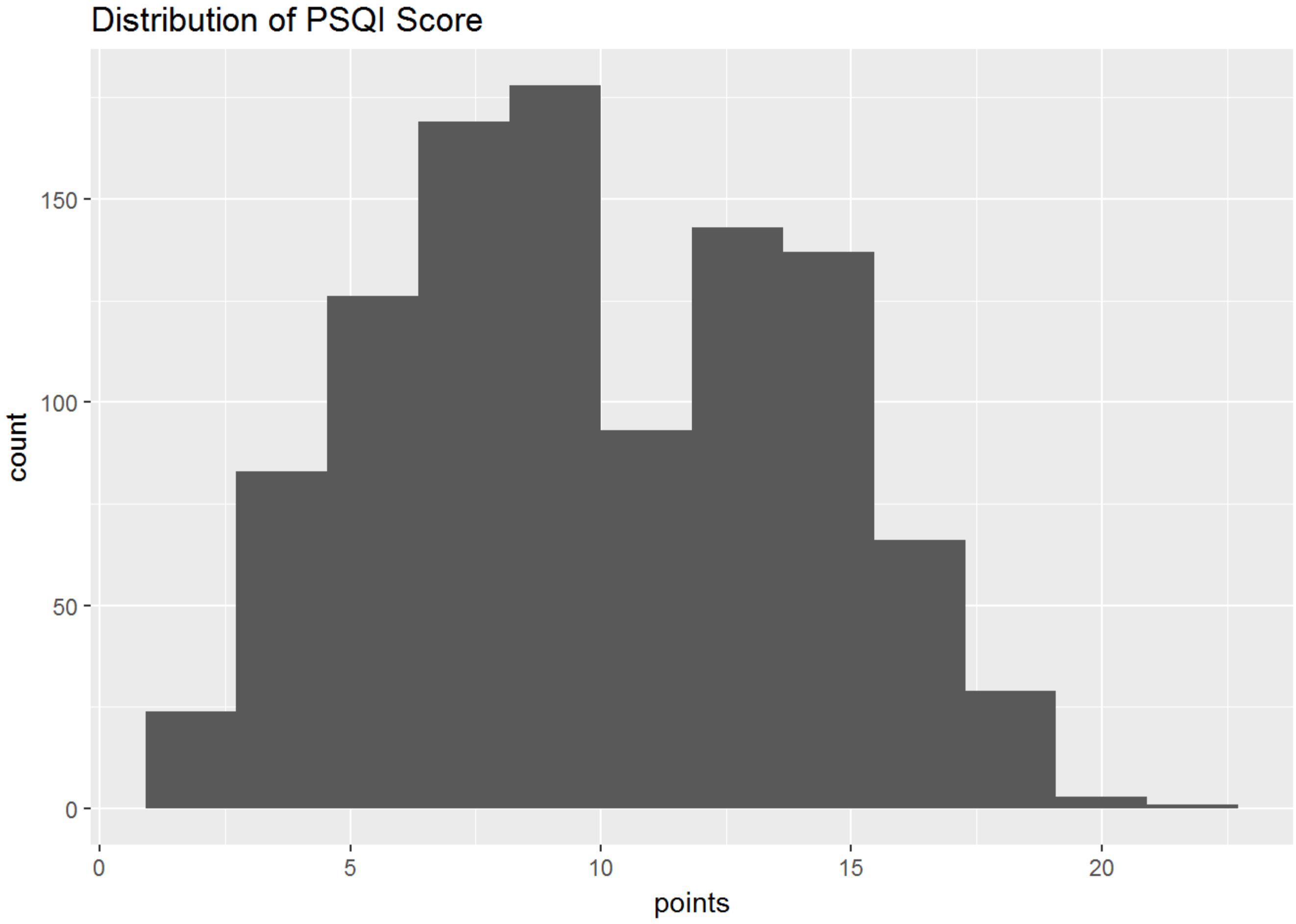
Distribution of PSQI Score
count
150
100
50
0
0
5
10
15
20
points

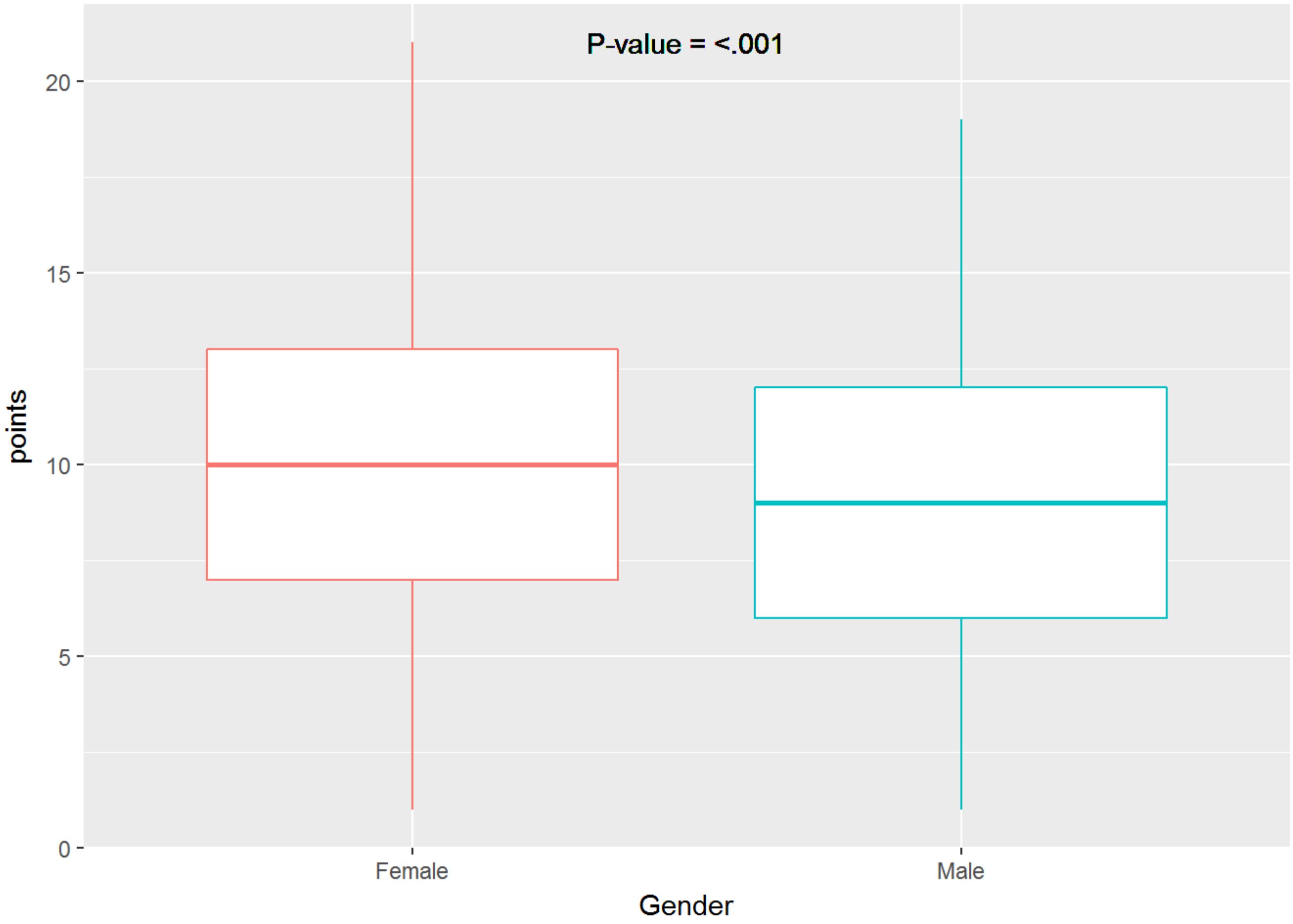

RLS Interpretation

Value	Count	Male	Female
No RLS	12	4	8
Mild RLS	263	120	143
Moderate RLS	536	187	349
Severe RLS	257	85	172
Very Severe RLS	51	19	32
NA	38	13	25

Min	Median	Mean	Max	St.Dev	No.Data
0	15	16.51	39	7.396	38

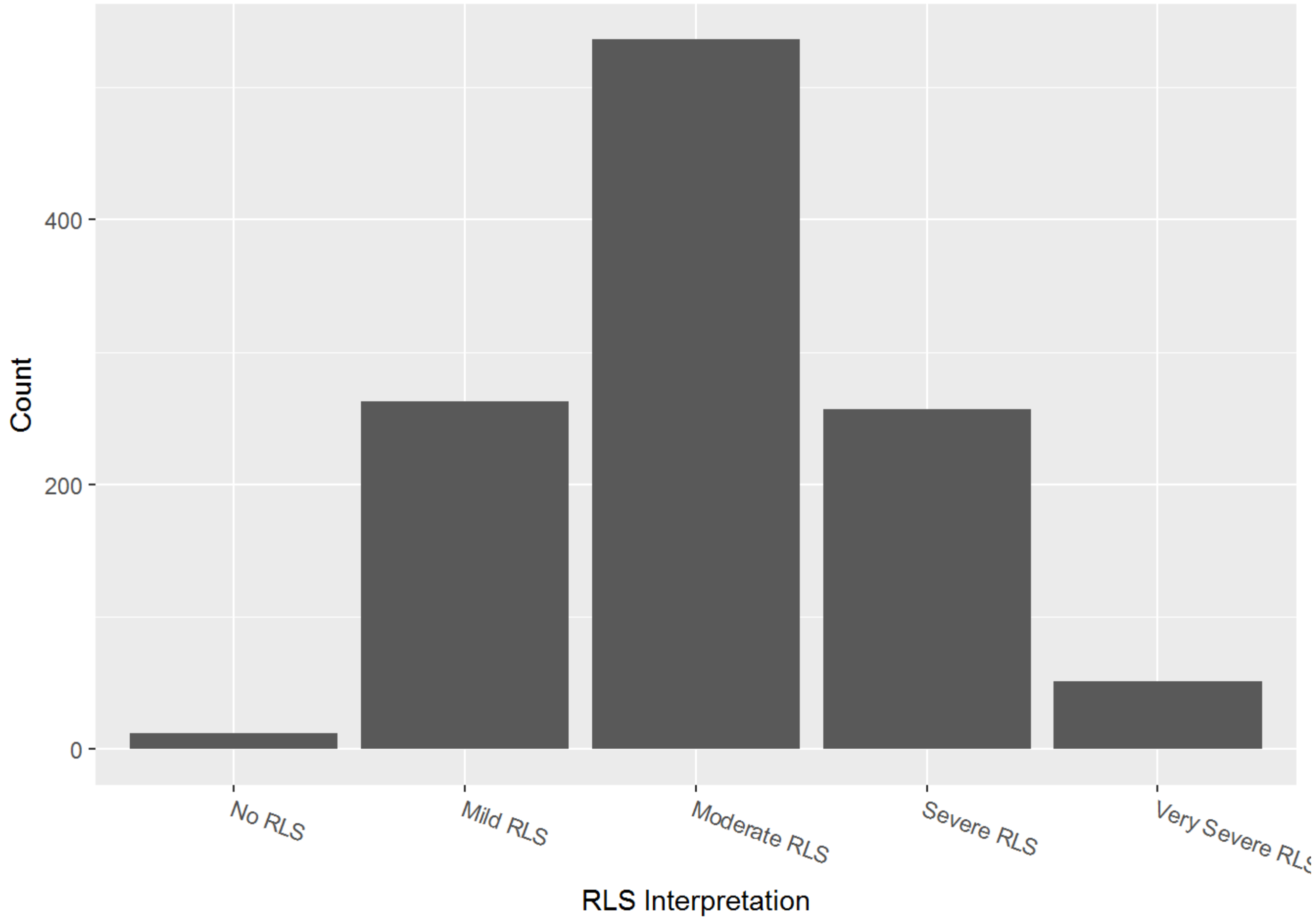
Count
400
200
0
No RLS
Mild RLS
Moderate RLS
Severe RLS
Very Severe RLS
RLS Interpretation

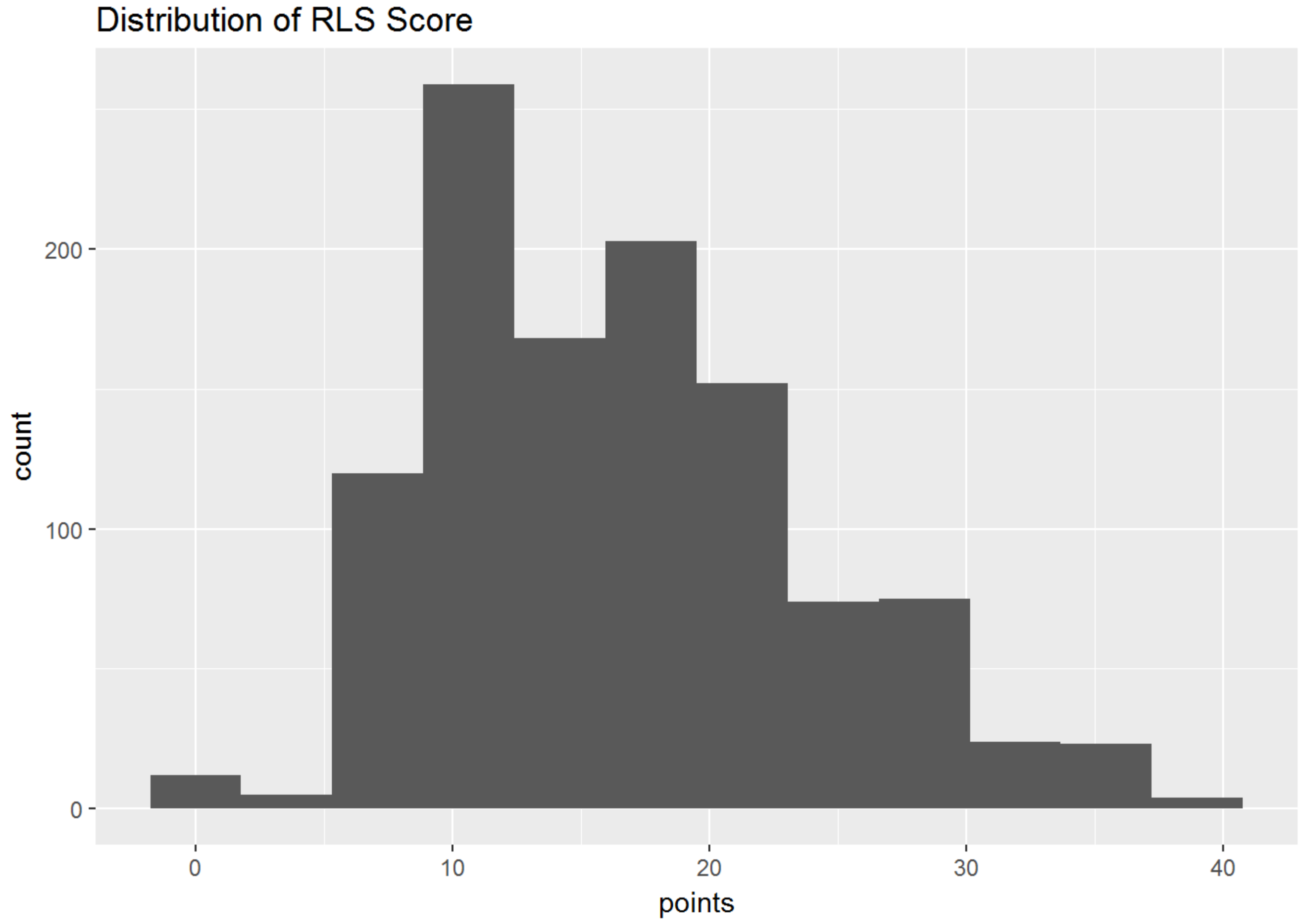
Distribution of RLS Score
count
200
100
0
0
10
20
30
40
points

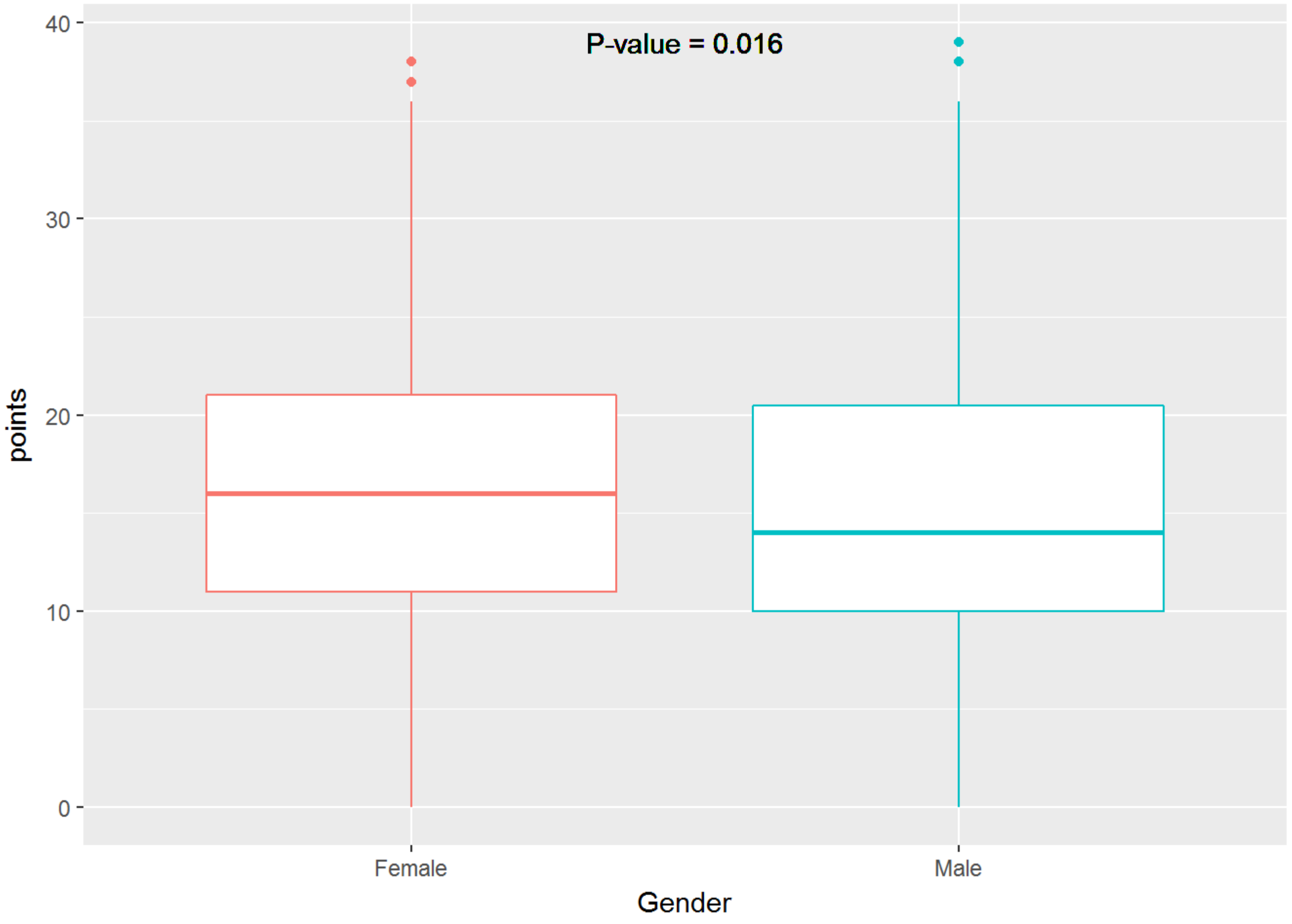

Current Symptoms Sleep Disruptors

Value	Percent	Count	Male	Female
None	43.4	502	225	277
Anxiety	18.1	209	62	147
Other	14.3	166	56	110
Pain	13.1	152	36	116
Noise	8.1	94	28	66
Racing thoughts	7.5	87	25	62
Room temperature	6.7	77	17	60
Bed partner (snoring, movements)	6.4	74	22	52
Pets	6.1	71	21	50
Sadness/depression	4.2	49	16	33
Leg discomfort	1.8	21	6	15
Uncomfortable bed	1.7	20	11	9
Snoring	1.6	19	7	12
Awakening to urinate	1	11	3	8
Leg movements	0.8	9	2	7
Dry mouth	0.6	7	1	6
Stop breathing	0.6	7	3	4

Irritability	0.5	6	1	5
Leg cramps	0.4	5	1	4
Vivid dreams	0.4	5	1	4
Gasping	0.3	3	0	3
Grinding teeth	0.3	4	1	3
Heart racing	0.3	4	1	3
Morning headache	0.3	4	1	3
Nasal congestion	0.3	4	1	3
Talking in sleep	0.3	3	0	3
Afraid of not being able to sleep	0.2	2	0	2
Hallucination as falling sleep or awakening	0.2	2	0	2
Heartburn	0.2	2	0	2
Muscular tension	0.2	2	0	2
Eating in the night	0.1	1	0	1
Esophageal reflux	0.1	1	0	1
Night terrors	0.1	1	0	1
Other unusual movements	0.1	1	1	0
Screaming, shouting or acting out dreams	0.1	1	1	0
Unable to move before falling asleep or after awakening	0.1	1	0	1
Waking up confused	0.1	1	0	1

Current Symptoms Breathing

Value	Percent	Count	Male	Female
Snoring	66	764	300	464
Awakening to urinate	55.1	637	233	404
Dry mouth	37.2	430	166	264
Morning headache	24.4	282	72	210
Stop breathing during sleep	22.9	265	126	139
Nasal congestion	21.4	248	80	168
Gasping	17.1	198	79	119
Heartburn	11.4	132	53	79
Reflux	9.1	105	41	64
Choking	8.6	100	36	64
None	7.4	86	27	59
Heart racing	6.7	77	22	55
Shortness of breath	4.2	49	15	34
Chest pain	1.2	14	2	12
Other	0.6	7	1	6
Heart slowing	0.2	2	1	1

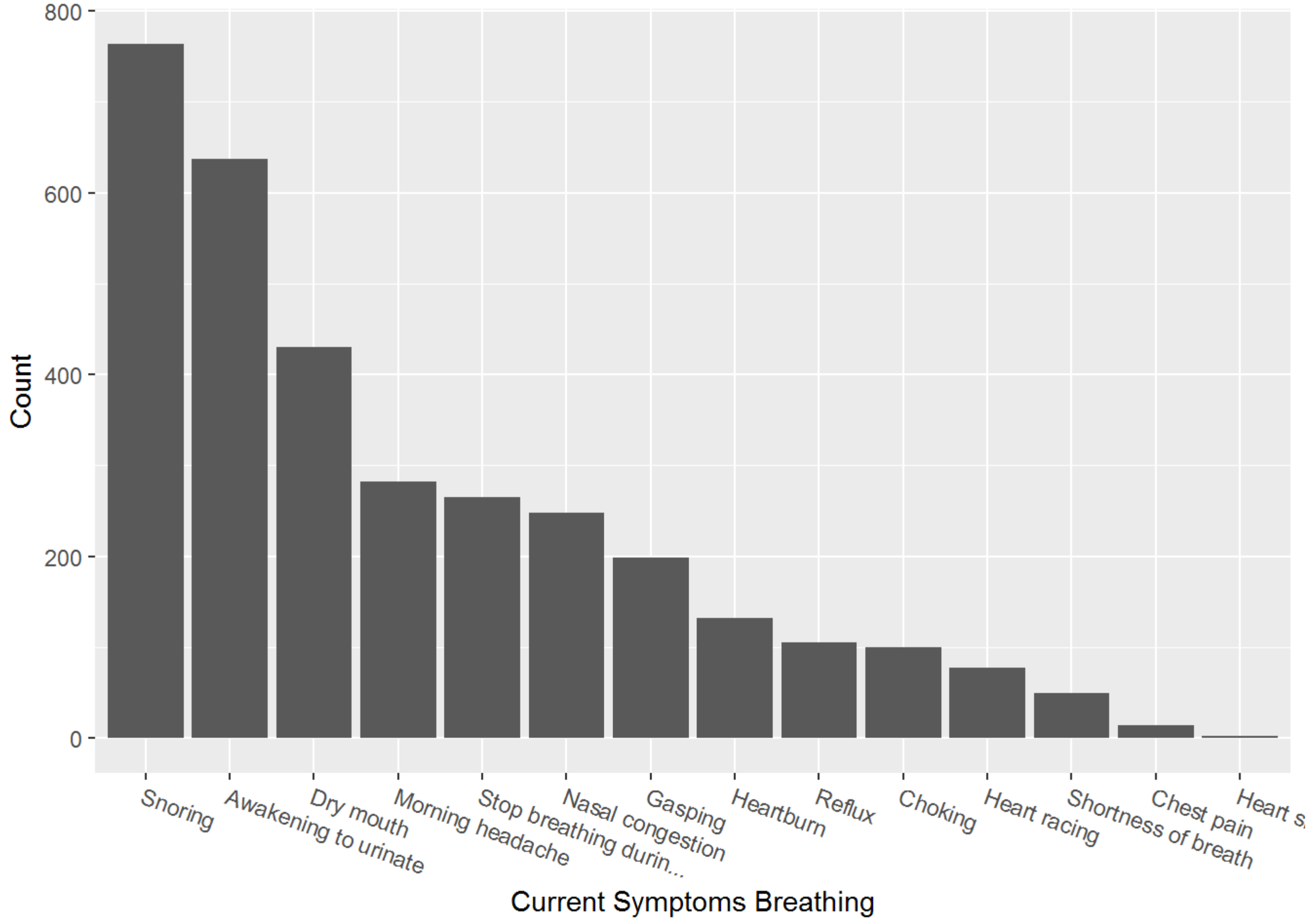

Correlation Table

	Awakening to urinate	Chest pain	Choking	Dry mouth	Gasping	Heart racing	Heart slowing	Heartburn	Morning headache	Nasal congestion	Reflux	Shortness of breath	Snoring	Stop breathing durin...
Awakening to urinate	1	0.02	0.05	0.1	0.02	0.03	0.04	0.11	0.07	0.01	0.07	-0.01	0.13	0.07
Chest pain	0.02	1	0.08	-0.02	0.05	0.07	0	0.11	0.07	0	0.08	0.06	0.03	0
Choking	0.05	0.08	1	0.11	0.37	0.07	-0.01	0.07	0.1	0.09	0.11	0.07	0.13	0.25
Dry mouth	0.1	-0.02	0.11	1	0.14	0.07	0.05	0.16	0.2	0.15	0.07	0.04	0.18	0.16
Gasping	0.02	0.05	0.37	0.14	1	0.18	-0.02	0.08	0.13	0.09	0.1	0.16	0.2	0.27
Heart racing	0.03	0.07	0.07	0.07	0.18	1	-0.01	0.07	0.13	0.05	0.05	0.05	0.05	0.04
Heart slowing	0.04	0	-0.01	0.05	-0.02	-0.01	1	-0.01	-0.02	0.03	0.06	-0.01	0.03	-0.02
Heartburn	0.11	0.11	0.07	0.16	0.08	0.07	-0.01	1	0.18	0.05	0.3	0.02	0.13	0.1
Morning headache	0.07	0.07	0.1	0.2	0.13	0.13	-0.02	0.18	1	0.16	0.06	0.04	0.09	0.09
Nasal congestion	0.01	0	0.09	0.15	0.09	0.05	0.03	0.05	0.16	1	0.03	0.04	0.12	0.08
Reflux	0.07	0.08	0.11	0.07	0.1	0.05	0.06	0.3	0.06	0.03	1	0.05	0.04	0.06
Shortness of breath	-0.01	0.06	0.07	0.04	0.16	0.05	-0.01	0.02	0.04	0.04	0.05	1	0.08	0.11
Snoring	0.13	0.03	0.13	0.18	0.2	0.05	0.03	0.13	0.09	0.12	0.04	0.08	1	0.35
Stop breathing durin...	0.07	0	0.25	0.16	0.27	0.04	-0.02	0.1	0.09	0.08	0.06	0.11	0.35	1

Current Symptoms Limb

Value	Percent	Count	Male	Female
Leg discomfort	85.6	990	356	634
Leg movements	28.1	325	130	195
Leg cramps	17.5	202	65	137
Teeth grinding	17	197	62	135
Other unusual movements or discomfort	3.9	45	15	30
Muscular tension	2.5	29	10	19
None	1.4	16	6	10

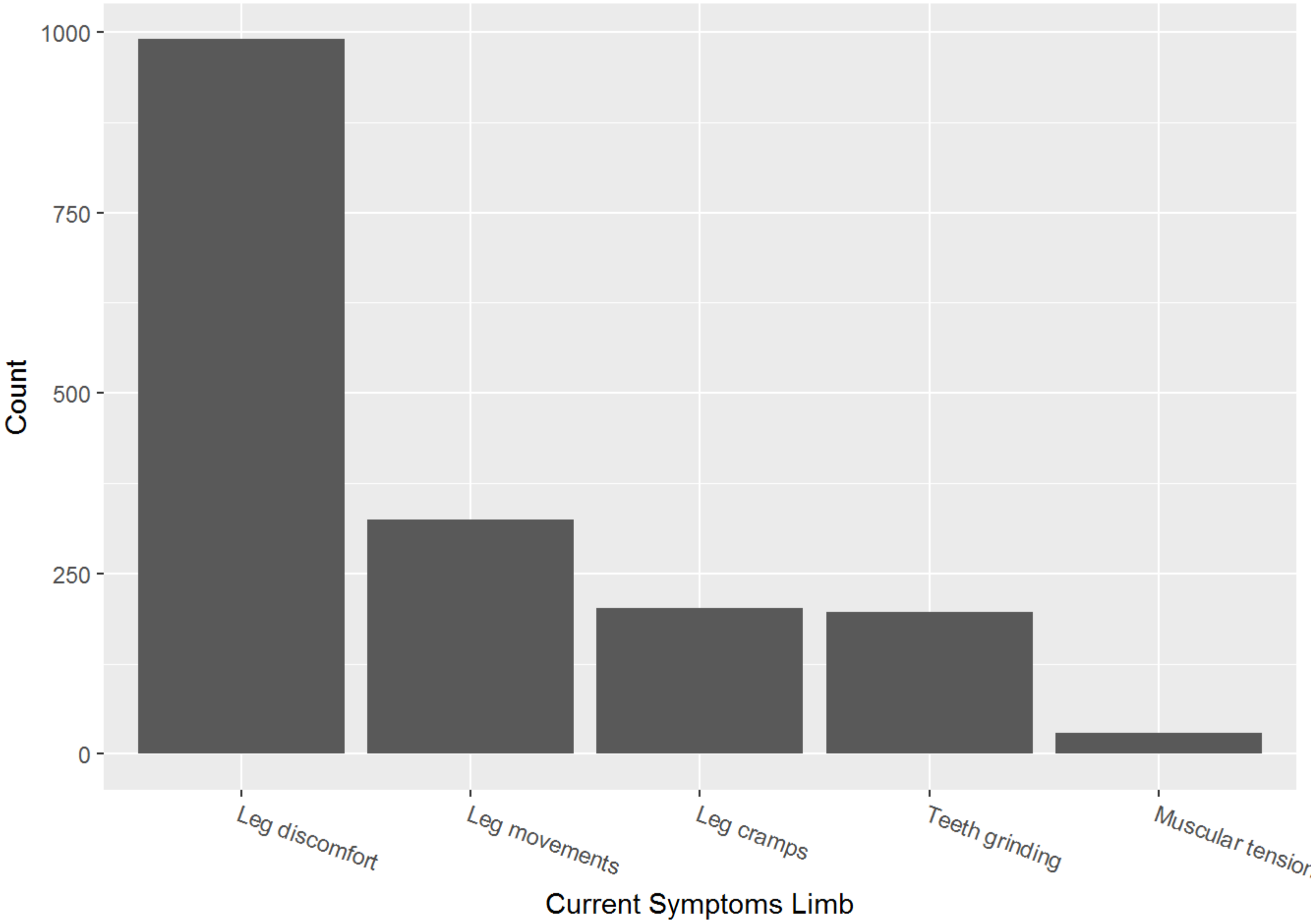

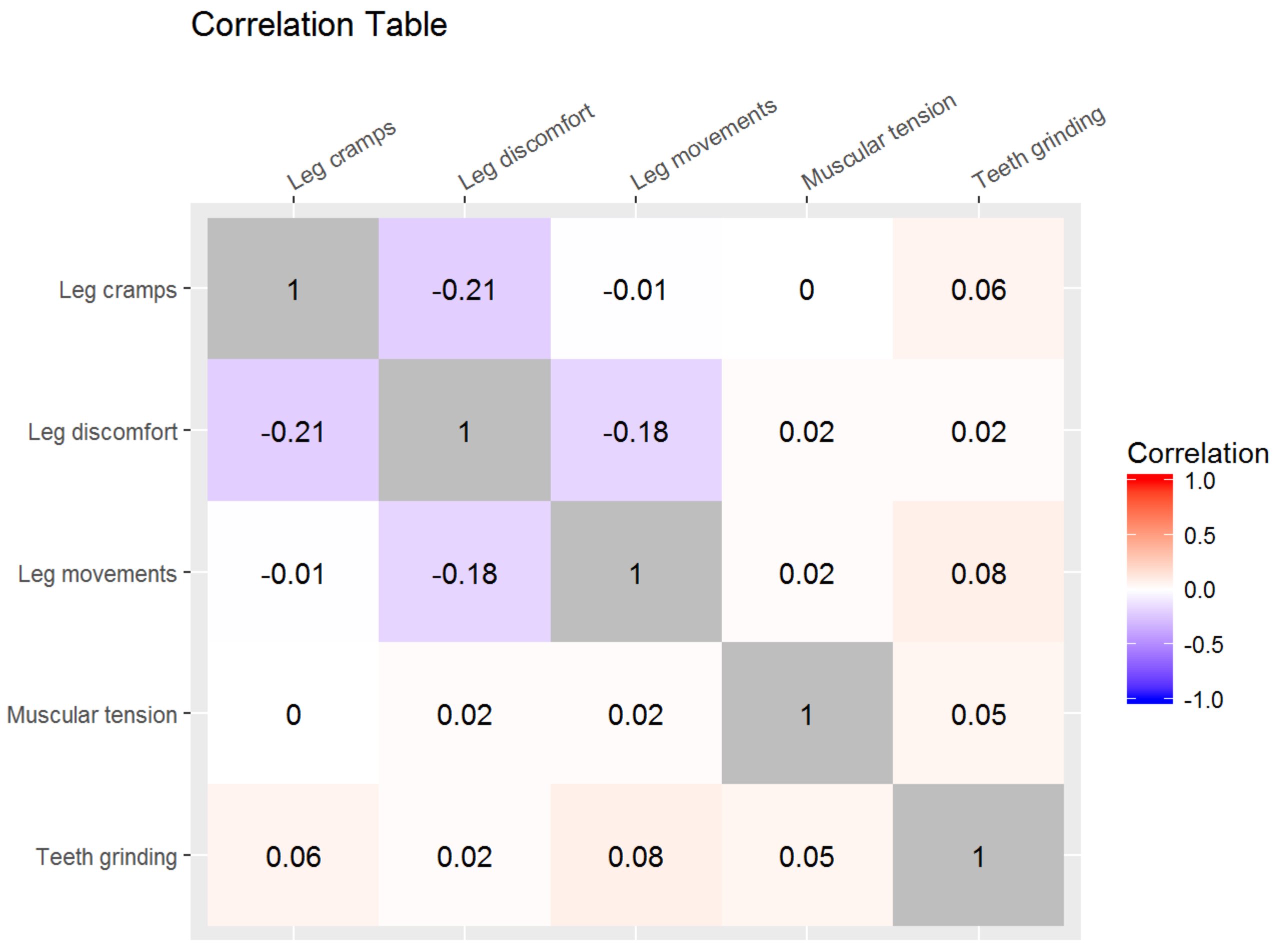

Current Symptoms RLS

Value	Percent	Count	Male	Female
Urge to move legs accompanied by unpleasant sensation	97.6	1129	415	714
Urge to move legs worsen during rest	97.6	1129	416	713
Urge to move legs relieved by movement	97.4	1127	415	712
Urge to move legs worse during evenings	97.3	1126	414	712
None	0.1	1	0	1

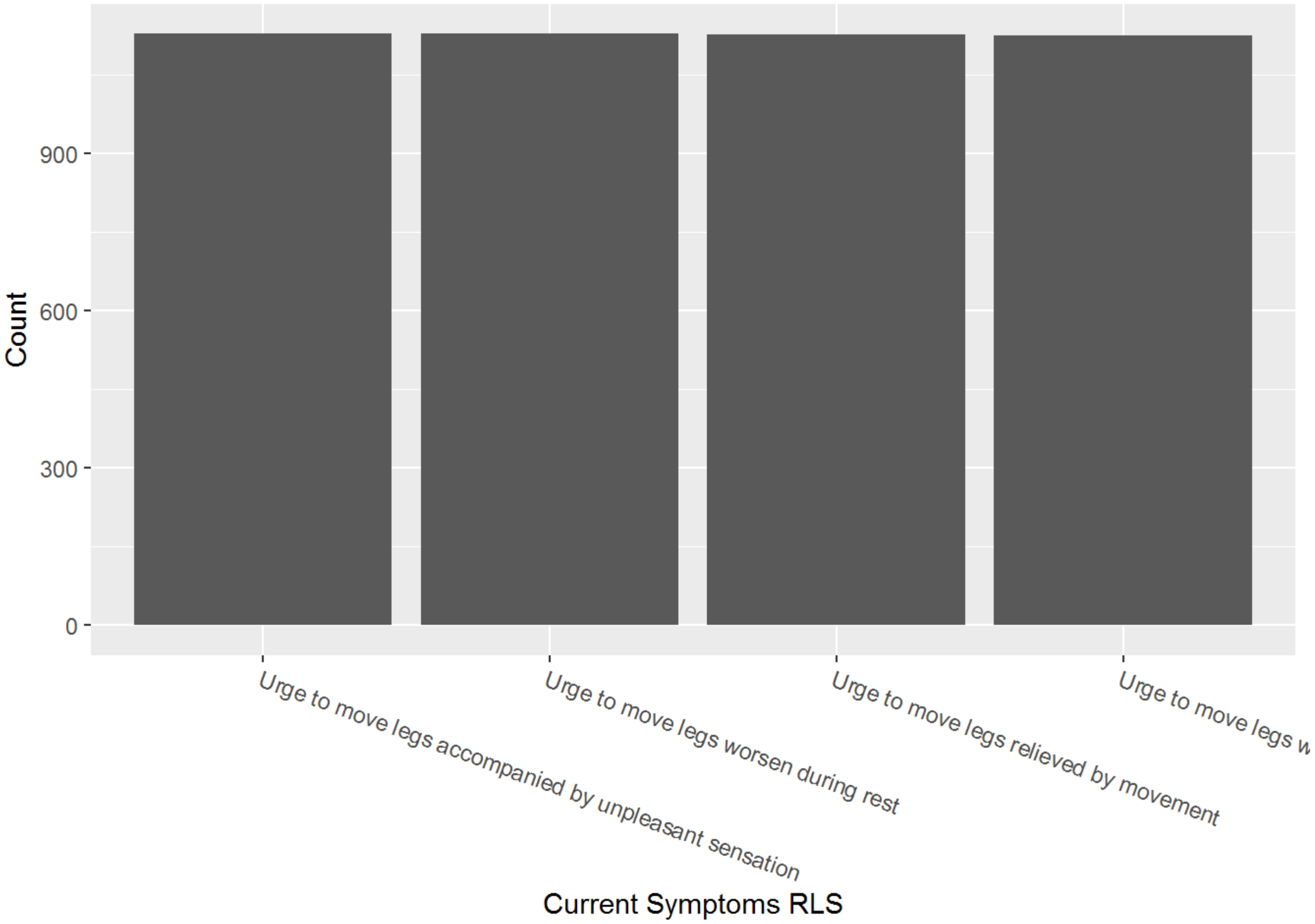

Current Symptoms Insomnia

Value	Percent	Count	Male	Female
Difficulty staying asleep	52.5	607	191	416
Unrefreshing sleep	48	555	196	359
Difficulty falling asleep	42.4	490	149	341
None	17.1	198	93	105
Early awakening	9.9	115	34	81
Afraid of not being able to sleep	5.1	59	18	41
Other	0.3	4	2	2

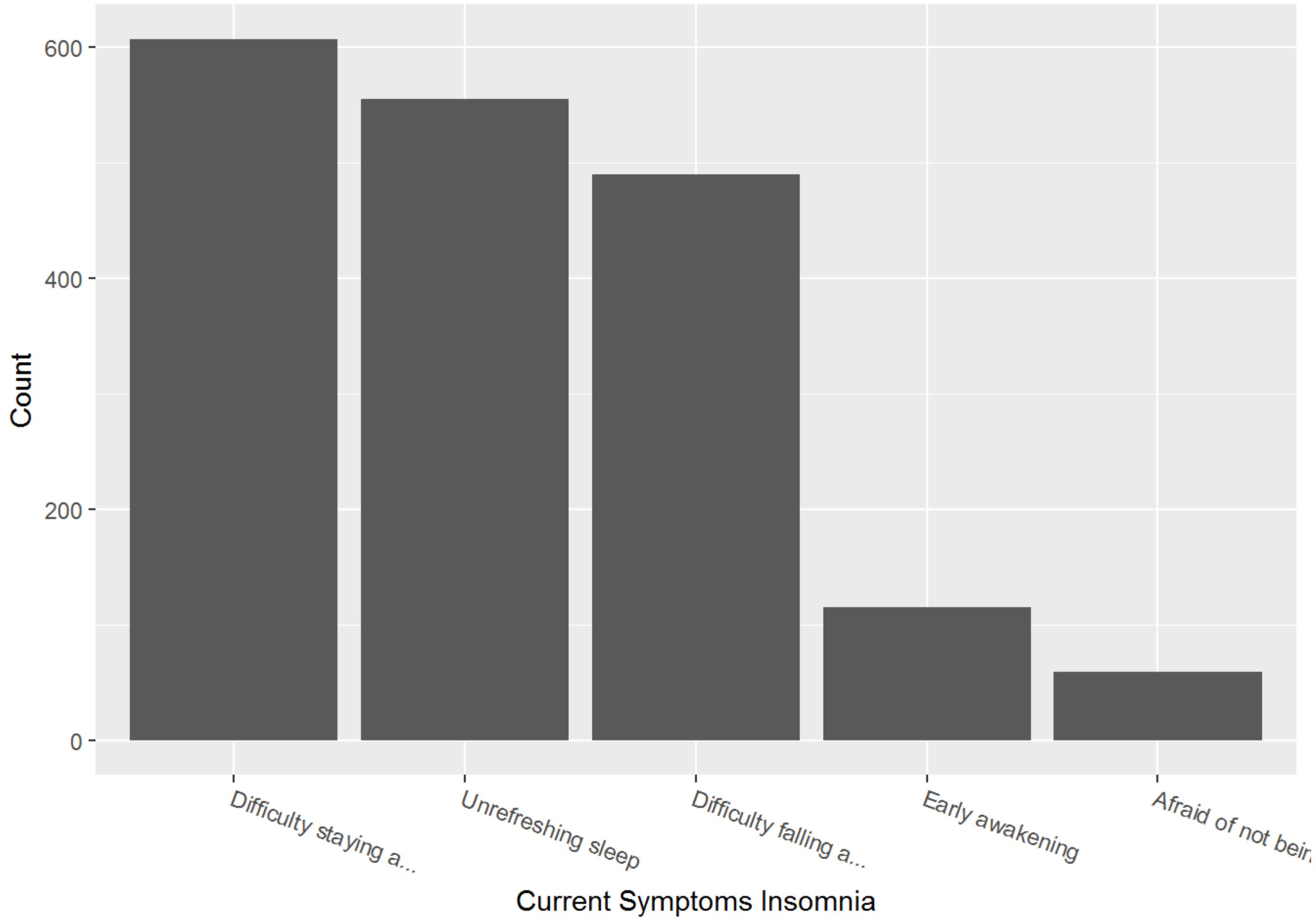
Count
600
400
200
0
Difficulty staying a...
Unrefreshing sleep
Difficulty falling a...
Early awakening
Afraid of not bein
Current Symptoms Insomnia

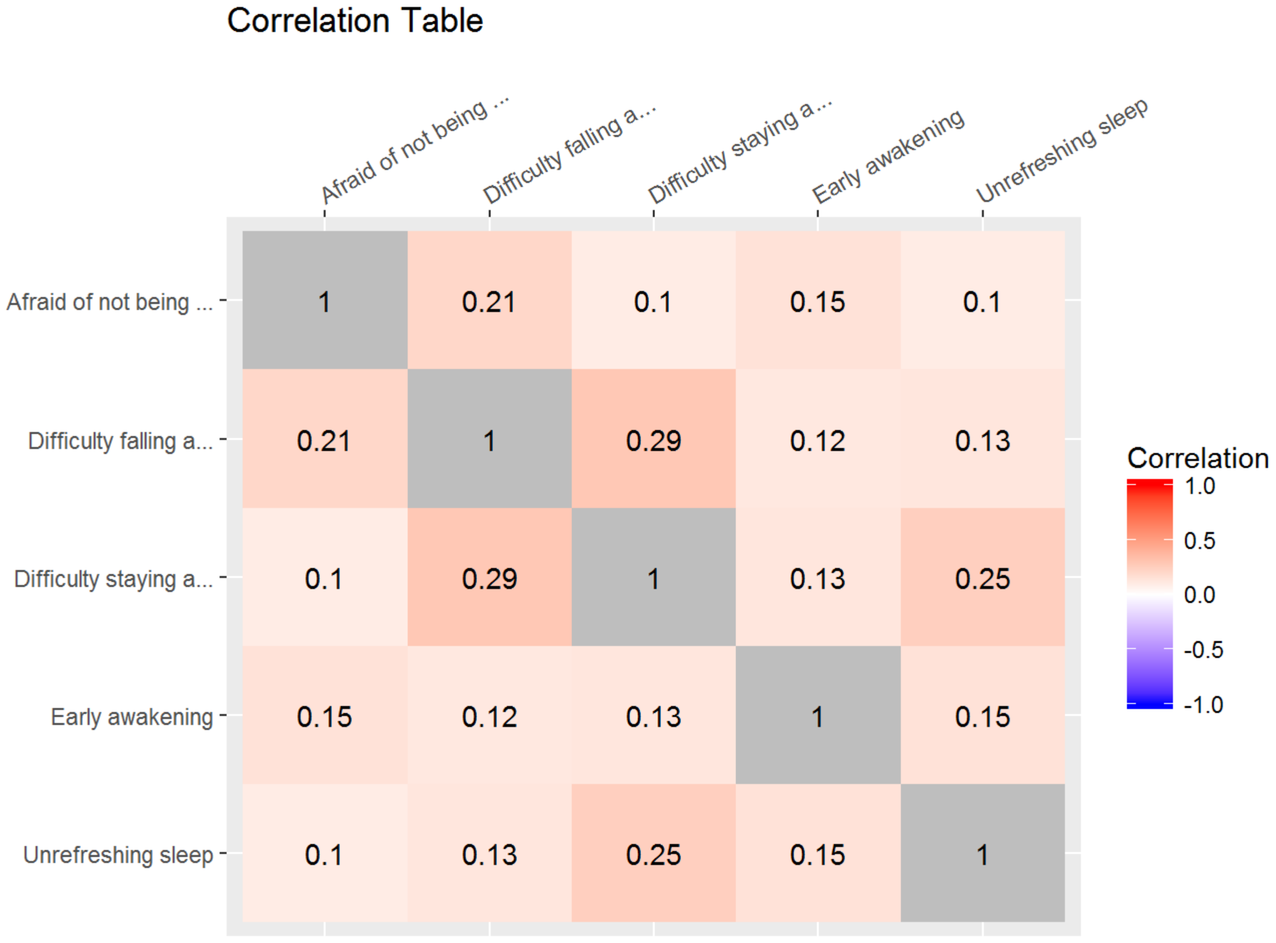
Correlation Table
Afraid of not being ...
Difficulty falling a...
Difficulty staying a...
Early awakening
Unrefreshing sleep
Afraid of not being ... 1 0.21 0.1 0.15 0.1
Difficulty falling a... 0.21 1 0.29 0.12 0.13
Difficulty staying a... 0.1 0.29 1 0.13 0.25
Early awakening 0.15 0.12 0.13 1 0.15
Unrefreshing sleep 0.1 0.13 0.25 0.15 1
Correlation
1.0
0.5
0.0
-0.5
-1.0

Current Symptoms REM

Value	Percent	Count	Male	Female
None	63.6	736	262	474
Talking in sleep	14	162	63	99
Vivid dreams	11.8	136	55	81
Nightmares	9.3	108	31	77
Screaming, shouting, or acting out dreams	6.1	71	40	31
Walking in sleep	2.6	30	12	18
Wake up confused	1.6	19	11	8
Injury to self or others during sleep	1.4	16	9	7
Eating in the night	1.2	14	2	12
Other	1	12	4	8
Night terrors	0.8	9	3	6
Bedwetting	0.3	4	2	2
Violent upon being awakened	0.3	4	4	0

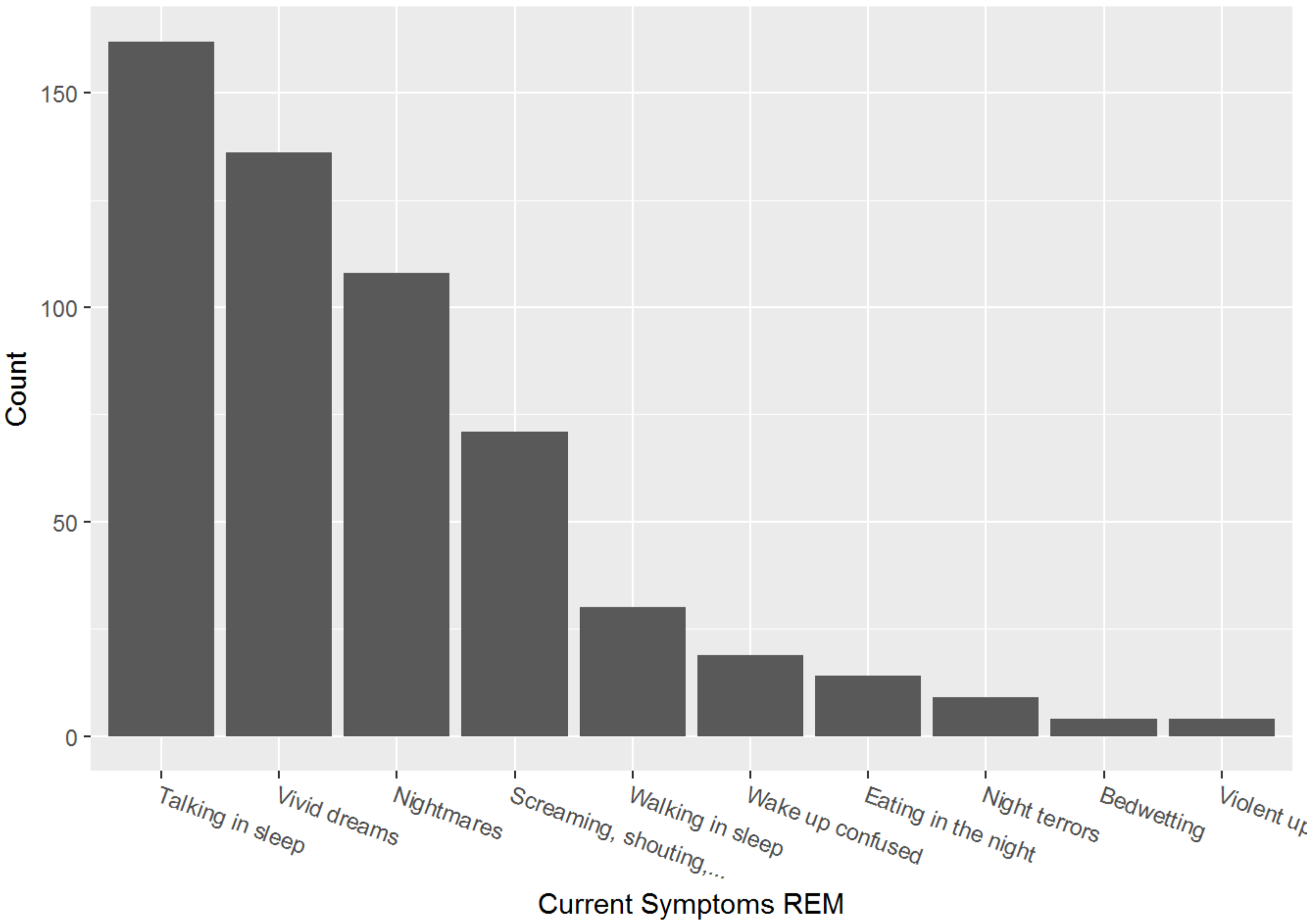

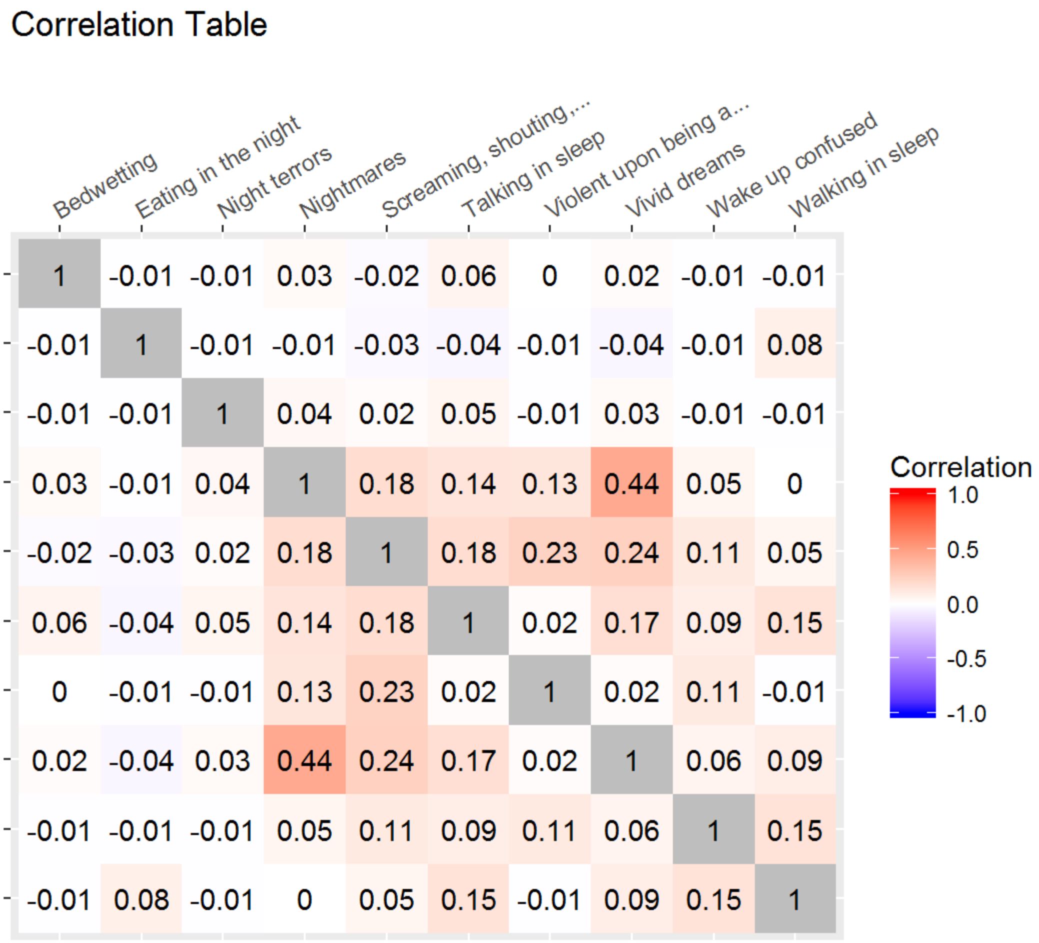

Current Symptoms Awake

Value	Percent	Count	Male	Female
Fatigue	60.2	696	239	457
Denies having accidents or near miss accidents related to sleepiness	56	648	231	417
Difficulty concentrating	30.2	349	131	218
Unintentional naps	23.5	272	114	158
Discomfort in legs (or arms) at rest	13.6	157	59	98
None	11.3	131	57	74
Accidents or near accident due to sleepiness	6	69	34	35
Sleep attacks	3.5	40	15	25
Discomfort in legs at rest	1.1	13	4	9
Other	0.5	6	2	4
Sudden weakness upon laughter, anger, or surprise	0.1	1	1	0

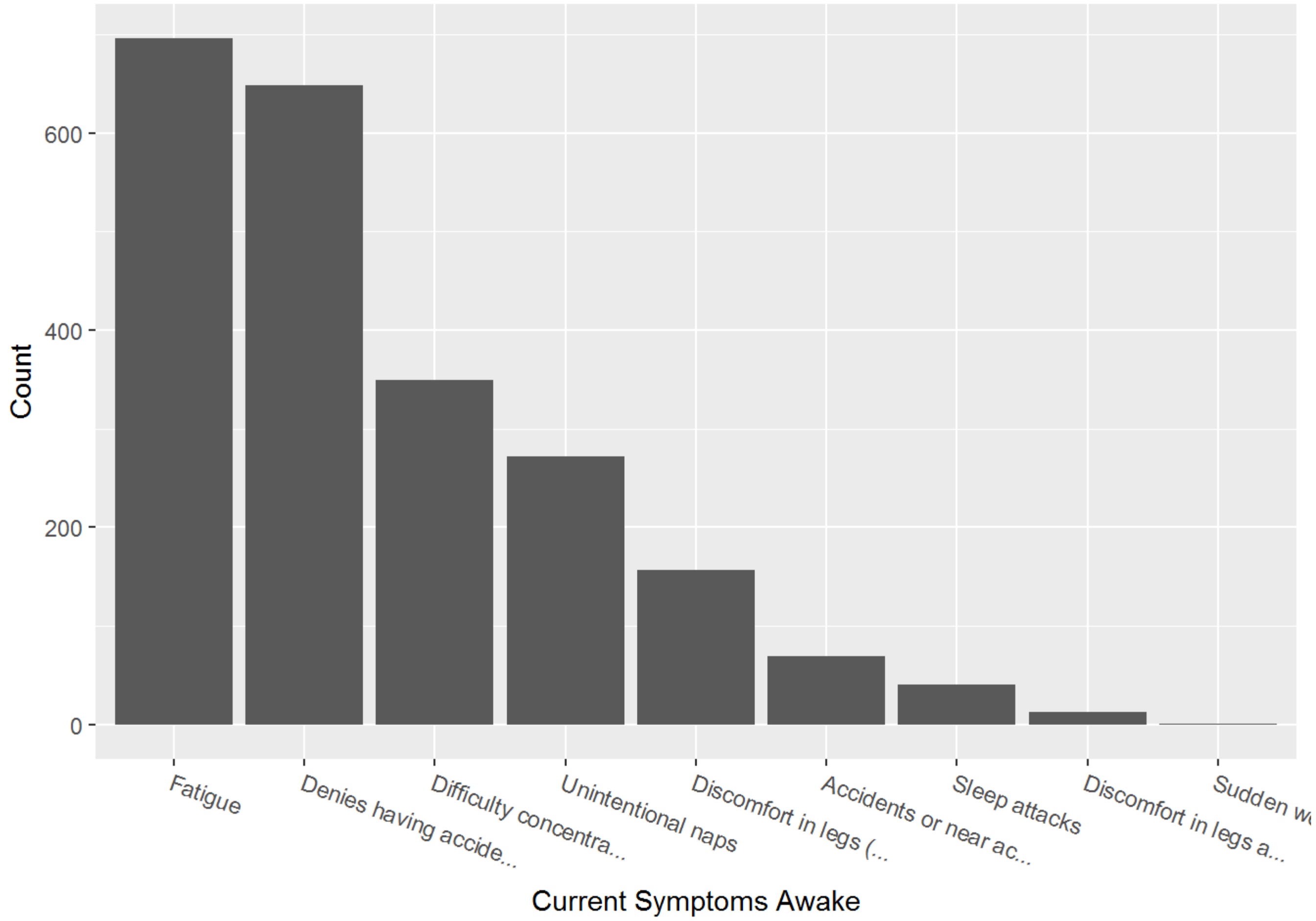

Correlation Table

	Accidents or near ac...	Denies having accide...	Difficulty concentra...	Discomfort in legs (...	Discomfort in legs a...	Fatigue	Sleep attacks	Sudden weakness upon...	Unintentional naps
Accidents or near ac...	1	-0.28	0.14	0.04	-0.03	0.05	0.15	-0.01	0.2
Denies having accide...	-0.28	1	-0.11	0.02	-0.12	-0.13	-0.01	-0.03	0.02
Difficulty concentra...	0.14	-0.11	1	0.09	0	0.36	0.12	0.04	0.23
Discomfort in legs (...	0.04	0.02	0.09	1	-0.04	0	0.09	-0.01	0.01
Discomfort in legs a...	-0.03	-0.12	0	-0.04	1	0.02	-0.02	0.28	0.02
Fatigue	0.05	-0.13	0.36	0	0.02	1	-0.01	0.02	0.18
Sleep attacks	0.15	-0.01	0.12	0.09	-0.02	-0.01	1	-0.01	0.25
Sudden weakness upon...	-0.01	-0.03	0.04	-0.01	0.28	0.02	-0.01	1	0.05
Unintentional naps	0.2	0.02	0.23	0.01	0.02	0.18	0.25	0.05	1

Correlation: 1.0, 0.5, 0.0, -0.5, -1.0

Current Symptoms Narcolepsy

Value	Percent	Count	Male	Female
Daytime sleepiness	48.9	566	207	359
None	43.8	507	189	318
Unable to move before falling asleep or after awakening	4.8	55	18	37
Hallucination upon falling sleep or awakening	3.5	41	19	22
Sudden weakness after surprise or laughter	1	12	6	6
Other	0.1	1	0	1

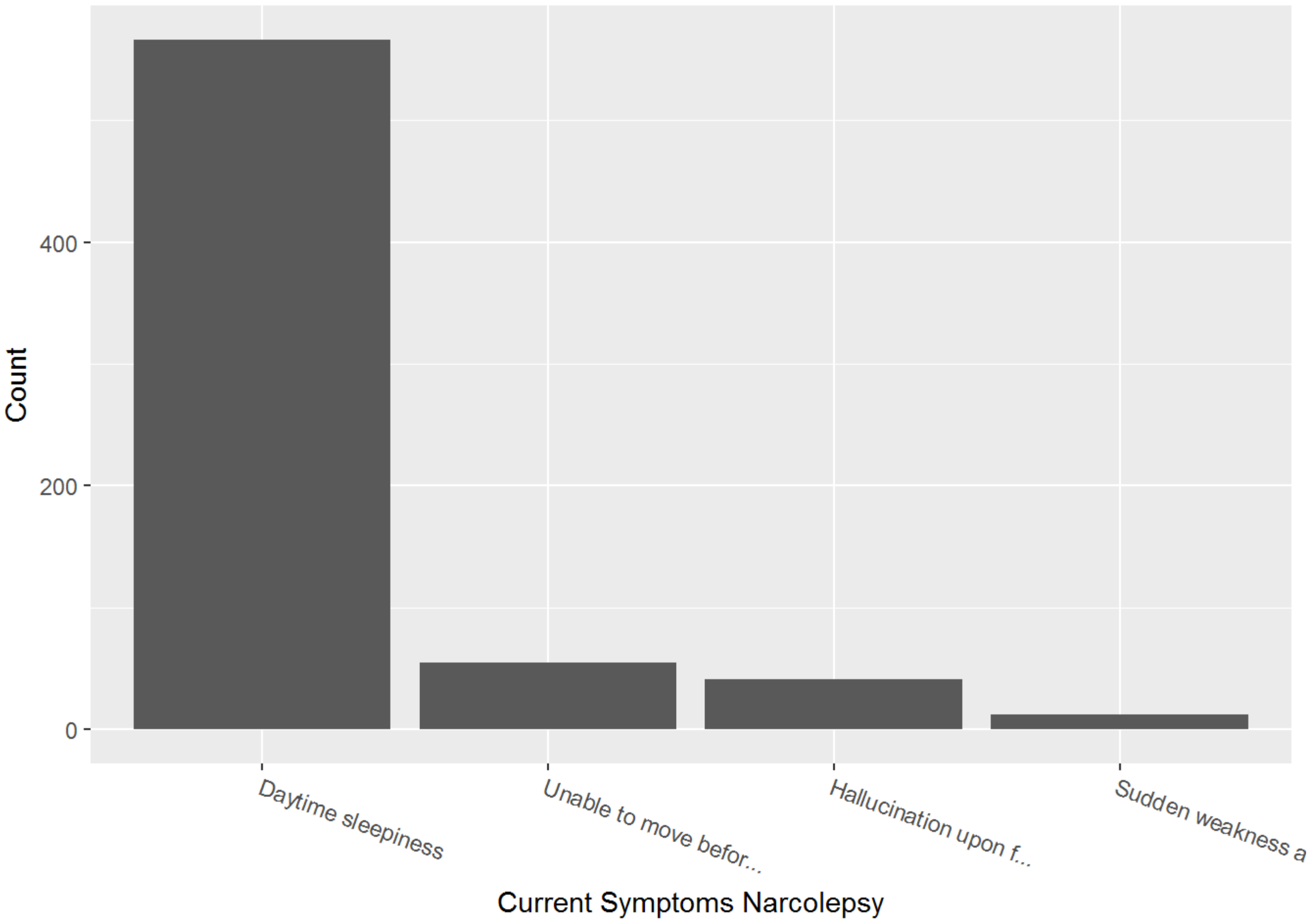

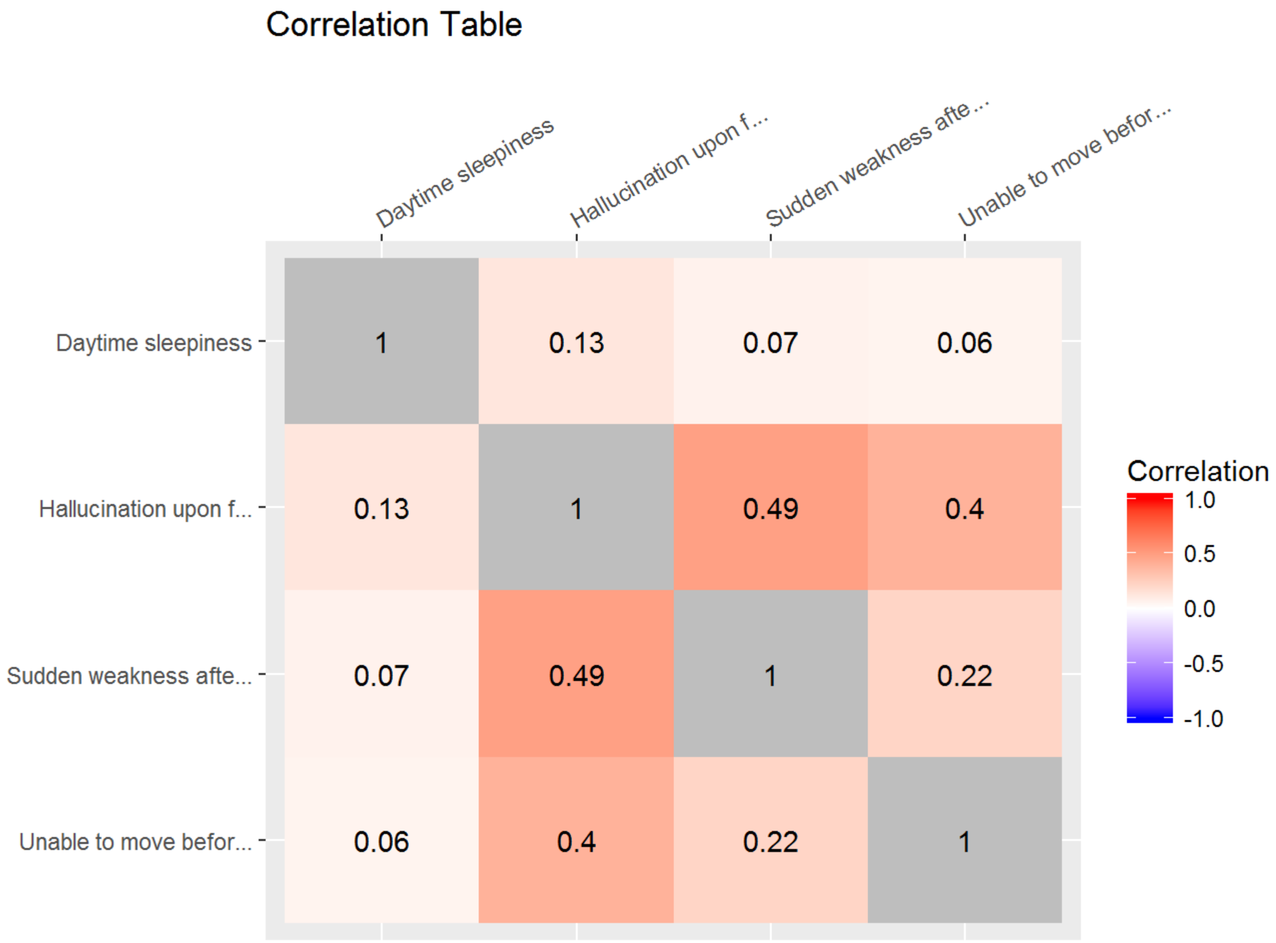

Sleep Medication Use

Value	Percent	Count	Male	Female
Yes	54.4	629	209	420
No	42.3	489	201	288
NA	3.4	39	18	21

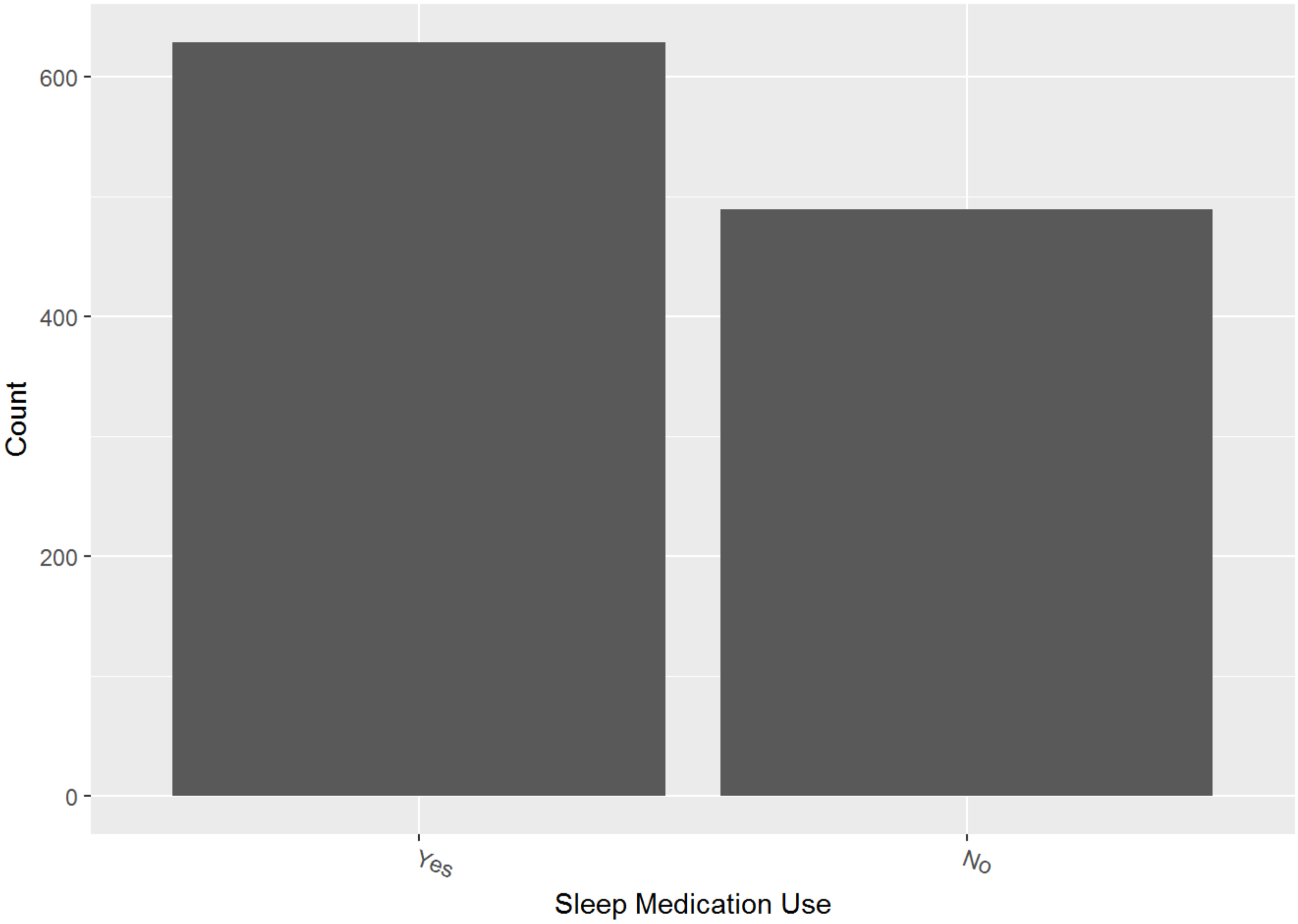

Family History of Circardian Rhythm Disorder

Value	Percent	Count	Male	Female
No	96.8	1120	414	706
Yes	3.2	37	14	23

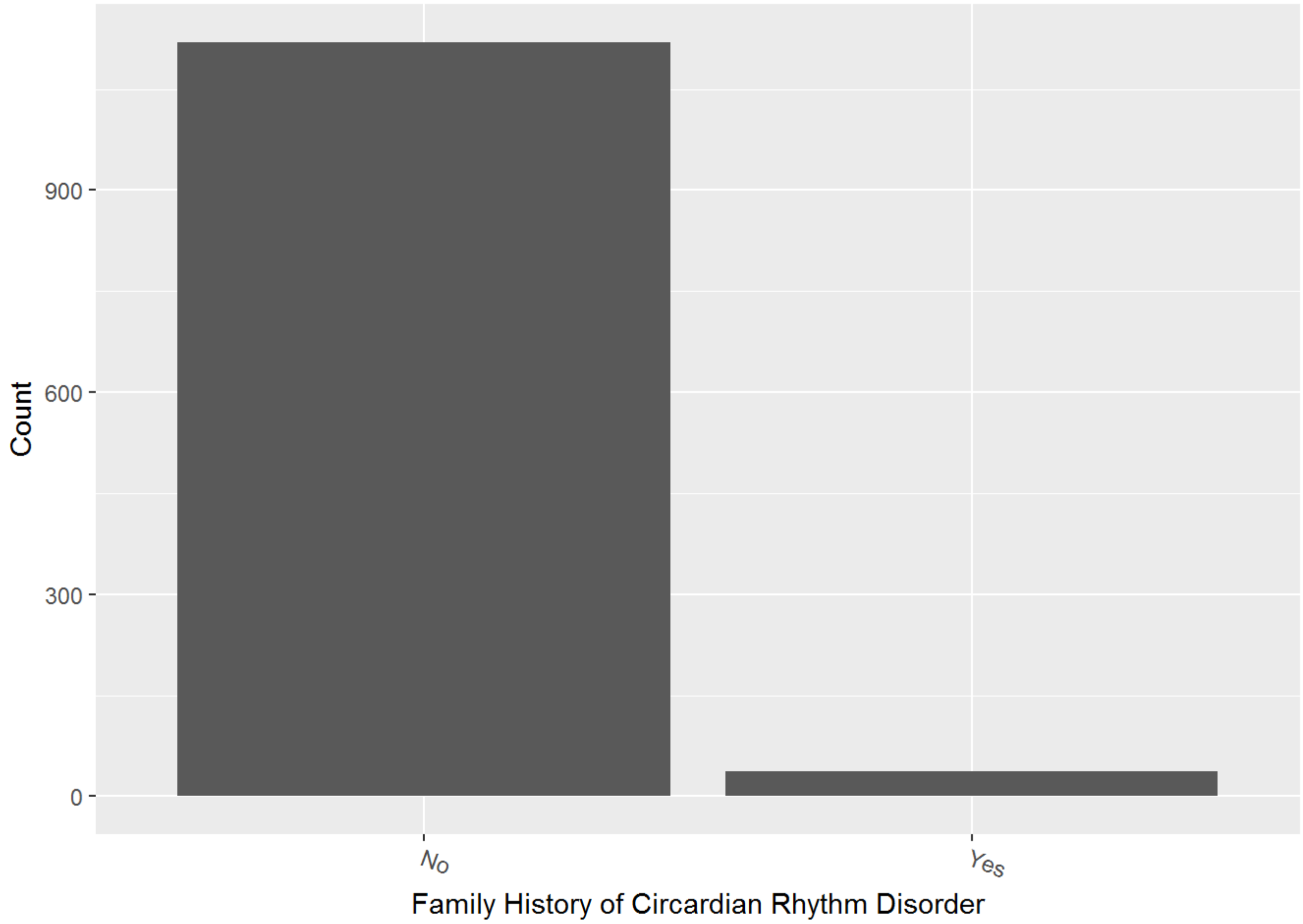

Family Members with History of Circardian Rhythm Disorder

Value	Percent	Count	Male	Female
MO	0.5	6	0	6
SIS	0.3	3	2	1
OTH	0.2	2	0	2
FA	0.1	1	1	0
MA	0.1	1	0	1
MGM	0.1	1	0	1
SN	0.1	1	1	0

Family History of Hypersomnia

Value	Percent	Count	Male	Female
No	96.6	1118	411	707
Yes	3.4	39	17	22

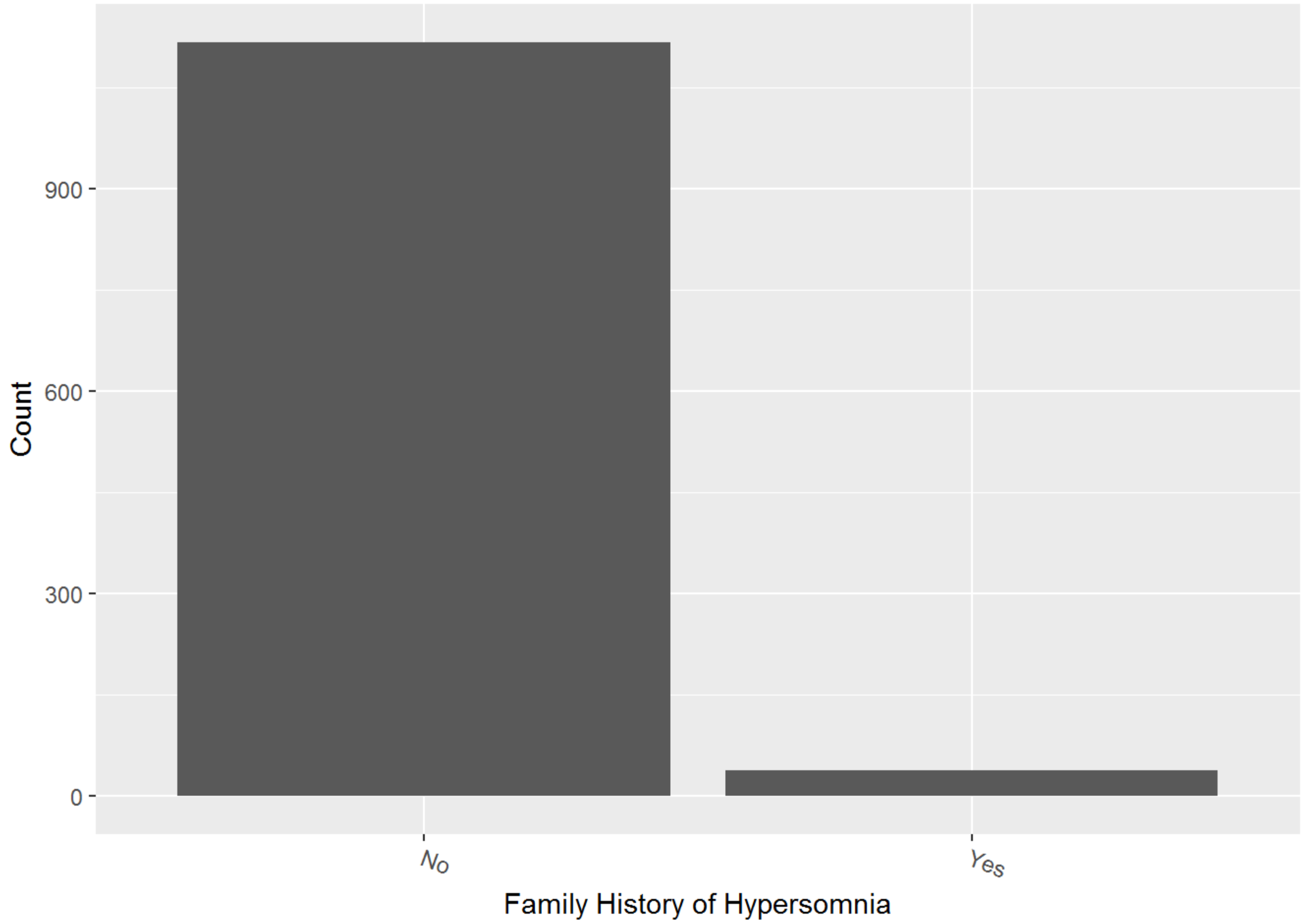

Family Members with History of Hypersomnia

Value	Percent	Count	Male	Female
MO	0.5	6	3	3
FA	0.3	4	3	1
SIS	0.3	4	1	3
BRO	0.1	1	1	0
MU	0.1	1	1	0

Family History of Insomnia

Value	Percent	Count	Male	Female
No	86.5	1001	381	620
Yes	13.5	156	47	109

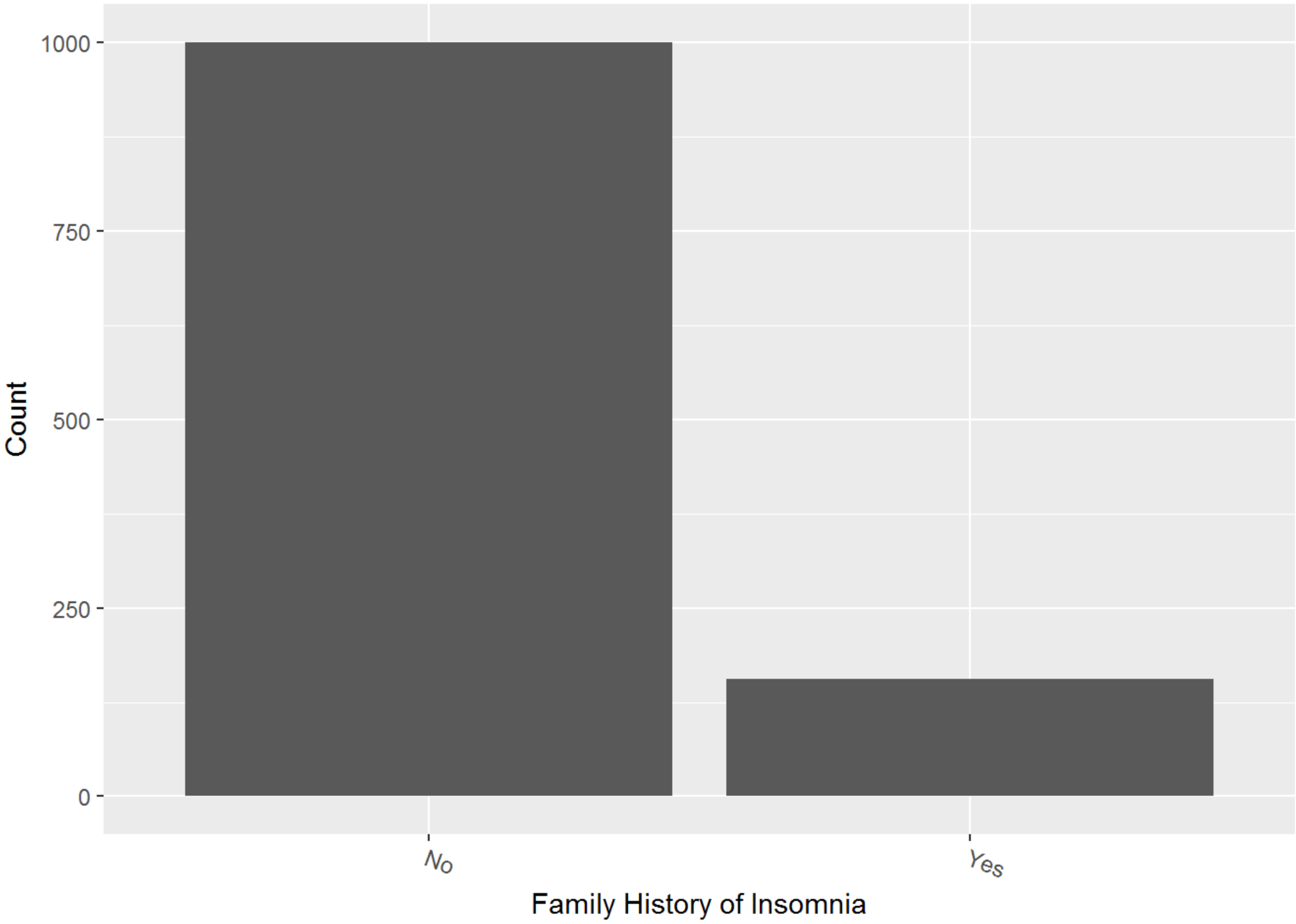

Family Members with History of Insomnia

Value	Percent	Count	Male	Female
MO	5.7	66	19	47
FA	2.2	26	7	19
SIS	2	23	4	19
BRO	1.4	16	7	9
DA	0.7	8	2	6
SN	0.7	8	0	8
MGM	0.5	6	2	4
PGF	0.2	2	0	2
PGM	0.2	2	1	1
MGF	0.1	1	0	1
MU	0.1	1	1	0
PC	0.1	1	1	0

Family History of Narcolepsy

Value	Percent	Count	Male	Female

No	96.7	1119	413	706
Yes	3.3	38	15	23

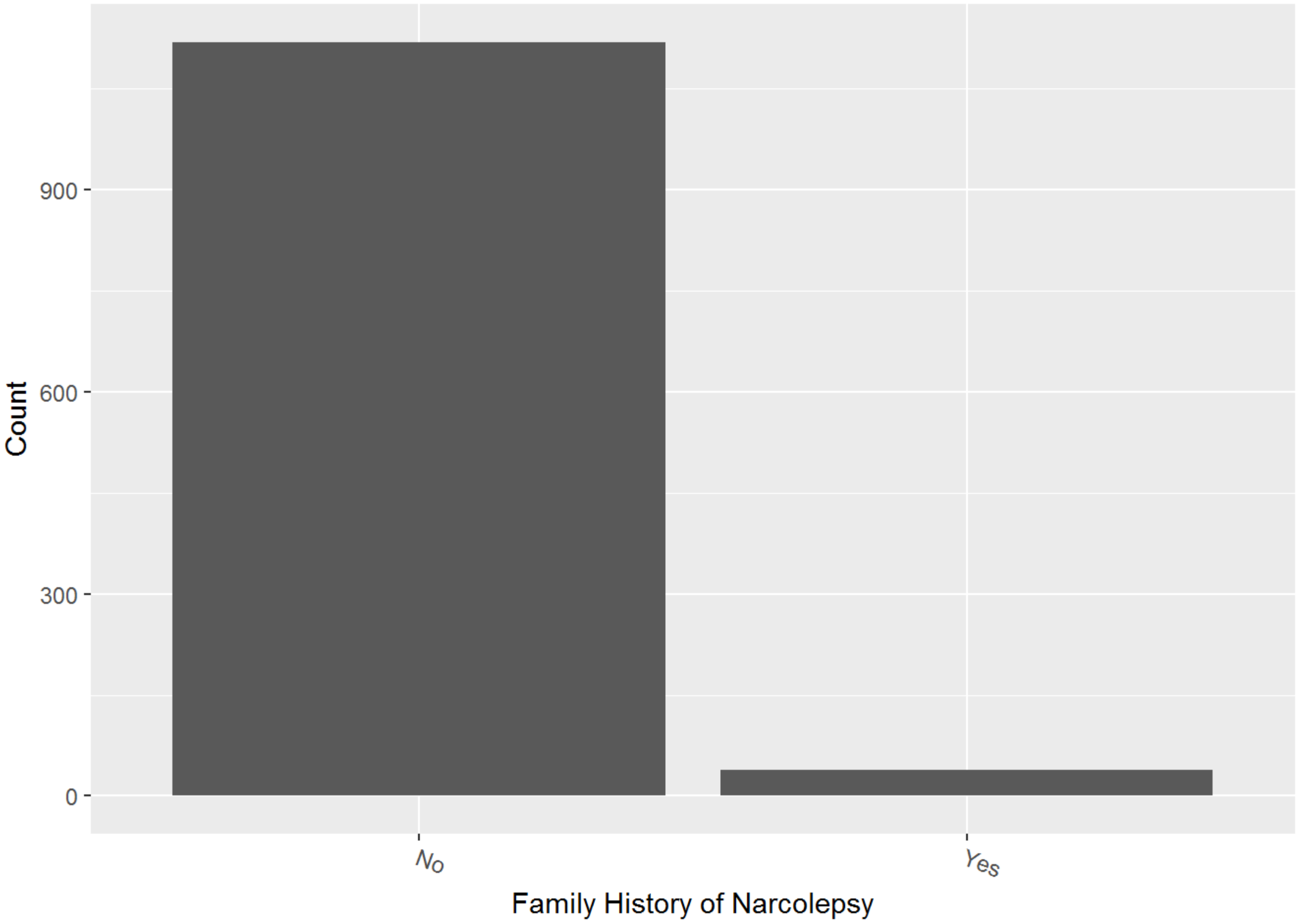

Family Members with History of Narcolepsy

Value	Percent	Count	Male	Female
BRO	0.3	3	2	1
FA	0.3	3	1	2
MGF	0.2	2	0	2
SIS	0.2	2	1	1
DA	0.1	1	0	1
MO	0.1	1	0	1
PU	0.1	1	1	0

Family History of Parasomnia

Value	Percent	Count	Male	Female
No	95.5	1105	410	695
Yes	4.5	52	18	34

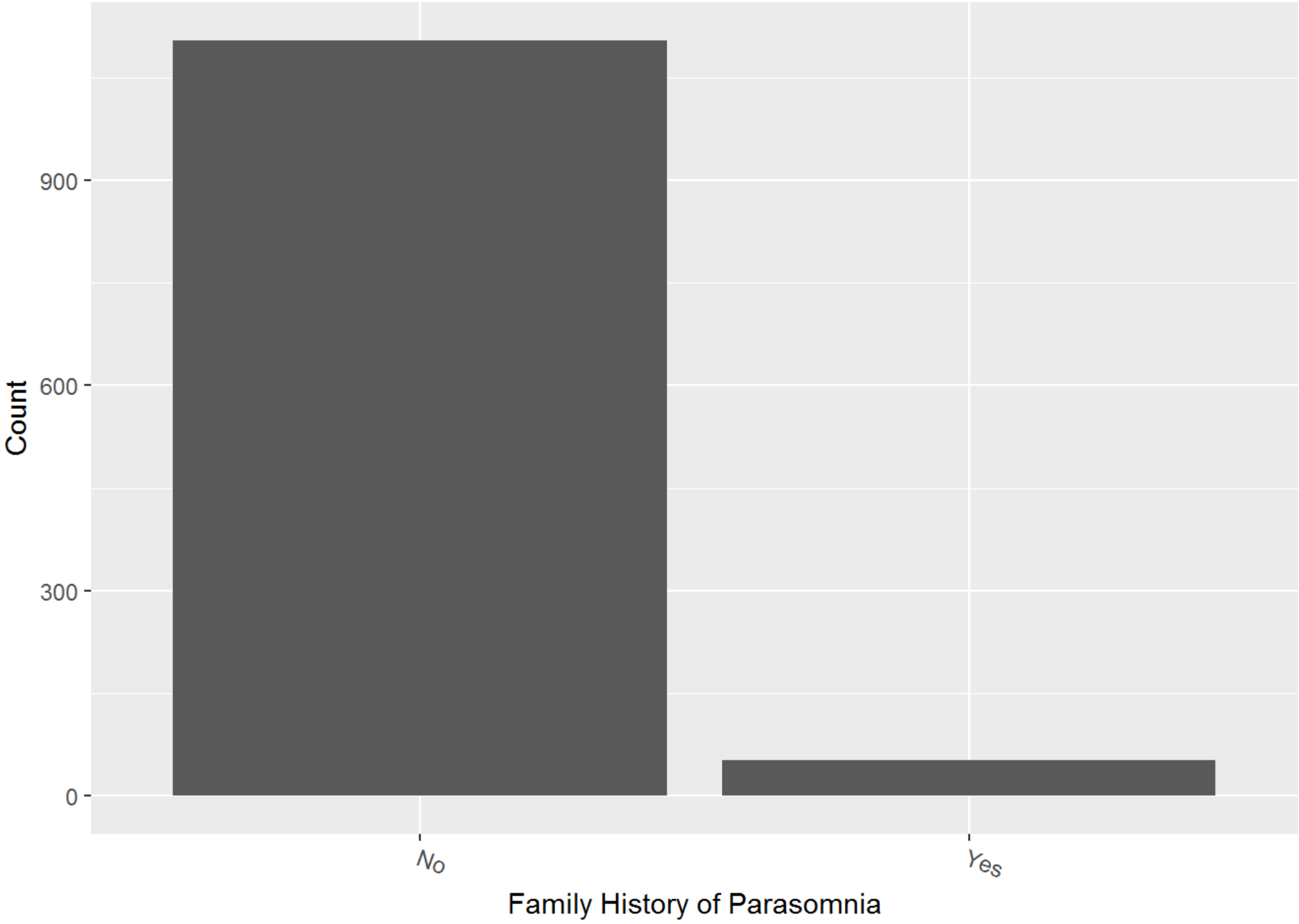

Family Members with History of Parasomnia

Value	Percent	Count	Male	Female
SIS	0.7	8	3	5
MO	0.5	6	0	6
BRO	0.3	4	0	4
FA	0.3	3	1	2
SN	0.3	3	1	2
DA	0.1	1	1	0
MA	0.1	1	1	0
MC	0.1	1	1	0
OTH	0.1	1	0	1

Family History of Parkinsons

Value	Percent	Count	Male	Female
No	91.7	1061	395	666
Yes	8.3	96	33	63

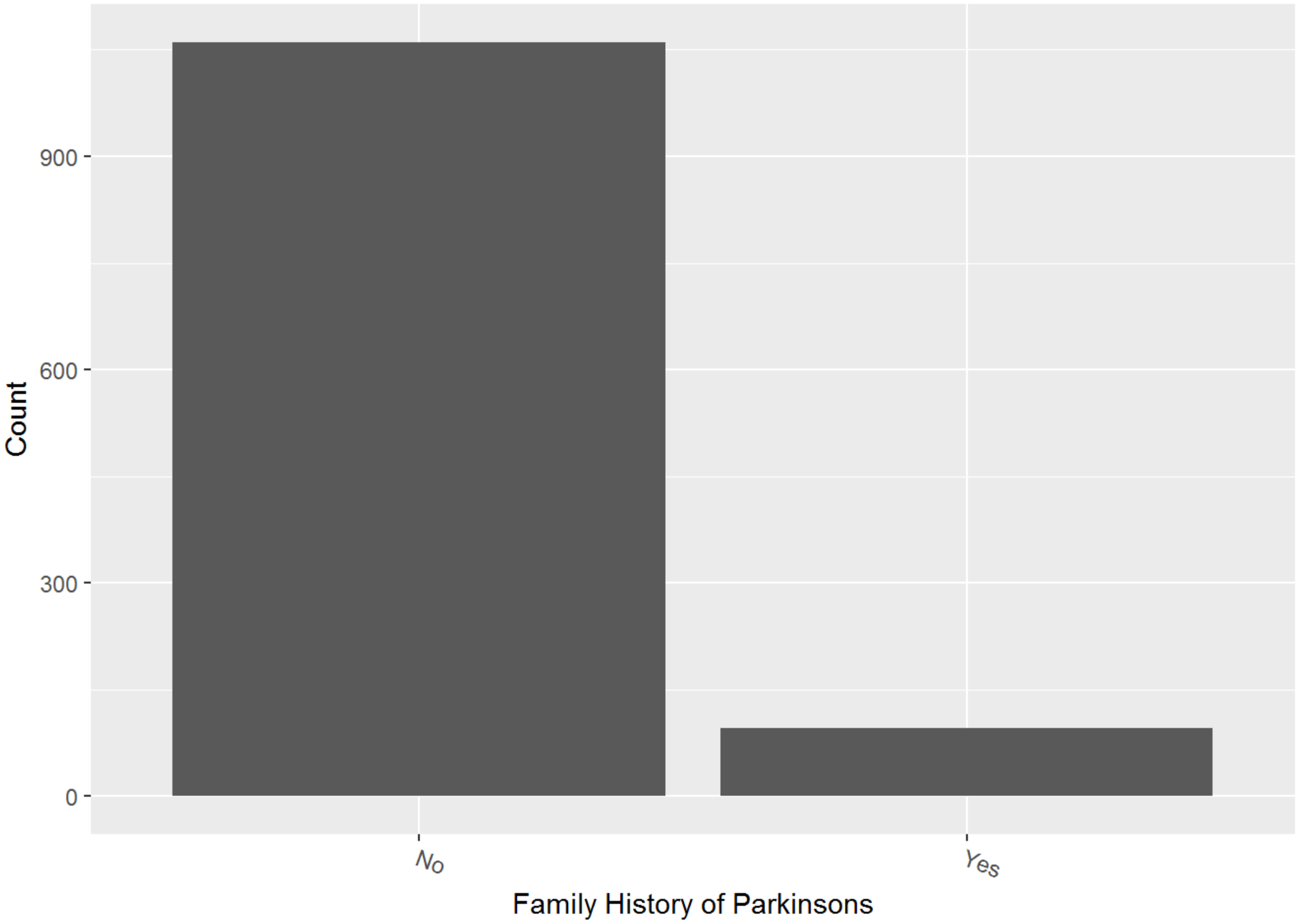

Family Members with History of Parkinsons

Value	Percent	Count	Male	Female
FA	1.8	21	7	14
MGM	0.9	10	3	7
MO	0.7	8	2	6
PGF	0.7	8	3	5
PGM	0.5	6	1	5
BRO	0.4	5	3	2
PU	0.4	5	3	2
MA	0.3	4	1	3
MC	0.3	3	1	2
MU	0.3	4	0	4
SIS	0.3	3	0	3
PC	0.2	2	0	2
MGF	0.1	1	0	1
PA	0.1	1	0	1
SN	0.1	1	0	1

Family History of REM Sleep Disorder

Value	Percent	Count	Male	Female
No	97.3	1126	416	710
Yes	2.7	31	12	19

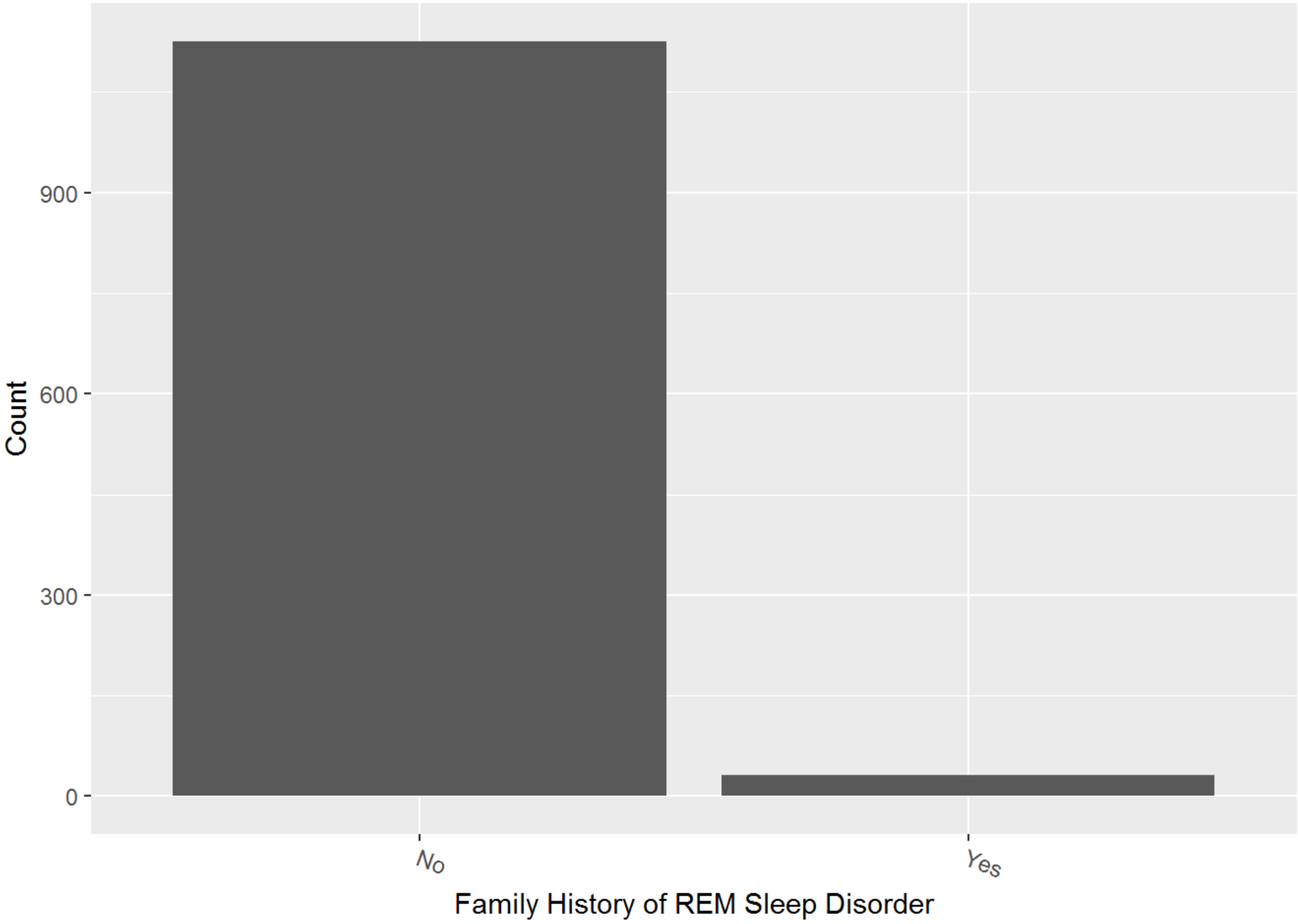

Family Members with History of REM Sleep Disorder

Value	Percent	Count	Male	Female
MO	0.3	3	1	2
FA	0.2	2	1	1
BRO	0.1	1	0	1
SIS	0.1	1	0	1

Family History of RLS

Value	Percent	Count	Male	Female
No	72.3	836	330	506
Yes	27.7	321	98	223

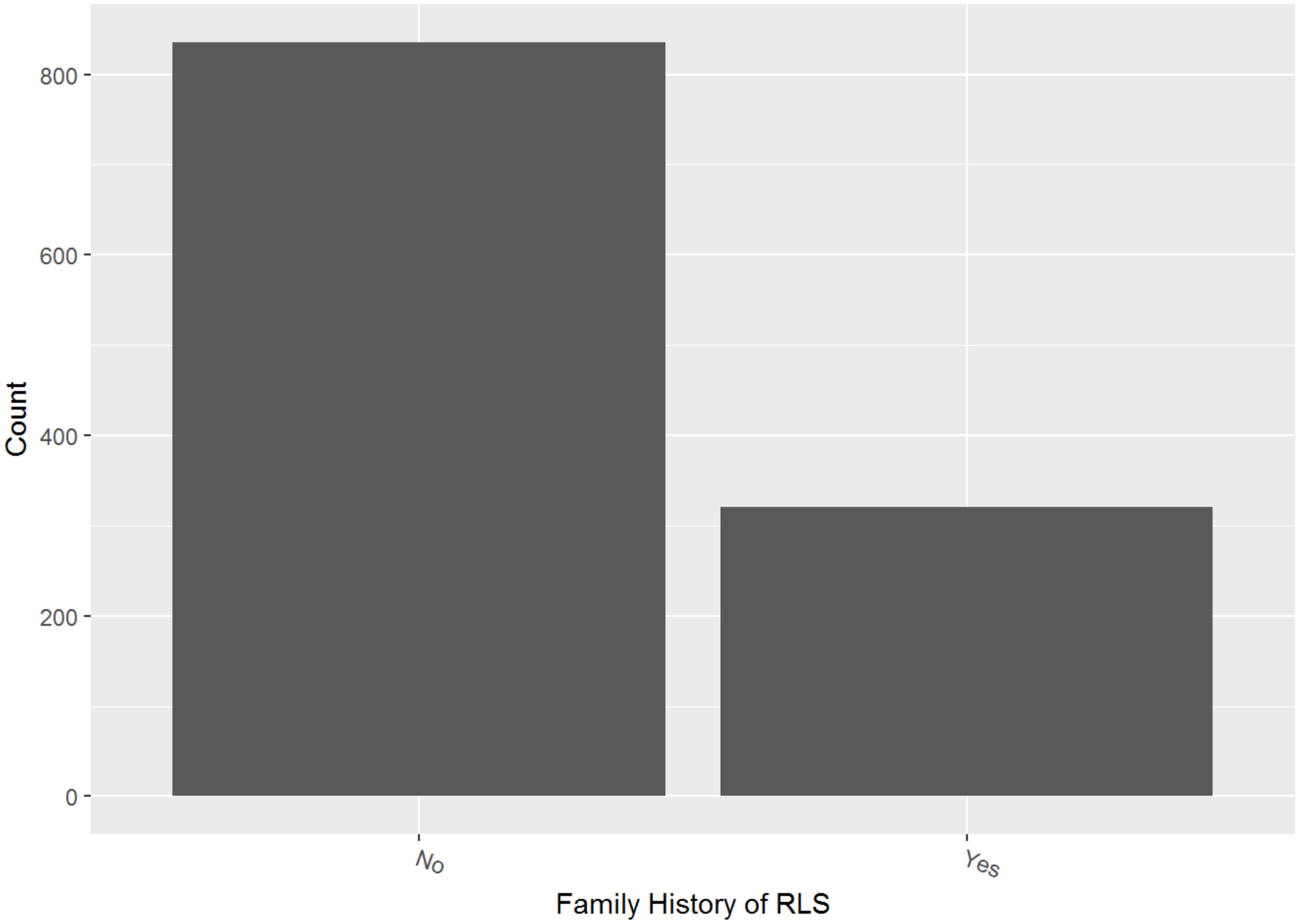

Family Members with History of RLS

Value	Percent	Count	Male	Female
MO	10.5	122	30	92
FA	7.4	86	34	52
SIS	5.6	65	17	48
BRO	3.3	38	11	27
DA	1.9	22	3	19
SN	1.6	19	3	16
MGM	1.5	17	4	13
MA	0.7	8	2	6
MGF	0.6	7	4	3
PGM	0.6	7	3	4
MU	0.4	5	3	2
OTH	0.3	3	1	2
MC	0.2	2	0	2
PC	0.2	2	2	0
PGF	0.2	2	1	1
PU	0.1	1	1	0

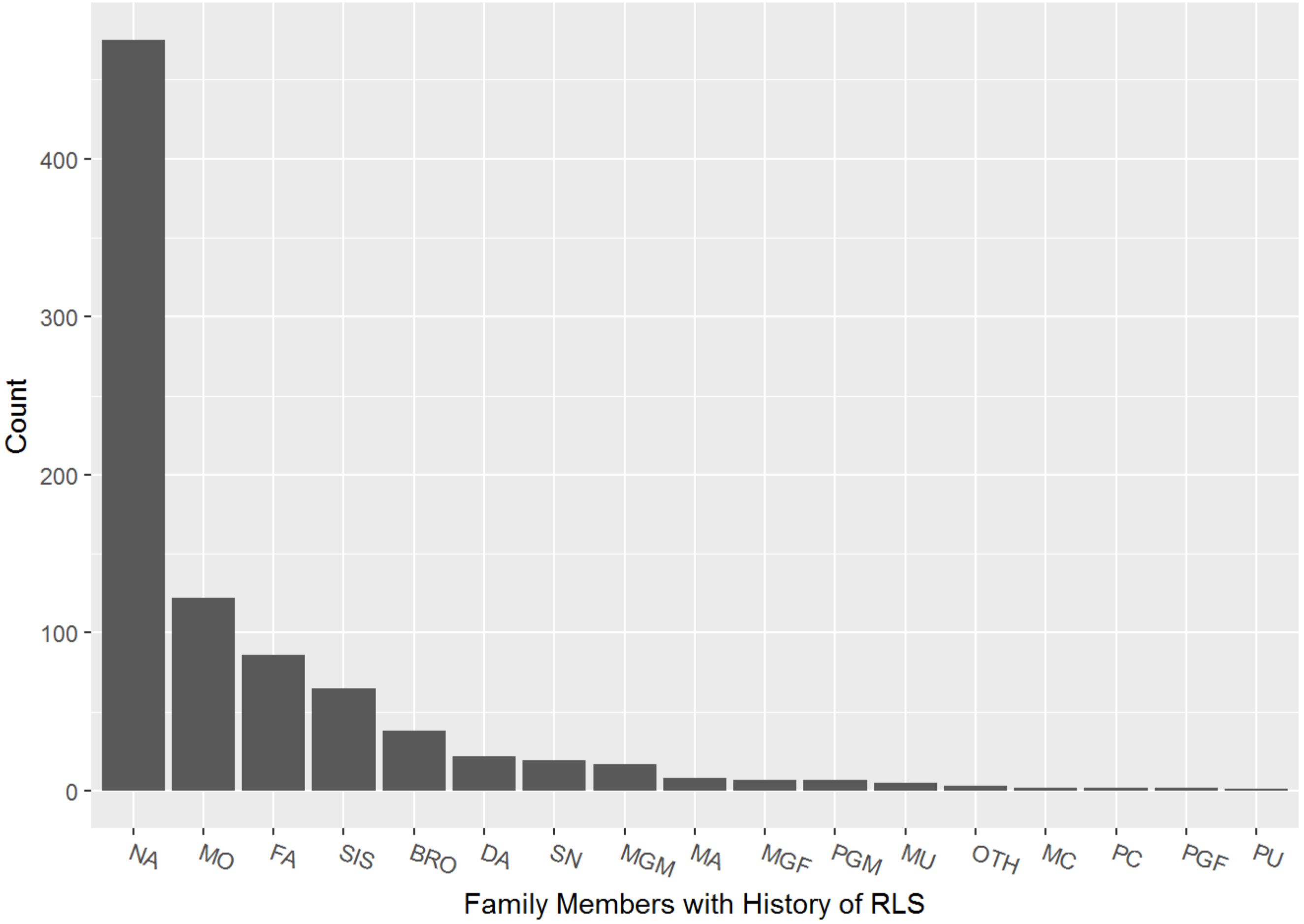

Correlation Table

	BRO	DA	FA	MA	MC	MGF	MGM	MO	MU	NA	OTH	PC	PGF	PGM	PU	SIS	SN
BRO	1	0.01	0.08	0.04	-0.01	0.05	0.06	0.11	0.06	-0.03	-0.01	0.11	-0.01	-0.01	0.16	0.12	0.05
DA	0.01	1	0.03	-0.01	-0.01	-0.01	0.04	0.01	-0.01	-0.02	-0.01	-0.01	-0.01	0.07	0	0.02	0.13
FA	0.08	0.03	1	-0.02	-0.01	0.02	0.02	-0.01	-0.02	-0.04	-0.01	0.07	-0.01	0.19	0.1	0.05	0.07
MA	0.04	-0.01	-0.02	1	0.25	-0.01	0.16	0.18	0.31	-0.01	0	0	0	-0.01	0	0.21	-0.01
MC	-0.01	-0.01	-0.01	0.25	1	0	-0.01	0.05	0	-0.01	0	0	0	0	0	0.08	0.16
MGF	0.05	-0.01	0.02	-0.01	0	1	-0.01	0.08	0.16	-0.01	0	0	0	-0.01	0	0.03	-0.01
MGM	0.06	0.04	0.02	0.16	-0.01	-0.01	1	0.12	0.1	-0.02	-0.01	-0.01	-0.01	-0.01	0	0	-0.02
MO	0.11	0.01	-0.01	0.18	0.05	0.08	0.12	1	0.06	-0.05	-0.02	0.05	-0.01	0.08	0.09	0.21	0.07
MU	0.06	-0.01	-0.02	0.31	0	0.16	0.1	0.06	1	-0.01	0	0	0	-0.01	0	0.04	-0.01
NA	-0.03	-0.02	-0.04	-0.01	-0.01	-0.01	-0.02	-0.05	-0.01	1	-0.01	-0.01	-0.01	-0.01	0	-0.04	-0.02
OTH	-0.01	-0.01	-0.01	0	0	0	-0.01	-0.02	0	-0.01	1	0	0	0	0	-0.01	-0.01
PC	0.11	-0.01	0.07	0	0	0	-0.01	0.05	0	-0.01	0	1	0	0	0.71	0.08	-0.01
PGF	-0.01	-0.01	-0.01	0	0	0	-0.01	-0.01	0	-0.01	0	0	1	0	0	-0.01	0.16
PGM	-0.01	0.07	0.19	-0.01	0	-0.01	-0.01	0.08	-0.01	-0.01	0	0	0	1	0	0.08	-0.01
PU	0.16	0	0.1	0	0	0	0	0.09	0	0	0	0.71	0	0	1	0.12	0
SIS	0.12	0.02	0.05	0.21	0.08	0.03	0	0.21	0.04	-0.04	-0.01	0.08	-0.01	0.08	0.12	1	0.03
SN	0.05	0.13	0.07	-0.01	0.16	-0.01	-0.02	0.07	-0.01	-0.02	-0.01	-0.01	0.16	-0.01	0	0.03	1

Correlation: 1.0, 0.5, 0.0, -0.5, -1.0

Family History of Sleep Apnea

Value	Percent	Count	Male	Female
No	71.7	829	324	505
Yes	28.3	328	104	224

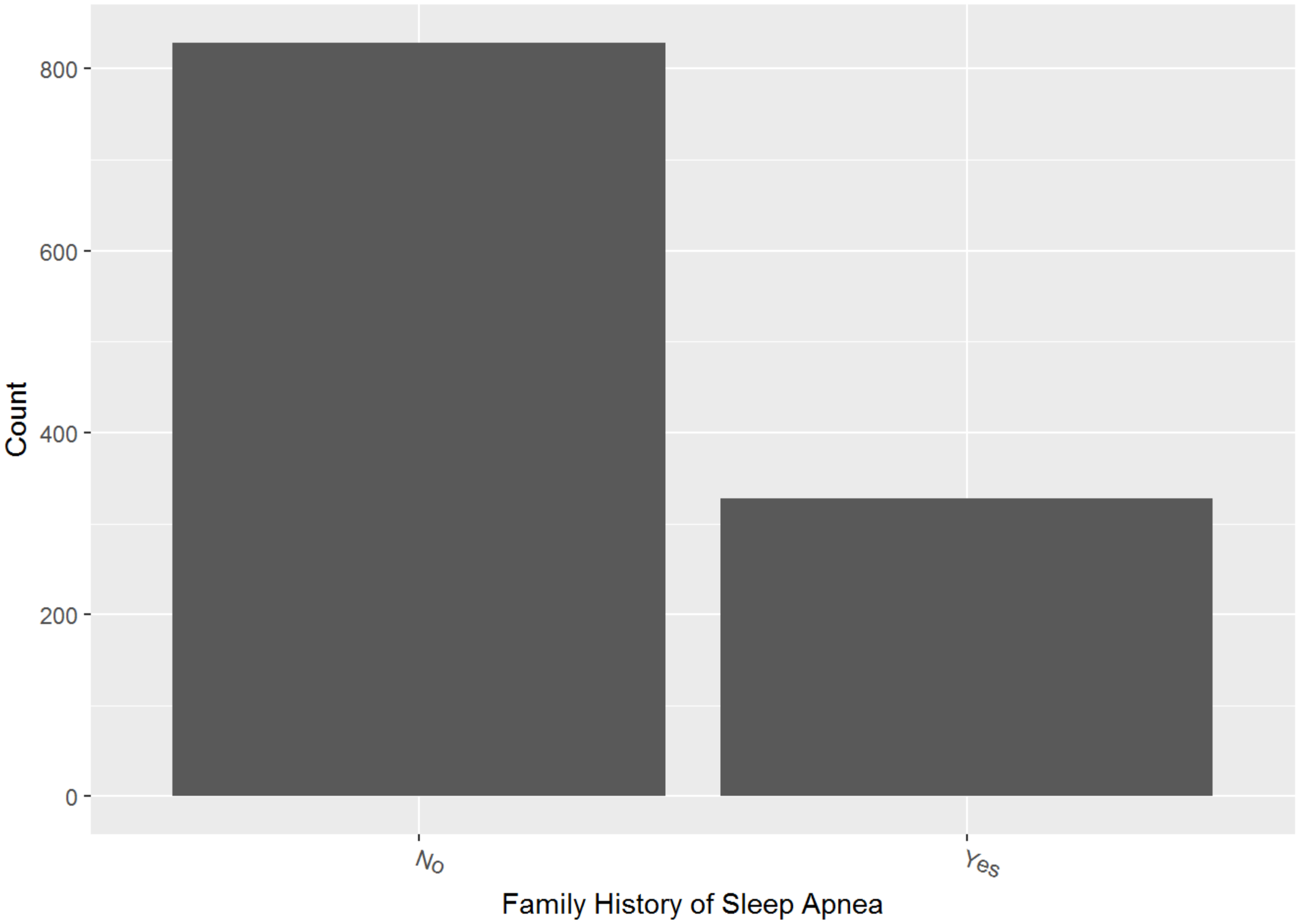

Family Members with History of Sleep Apnea

Value	Percent	Count	Male	Female
FA	9.8	113	38	75
BRO	7.5	87	29	58
SIS	4.2	49	11	38
MO	4	46	15	31
SN	2.2	25	8	17
DA	1.3	15	1	14
MU	1.2	14	6	8
MGF	0.8	9	2	7
MC	0.6	7	2	5

MA	0.5	6	1	5
MGM	0.4	5	0	5
PU	0.4	5	3	2
OTH	0.3	3	1	2
PA	0.2	2	0	2
PGF	0.2	2	1	1
PGM	0.2	2	0	2
PC	0.1	1	0	1

Family History of Sleep Movement Disorder

Value	Percent	Count	Male	Female
No	97.1	1124	415	709
Yes	2.9	33	13	20

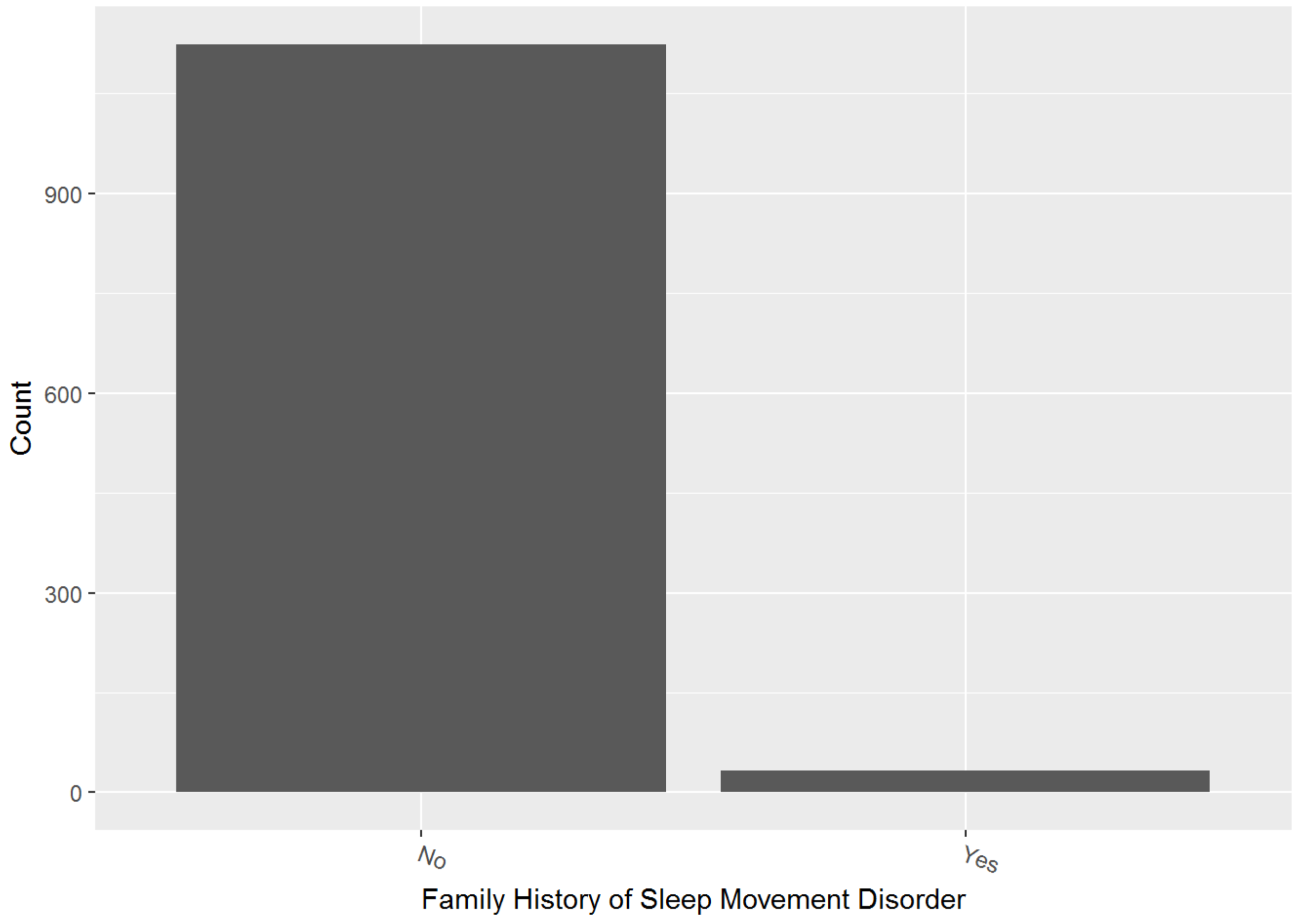

Family Members with History of Sleep Movement Disorder

Value	Percent	Count	Male	Female
FA	0.3	3	3	0

MO	0.3	3	0	3
DA	0.1	1	0	1
MGM	0.1	1	0	1
SN	0.1	1	0	1

Family History of Snoring

Value	Percent	Count	Male	Female
No	69.9	809	293	516
Yes	30.1	348	135	213

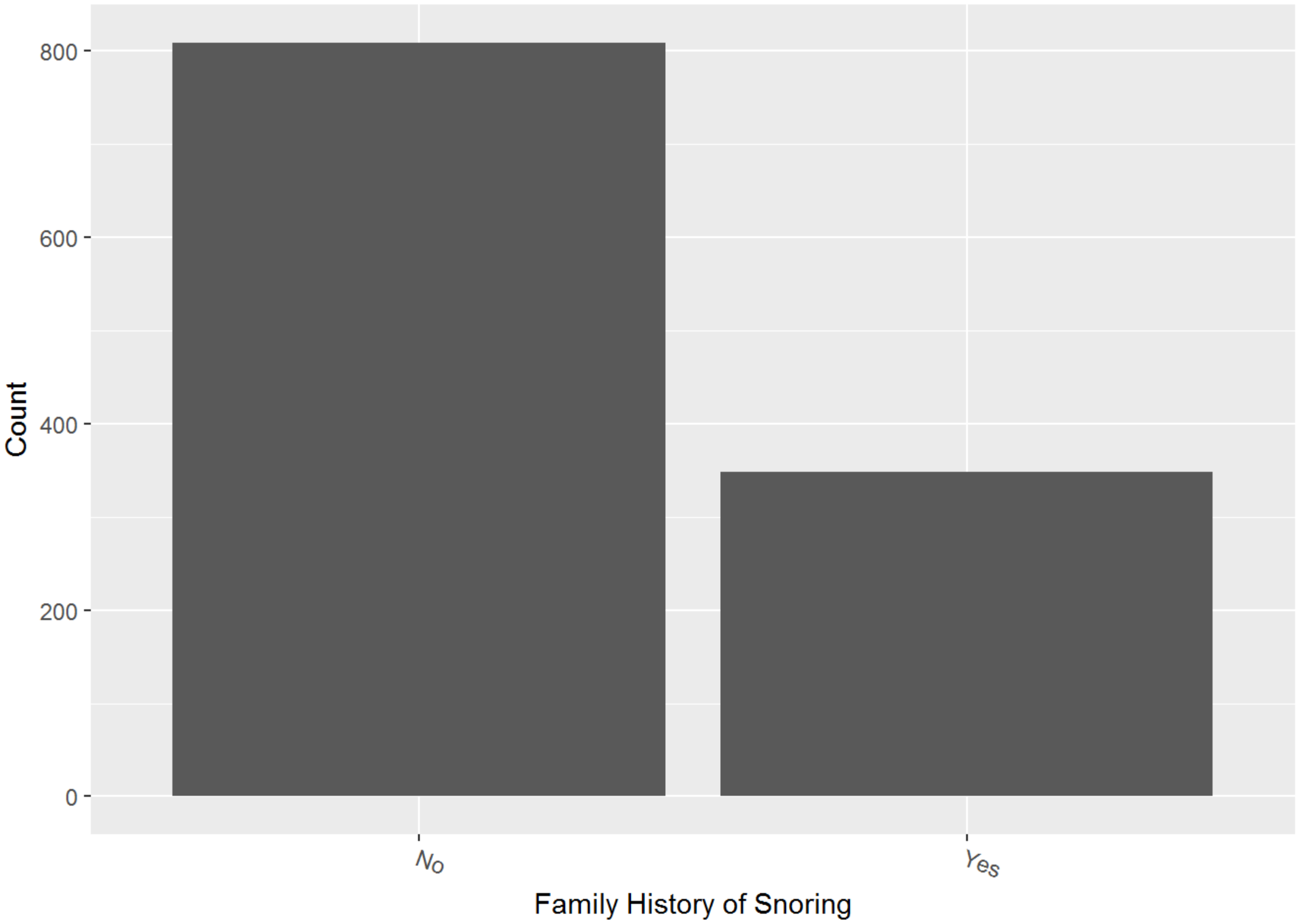

Family Members with History of Snoring

Value	Percent	Count	Male	Female
FA	18.7	216	87	129
MO	10.3	119	39	80
BRO	3.3	38	20	18
SIS	2.5	29	7	22
SN	1	11	4	7

MGF	0.6	7	3	4
MGM	0.5	6	5	1
DA	0.4	5	1	4
MA	0.3	3	3	0
PGF	0.3	4	2	2
MC	0.2	2	1	1
PGM	0.2	2	1	1
PU	0.2	2	1	1
MU	0.1	1	0	1
PC	0.1	1	1	0
