# Supplementary material for: Quality improvement and practice-based research in sleep medicine using structured clinical documentation in the electronic medical record
Source: Sleep Sci Pract. Author manuscript; Available in PMC 2020 May 11. (PMC7213673; doi:10.1186/s41606-019-0038-2)
Supplement: Additional file 3 — Score tests distributions, correlations, and principal component analysis of patients at initial visit. [file NIHMS1574724-supplement-Additional_file_3.pdf]

Sleep Disorders

Score Tests, Correlations, and Principal Component Analysis

2019-02-01

- Variable Descriptives
- All Pairwise Correlations
- Principal Components, full sample

Total Patients = 1157

All DodoNA eligible patients regardless of enrollment

Encounters restricted to the following visit types: Initial Visit

---

Variable Descriptives

ESS Score

Min	Median	Mean	Max	St.Dev	No.Data
0	7	8.245	24	5.399	60

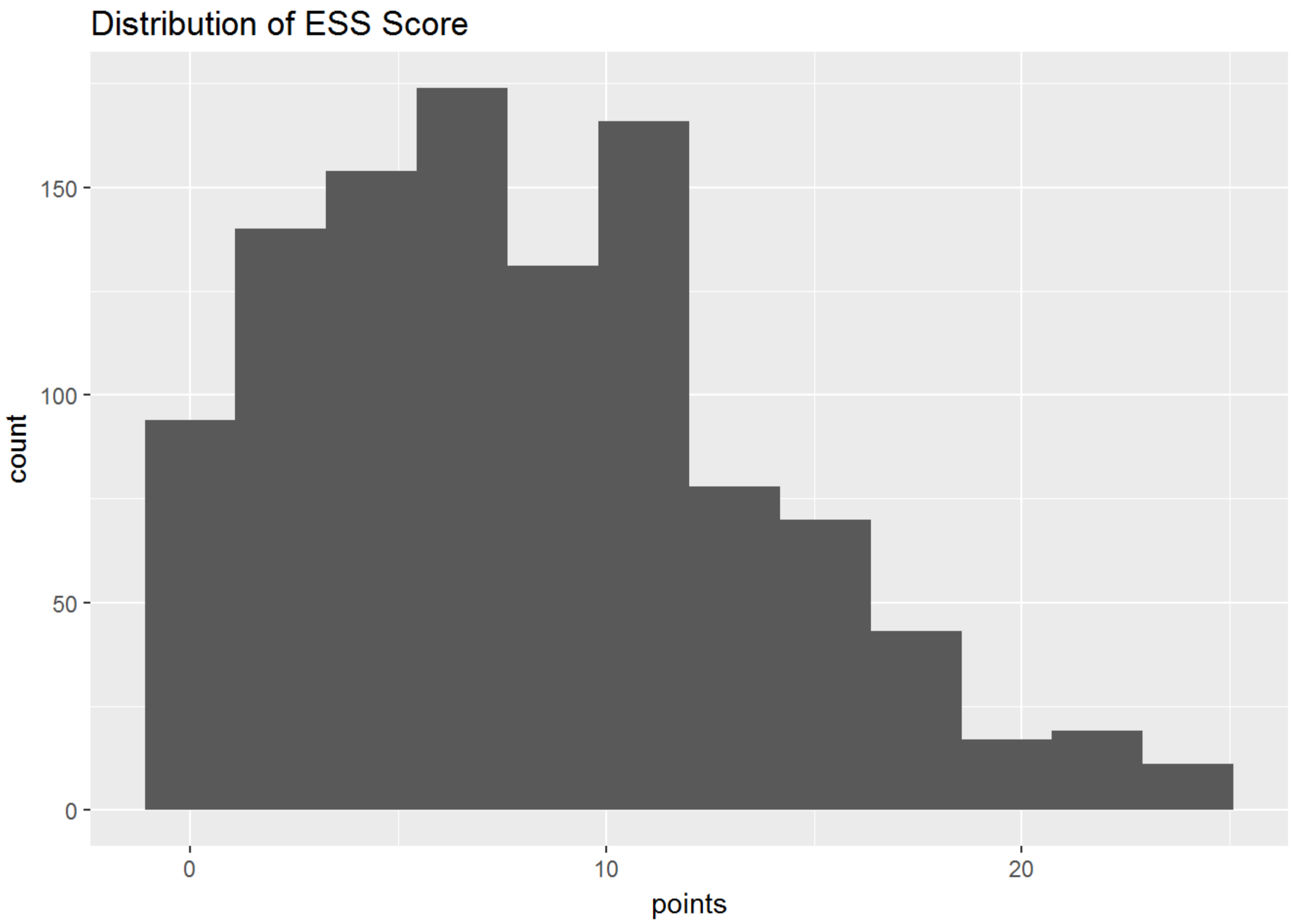
Distribution of ESS Score
count
150
100
50
0
0
10
20
points

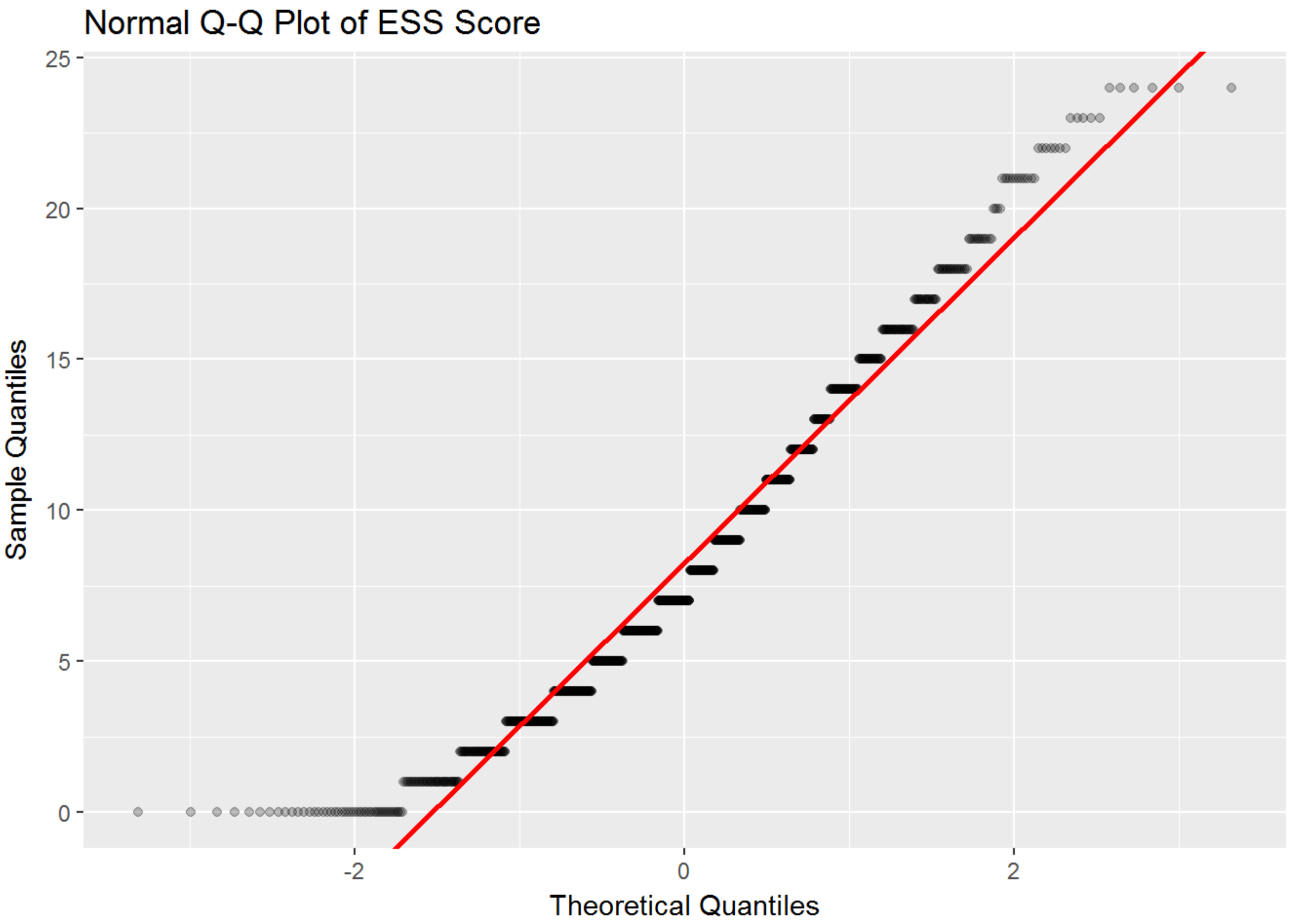
Normal Q-Q Plot of ESS Score
Sample Quantiles
25
20
15
10
5
0
-2
0
2
Theoretical Quantiles

CESD Score

Min	Median	Mean	Max	St.Dev	No.Data
0	12	14.24	55	10.54	94

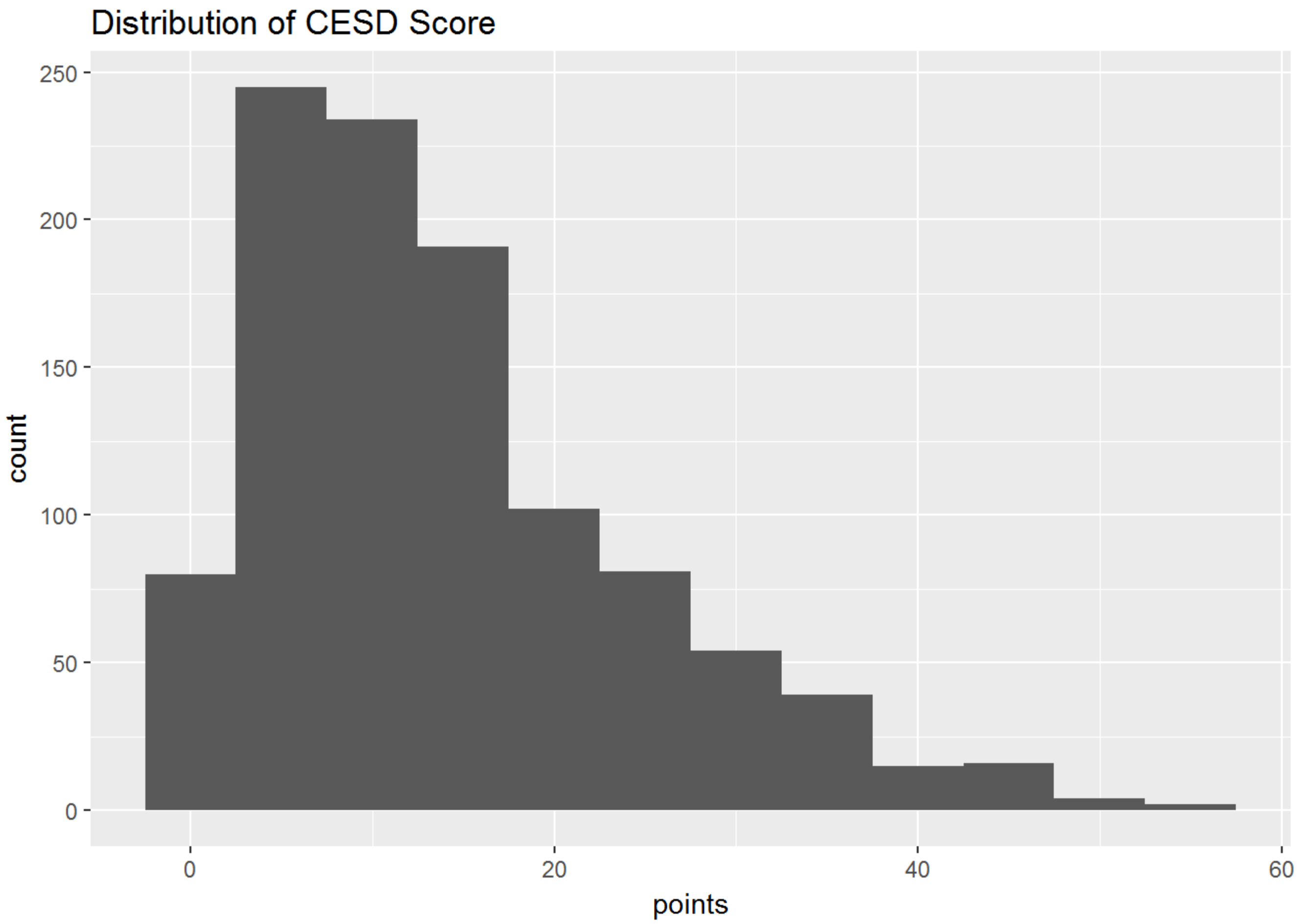

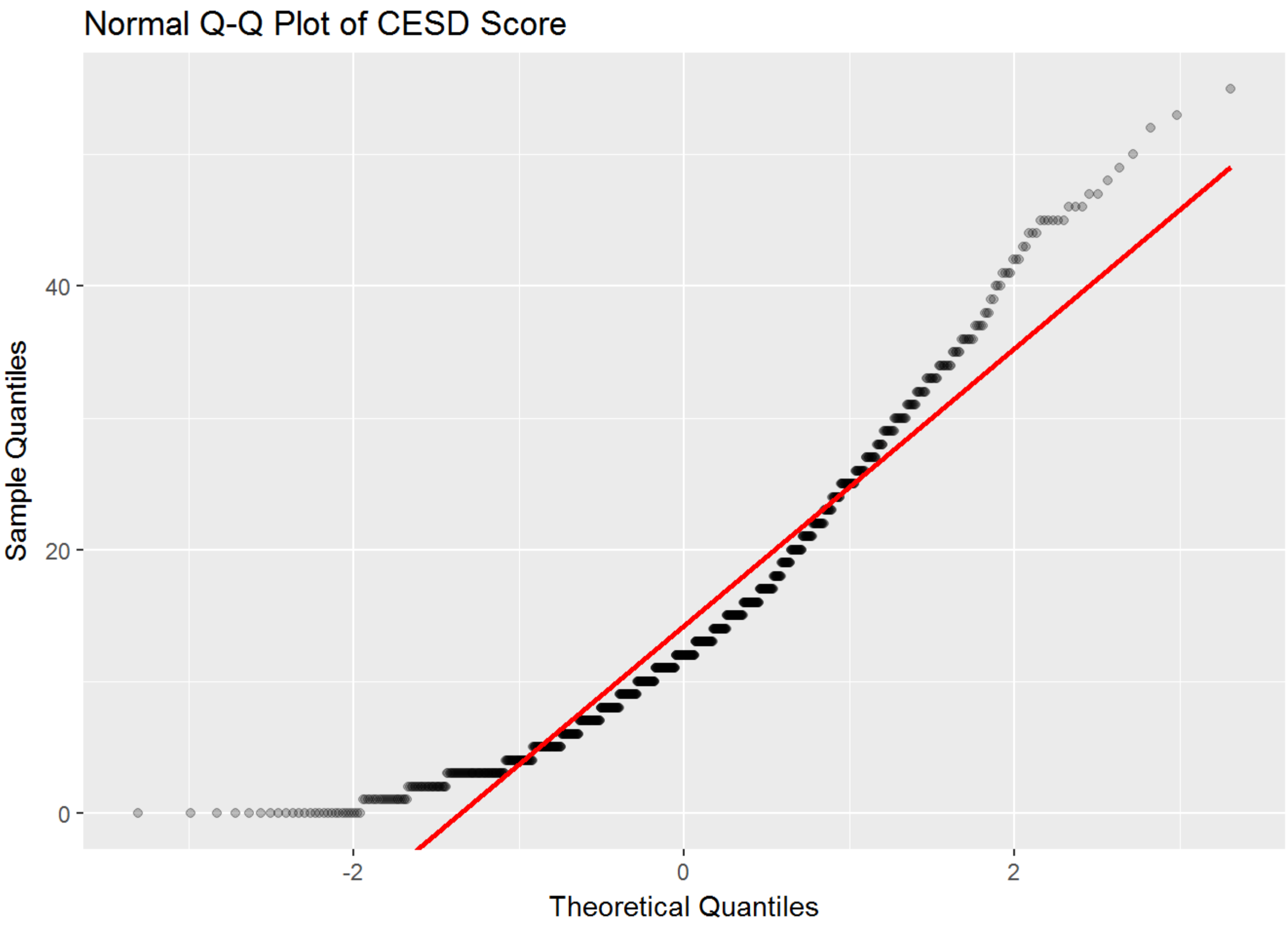

GAD7 Score

Min	Median	Mean	Max	St.Dev	No.Data
0	5	6.028	21	5.505	80

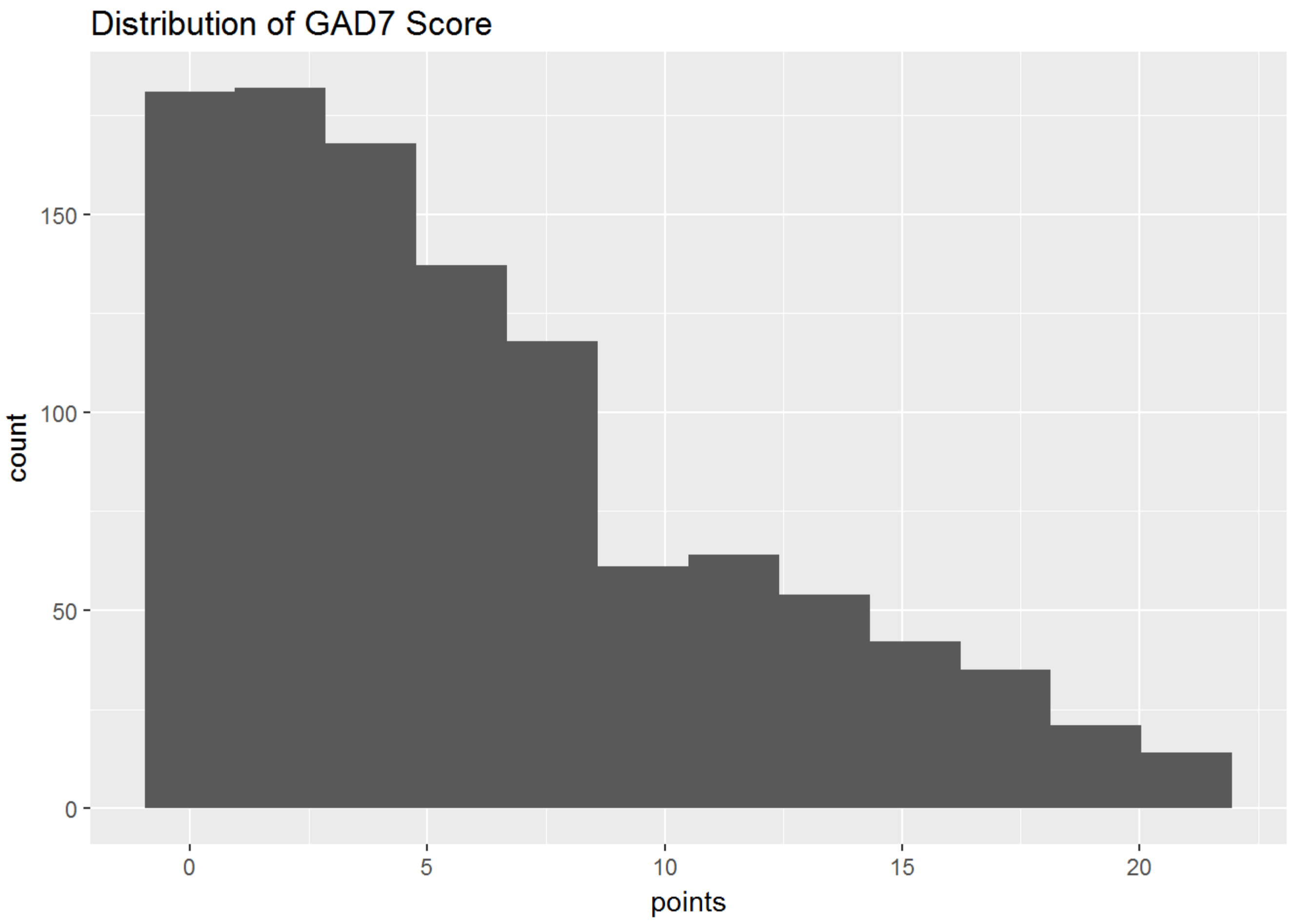
Distribution of GAD7 Score
count
150
100
50
0
0
5
10
15
20
points

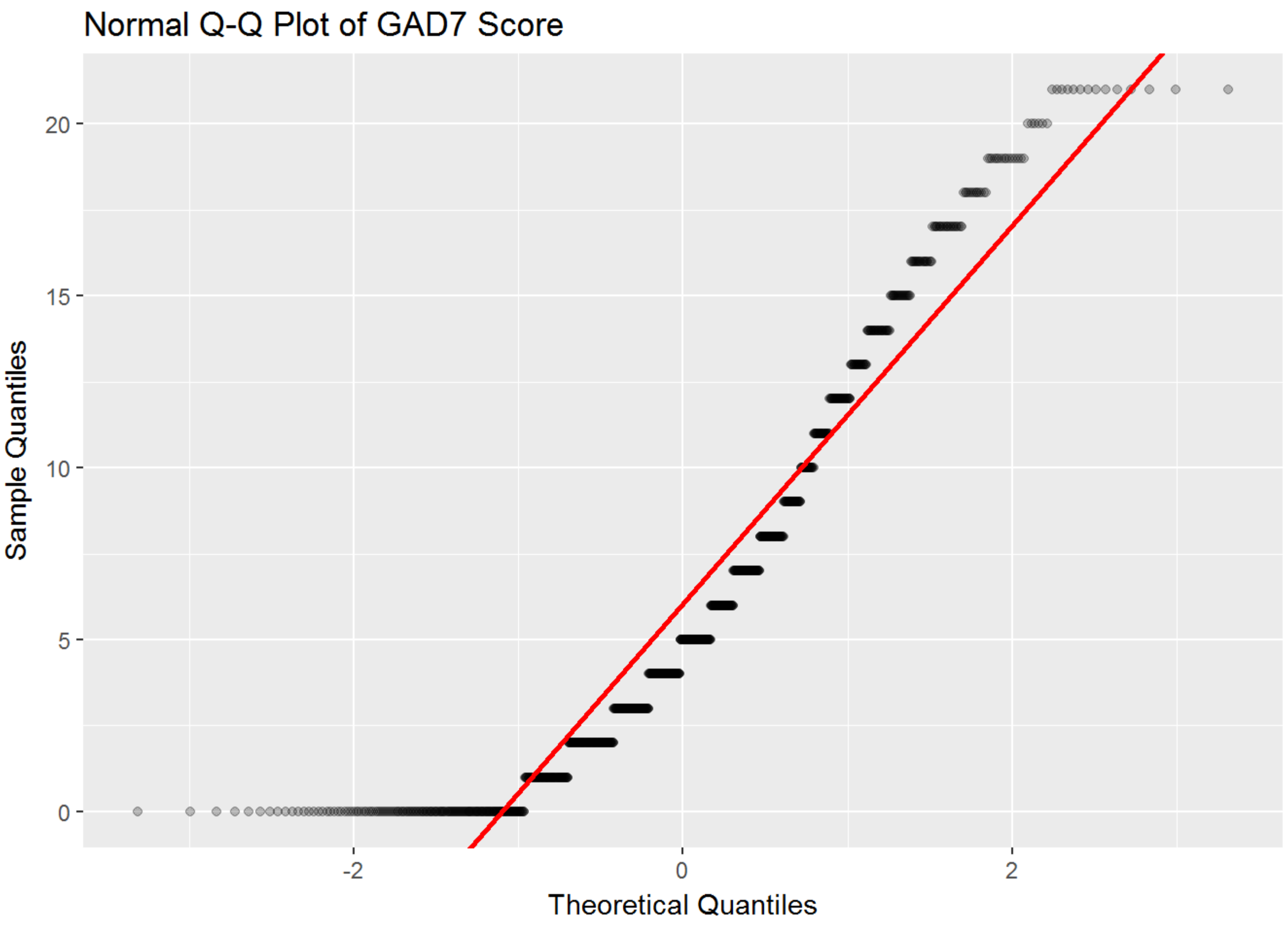
Normal Q-Q Plot of GAD7 Score
Sample Quantiles
20
15
10
5
0
-2
0
2
Theoretical Quantiles

ISI Score

Min	Median	Mean	Max	St.Dev	No.Data
0	15	14.95	28	6.425	69

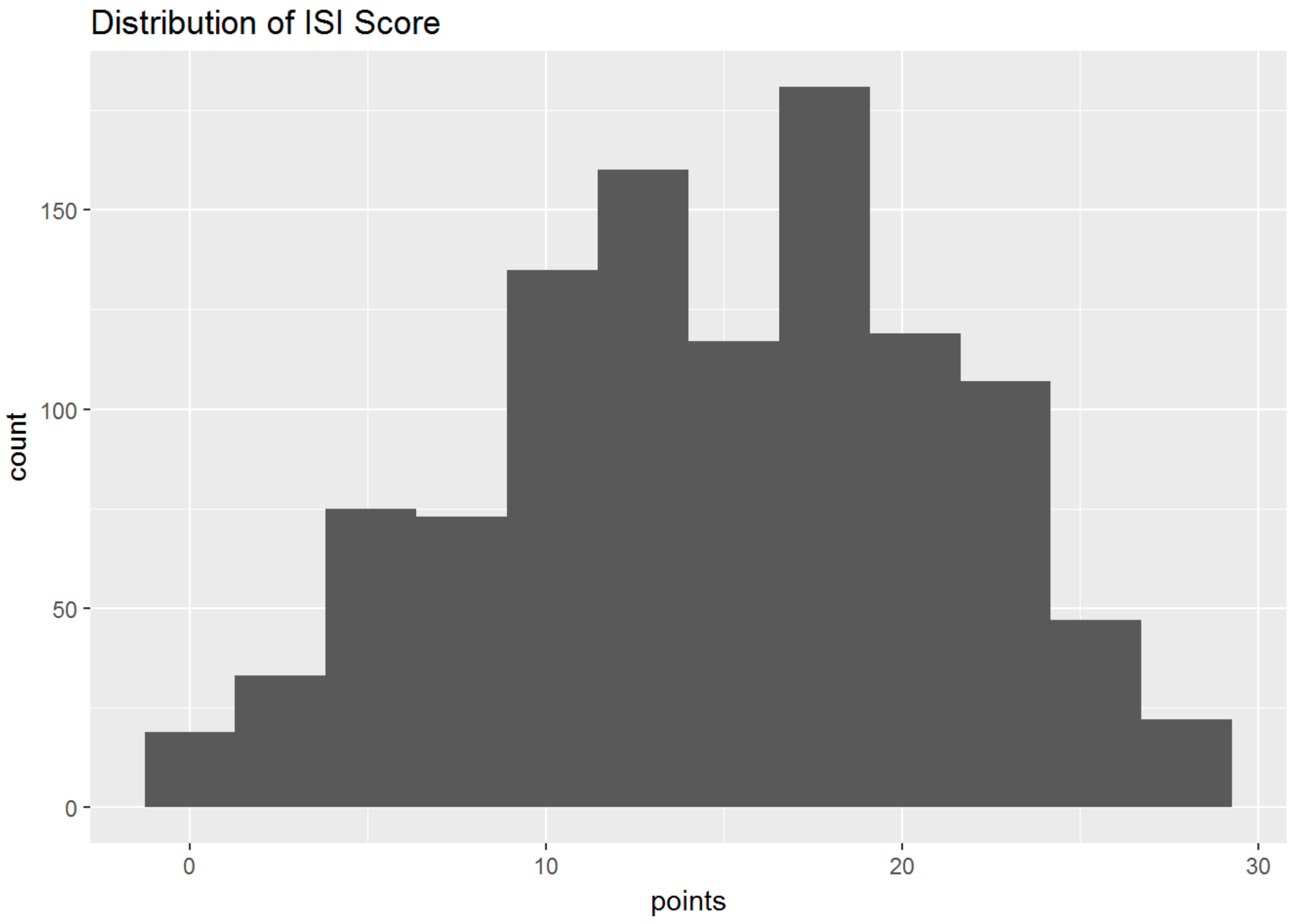

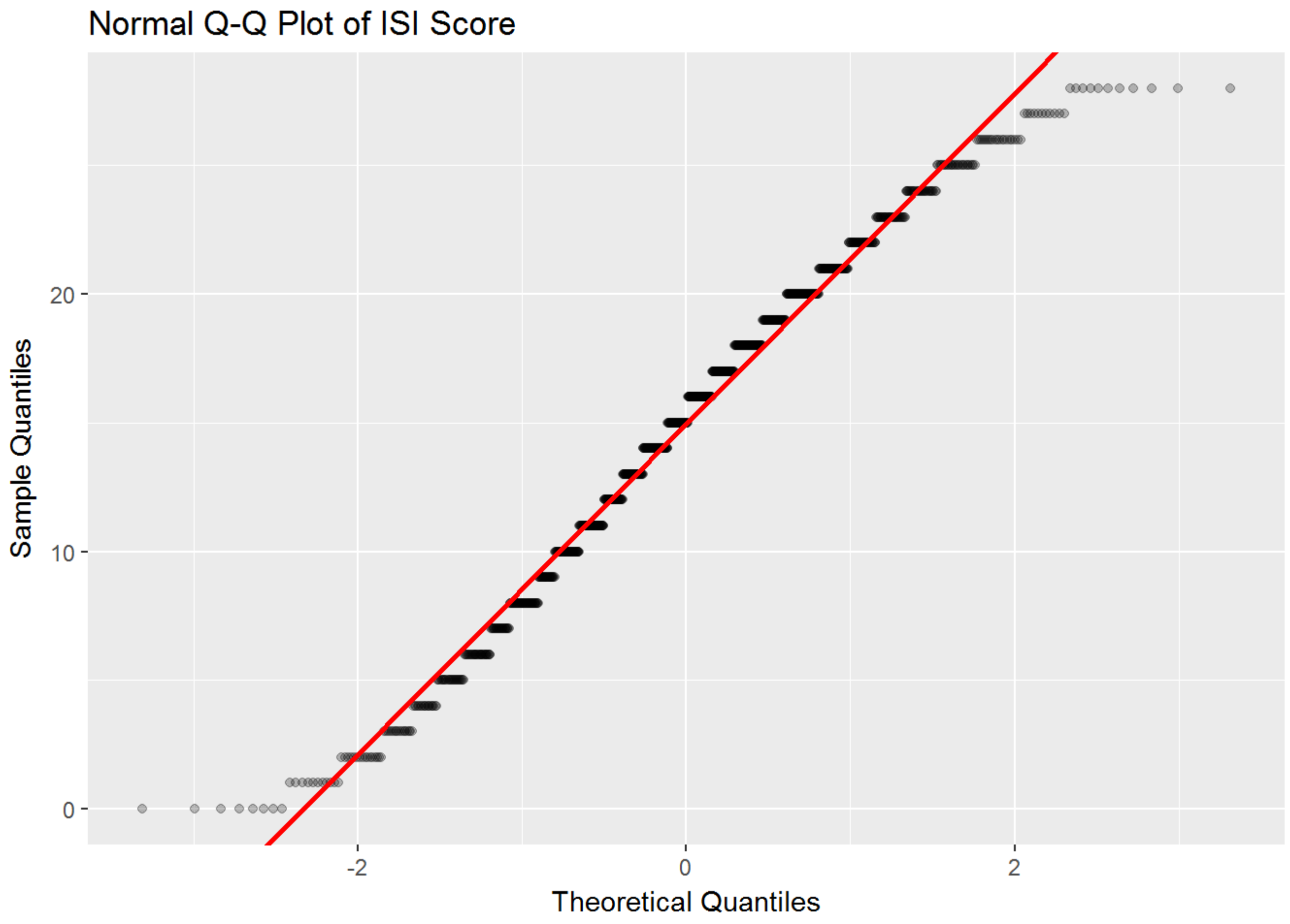

PSQI Score

Min	Median	Mean	Max	St.Dev	No.Data
1	10	9.967	21	4.109	105

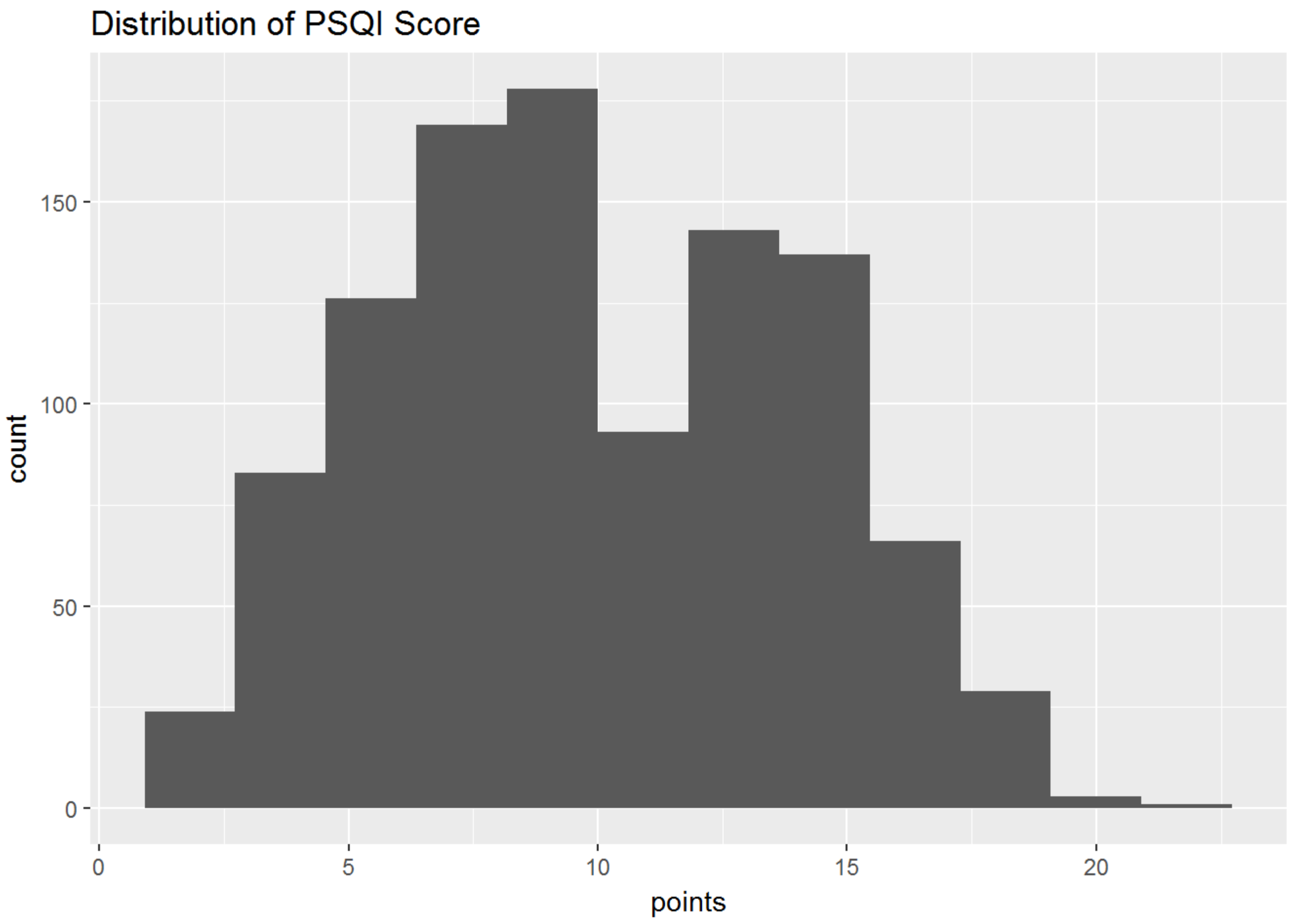

Distribution of PSQI Score
count
150
100
50
0
0
5
10
15
20
points

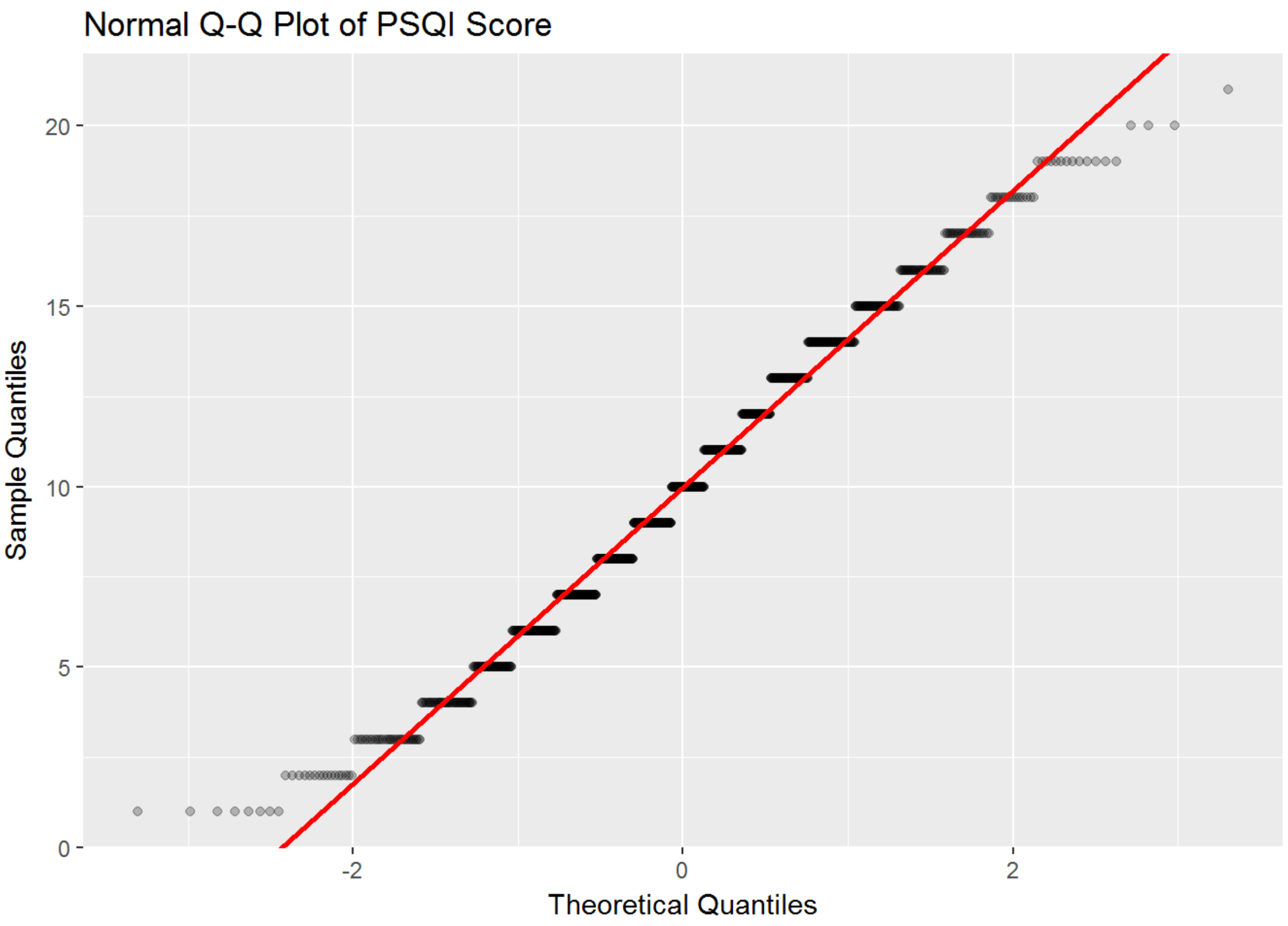

Normal Q-Q Plot of PSQI Score
Sample Quantiles
20
15
10
5
0
-2
0
2
Theoretical Quantiles

RLS Score

Min	Median	Mean	Max	St.Dev	No.Data
0	15	16.51	39	7.396	38

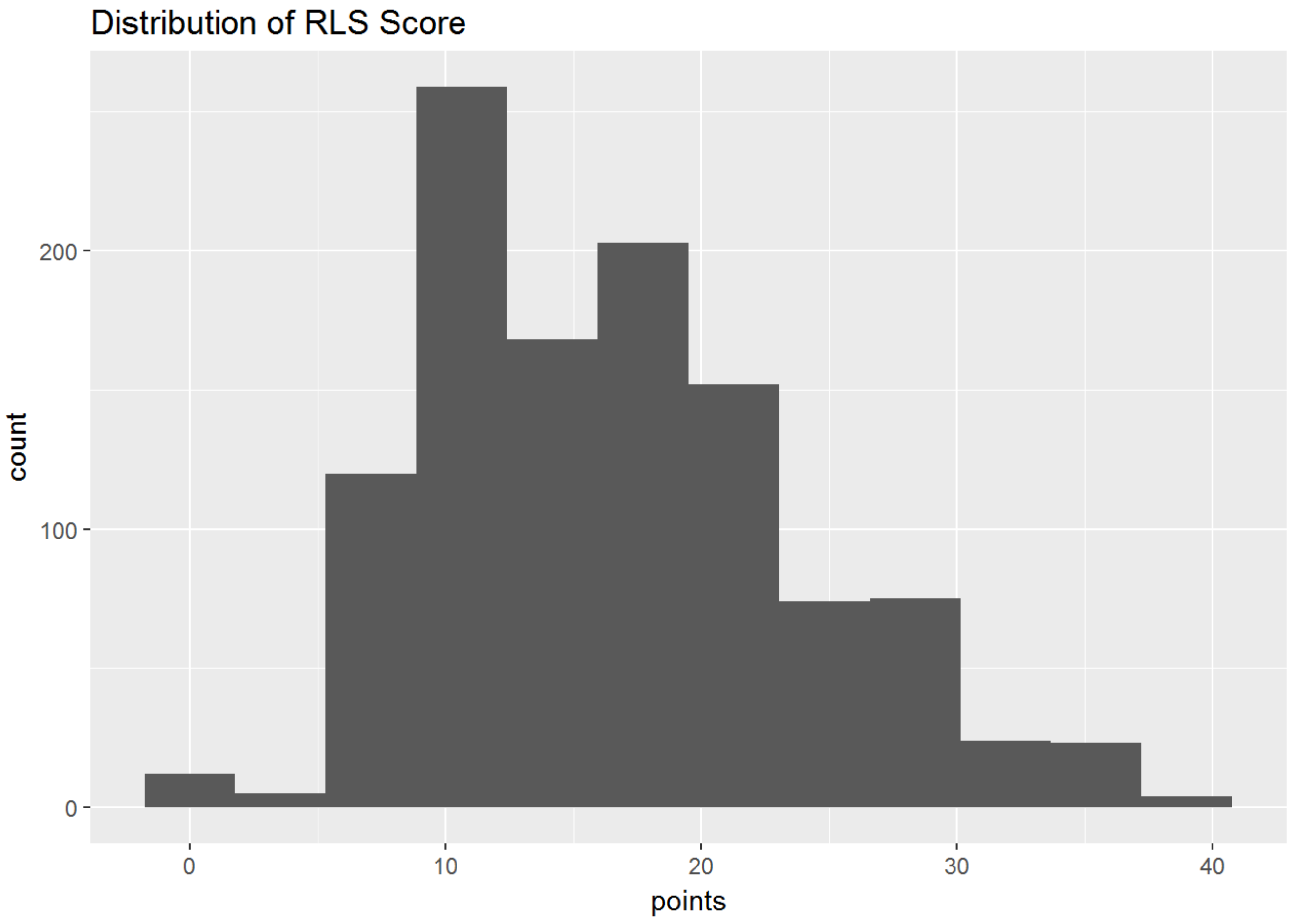

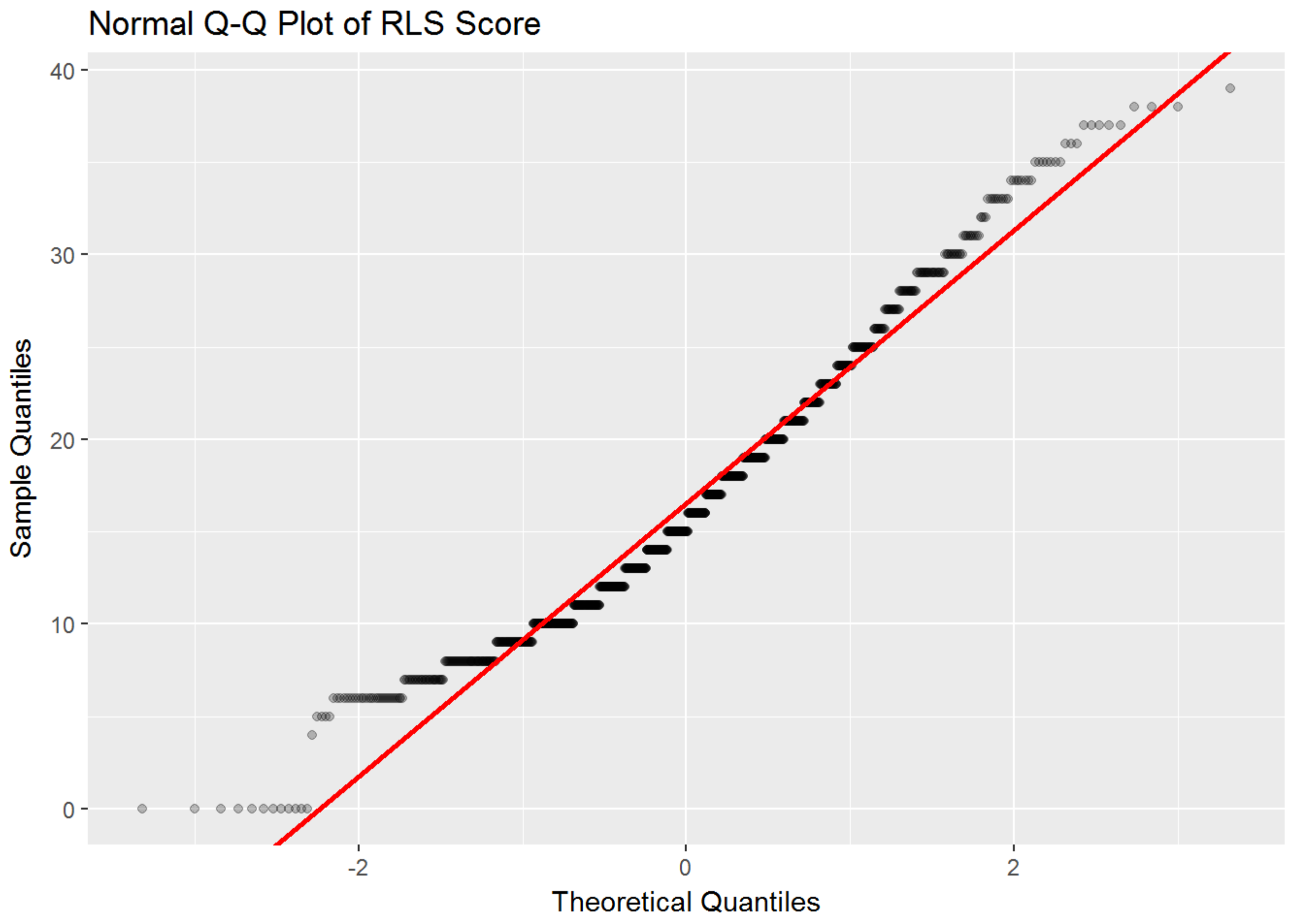

All Pairwise Correlations

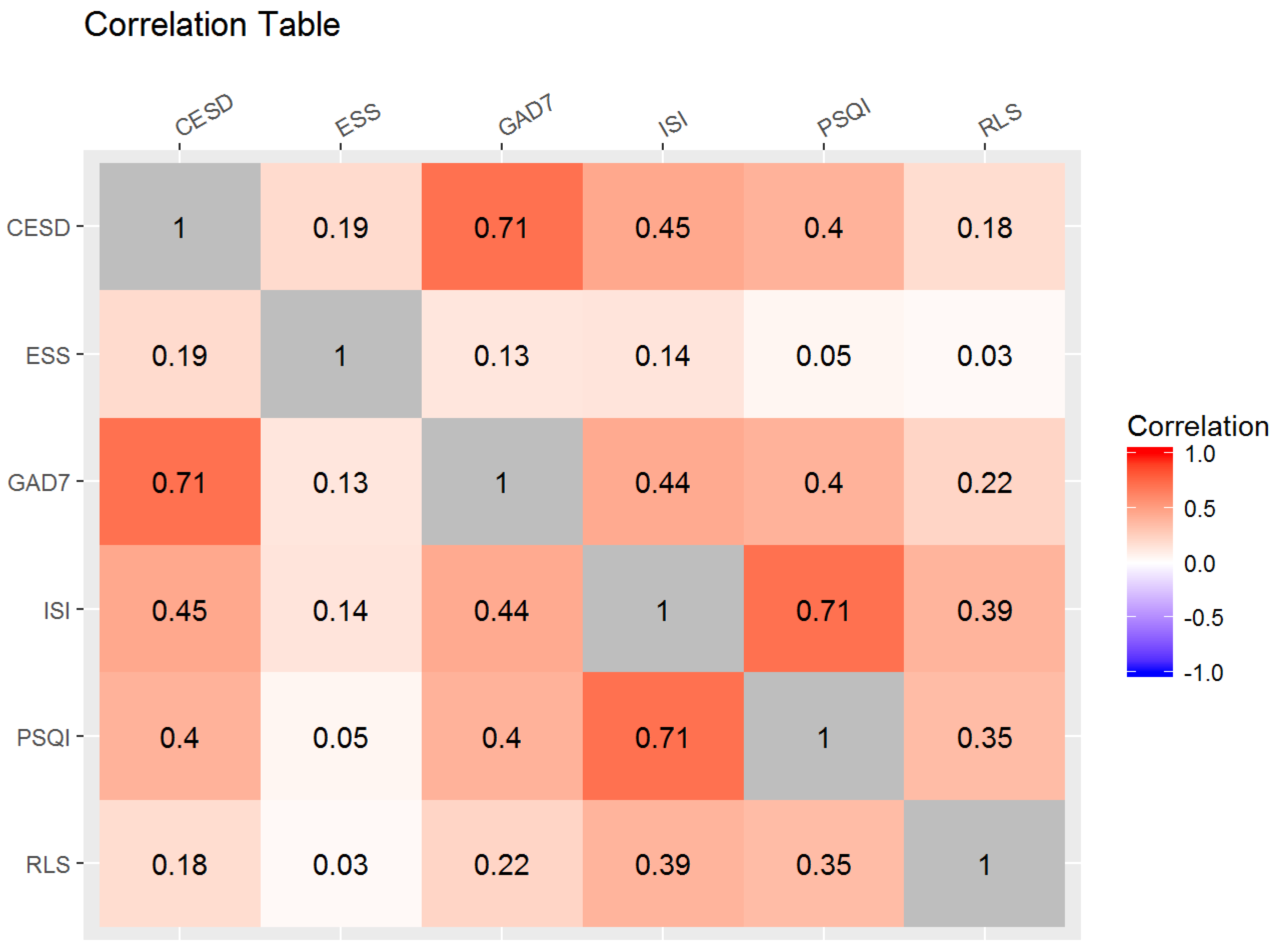

Principal Components, full sample

Total Patients with full data = 939

Explained Variance

Principal components space can only be explored for complete data, N = 939.

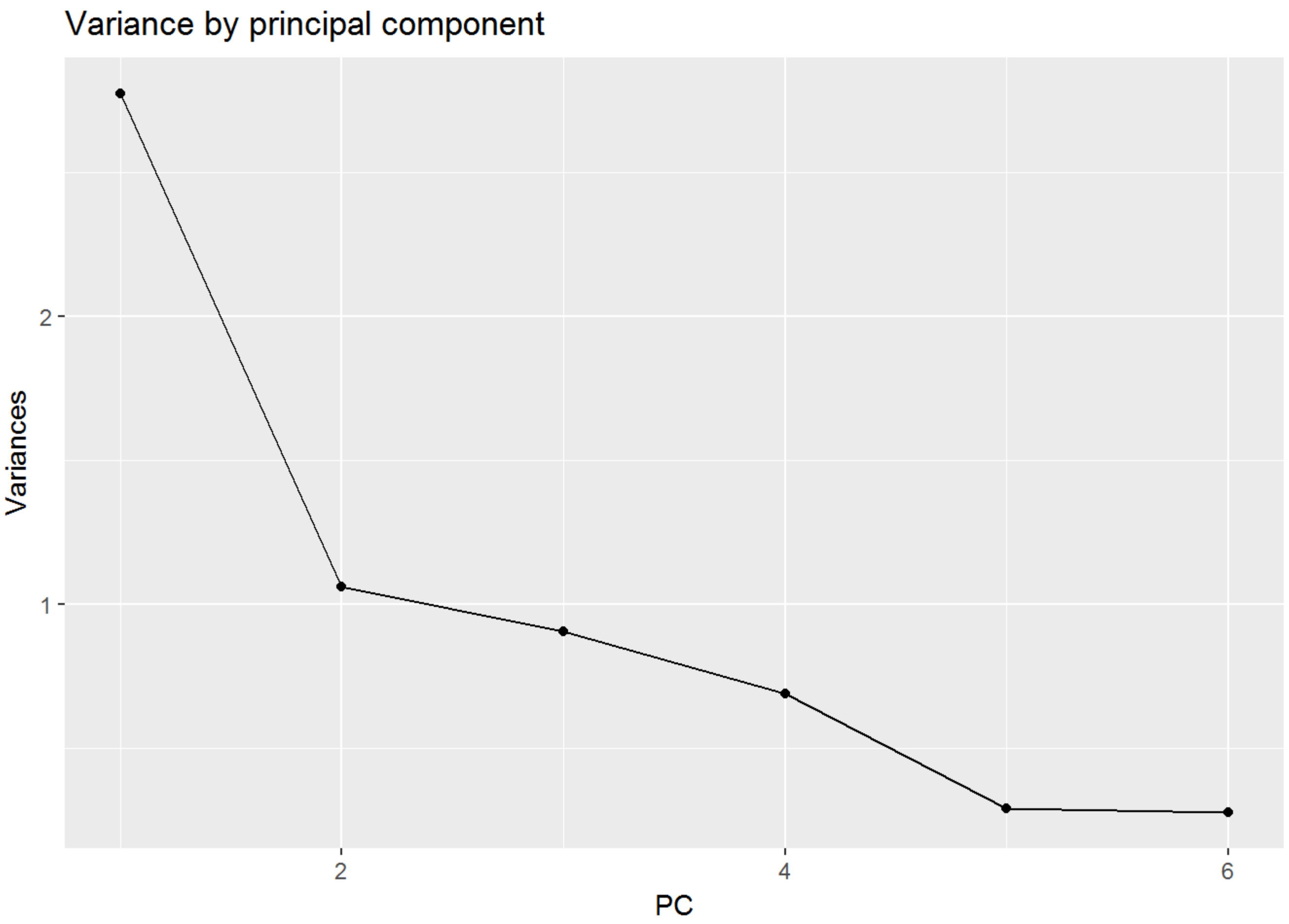

	PC1	PC2	PC3	PC4	PC5	PC6
**Standard deviation**	1.666	1.03	0.9522	0.8304	0.5403	0.5257
**Proportion of Variance**	0.4626	0.1767	0.1511	0.1149	0.04865	0.04605
**Cumulative Proportion**	0.4626	0.6392	0.7904	0.9053	0.9539	1

Component Loadings

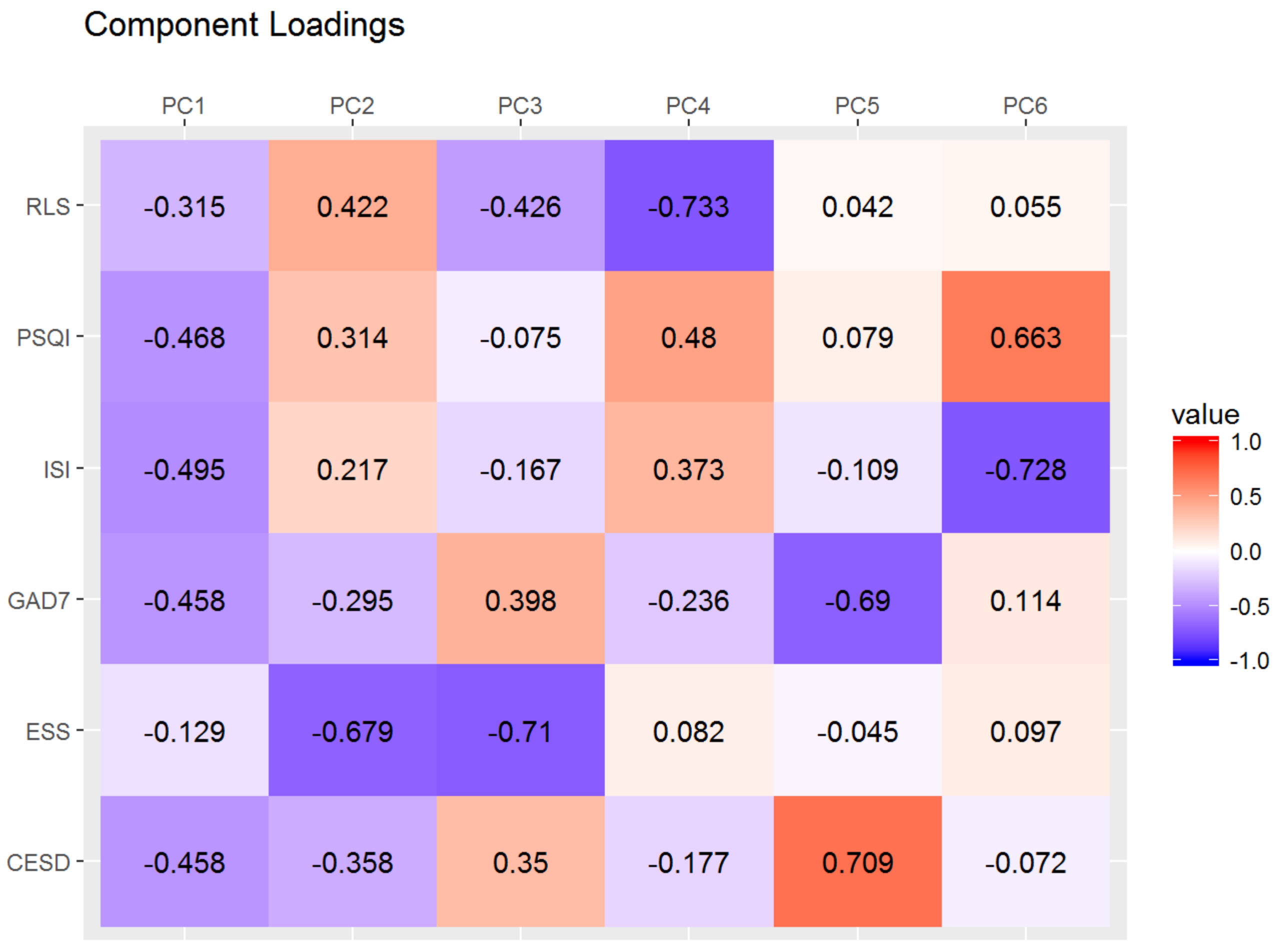

Component and Variable Correlations

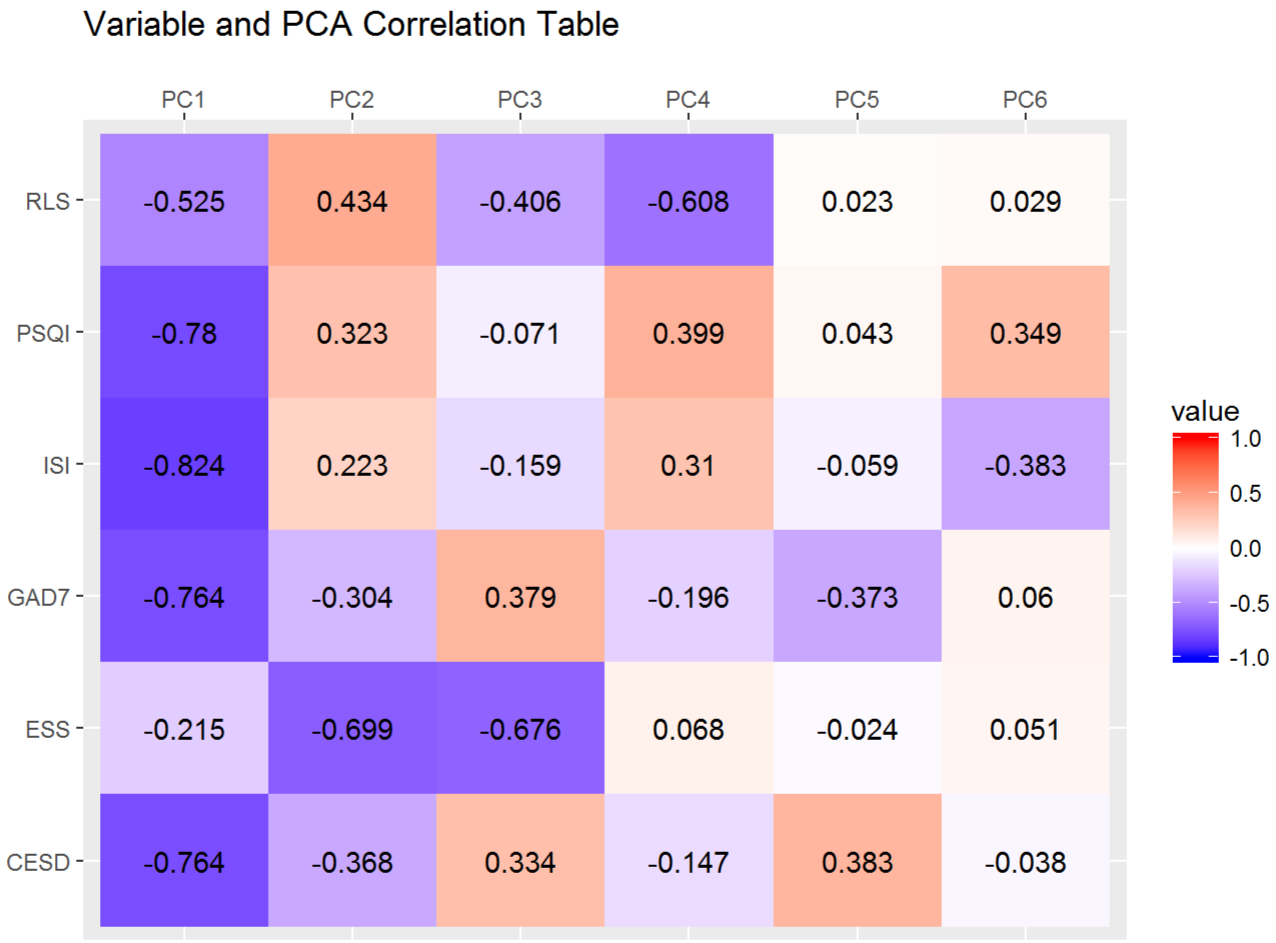

Variable loadings plotted in component space

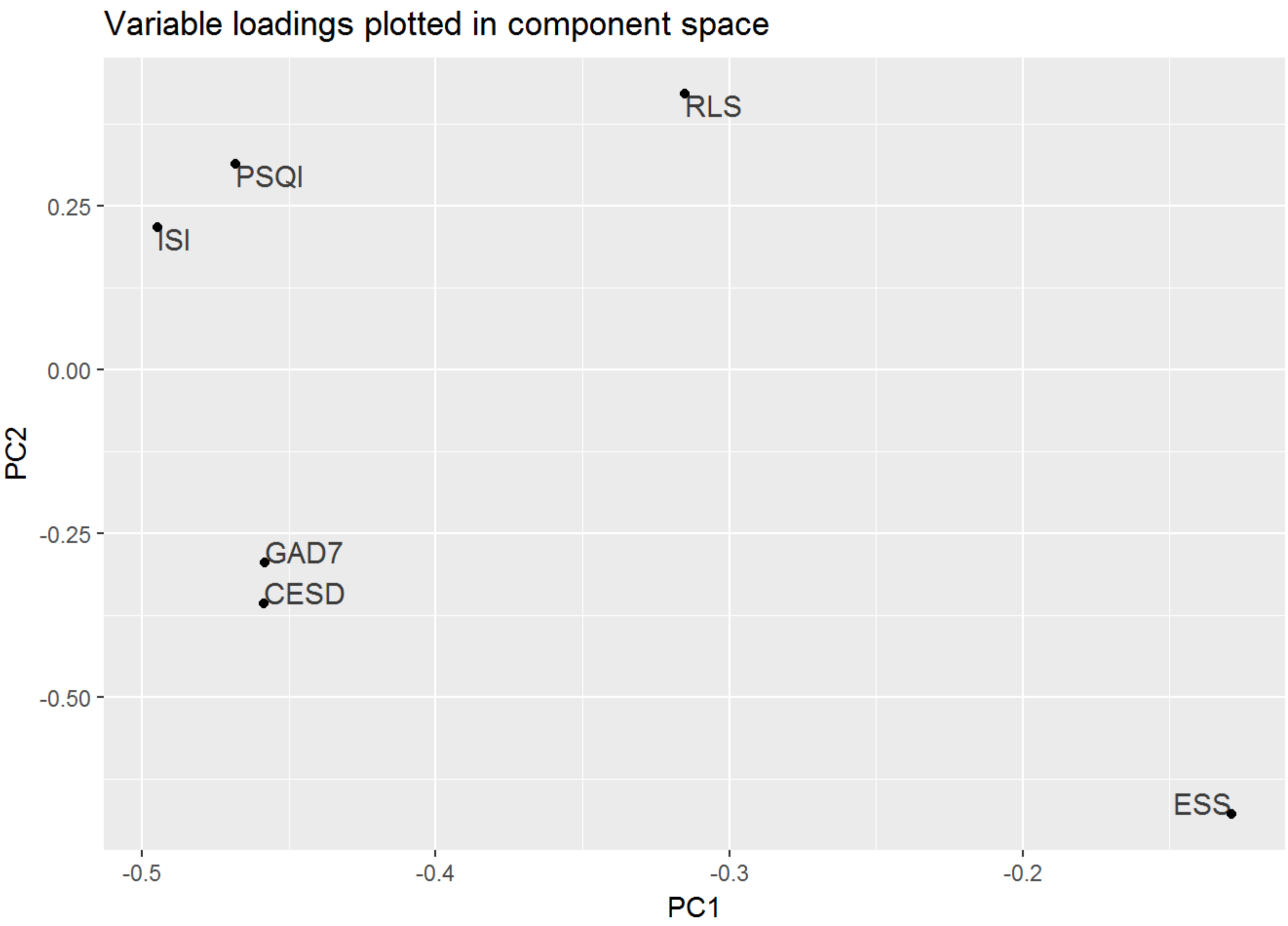

Varimax Rotation

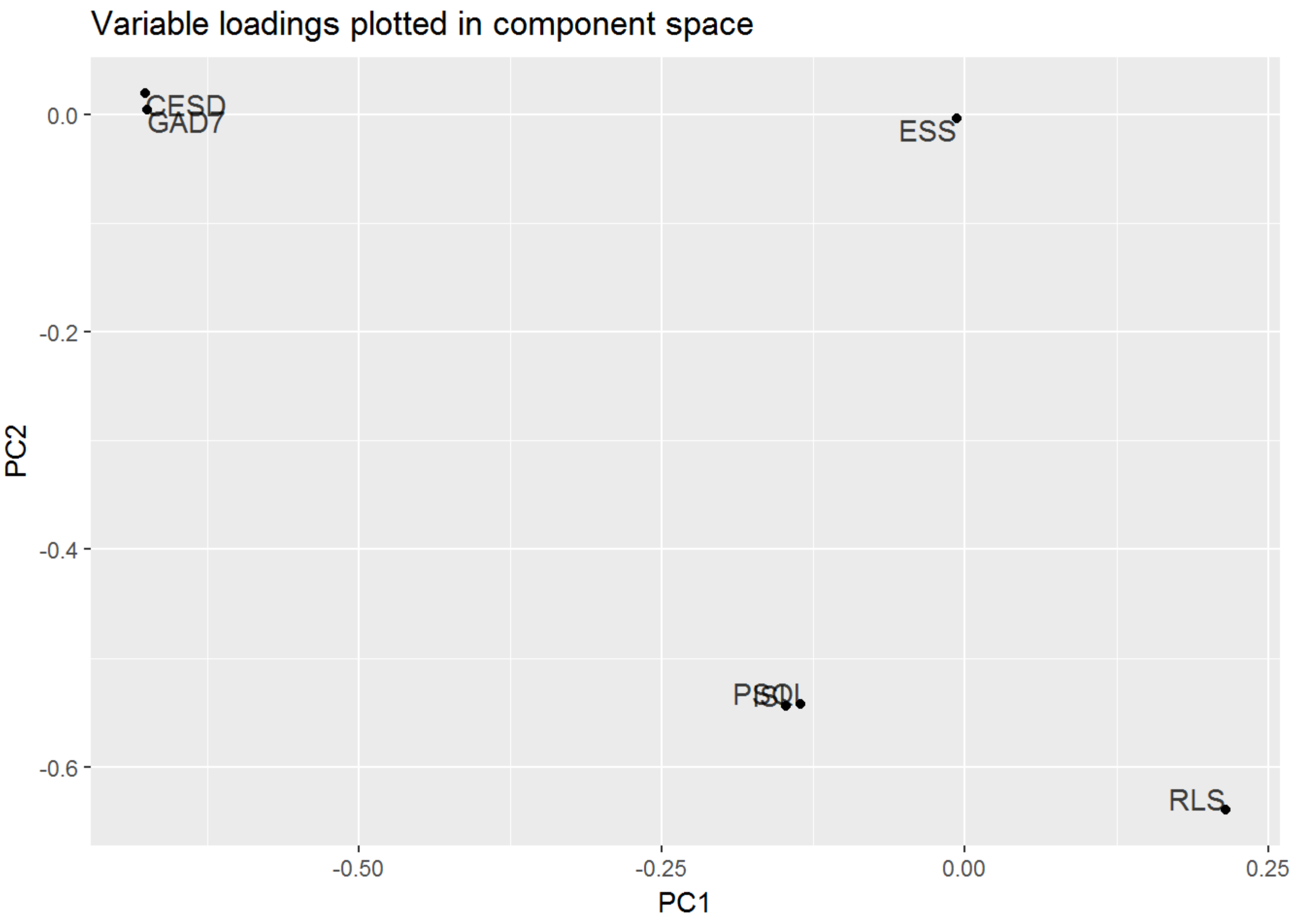

	PC1	PC2	PC3
**ESS_SCORE**	-0.006436	-0.003735	-0.9909
**CESD_SCORE**	-0.6768	0.01994	-0.04949
**GAD7_SCORE**	-0.6747	0.004802	0.02742
**ISI_SCORE**	-0.1481	-0.5445	-0.03235
**PSQI_SCORE**	-0.136	-0.5422	0.1038
**RLS_SCORE**	0.2152	-0.6396	-0.05597
